# Tetraarylphosphonium Cations with Excellent Alkaline‐Resistant Performance for Anion‐Exchange Membranes

**DOI:** 10.1002/cssc.202402366

**Published:** 2025-01-16

**Authors:** Ryoyu Hifumi, Yoshikazu Toyama, Keisuke Ikeda, Tetsuaki Hashimoto, Tomohiro Imai, Shinsuke Inagi, Ikuyoshi Tomita

**Affiliations:** ^1^ Department of Chemical Science and Engineering School of Materials and Chemical Technology Institute of Science Tokyo Nagatsuta-cho 4259-G1-9 Midori-ku, Yokohama 226-8501 Japan

**Keywords:** Alkaline stability, Anion exchange membranes, Fuel cells, Tetraarylphosphoniums, Water splitting

## Abstract

To realize the robust anion exchange membrane (AEM)‐based water splitting modules and fuel cells, the design and synthesis of tetraarylphosphonium (TAP) cations are described as a new class of cationic building blocks that exhibit remarkable alkaline stability under harsh conditions. TAP cations with highly sterically demanding aromatic substituents were efficiently synthesized from triarylphosphine derivatives and highly reactive arynes, whose alkaline degradation proved to be suppressed dramatically by the sterically demanding substituents. In the case of bis(2,5‐dimethylphenyl)bis(2,4,6‐trimethylphenyl)phosphonium, for example, approximately 60% of the cation survived for 27 d under the forced conditions (i.e., in 4 M KOH/CD_3_OH at 80 °C), while tetraphenylphosphonium degraded completely within 10 min in 1 M KOH/CD_3_OH at that temperature. Through the decomposition of the alkaline‐stable TAP cations, not only triarylphosphine oxides, which are often reported to form *via* the nucleophilic attack toward the cationic phosphorus center, but also triarylphosphines were detected, which suggested the presence of other degradation mechanisms due to the sterically demanding aromatic substituents. In kinetic analyses, bis(2,5‐dimethylphenyl)bis(2,4,6‐trimethylphenyl)phosphonium was found to exhibit 52 times higher stability compared to benzyltrimethylammonium, which is often employed as the cationic building block for AEMs.

## Graphical abstract

As new cationic building blocks potentially applicable to anion exchange membranes, the design and synthesis of tetraarylphosphoniums (TAPs) with highly sterically demanding aromatic substituents and their remarkable alkaline stability are described.

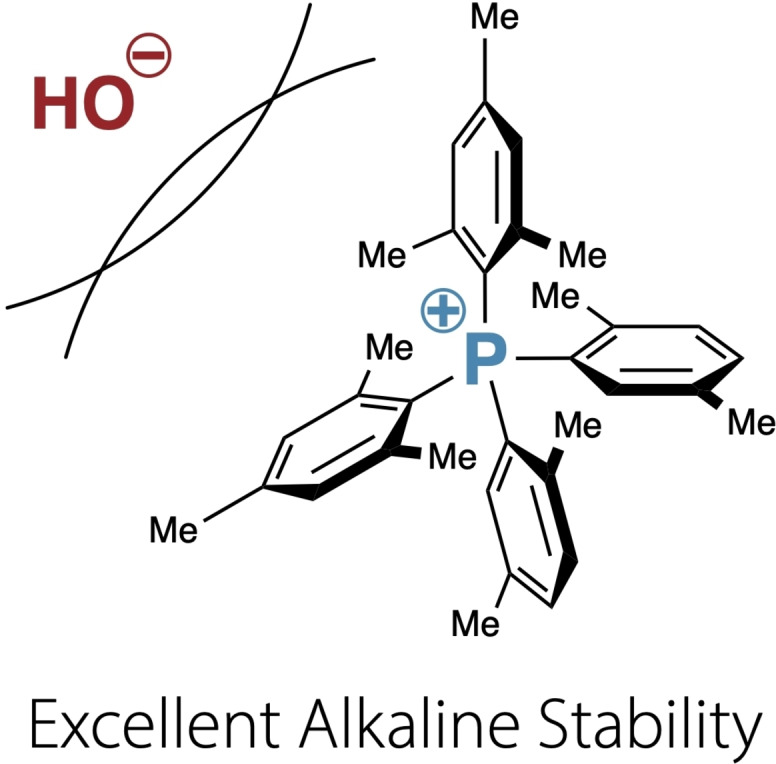

## Introduction

Alkaline fuel cells and water electrolysis modules using anion exchange membranes (AEMs) have attracted much attention because of their expectation to realize low‐cost hydrogen‐based energy conversion systems without the use of noble metal catalysts.[^1^, ^2^, ^3^, ^4^] So as to facilitate the AEM‐based modules, it is important to develop robust AEM materials that are totally intact under the harsh operating conditions, i.e., at higher temperature, in stronger alkaline media, and under electric field that generates active species. That is, besides the durability of the polymer backbones as is also the case of the proton exchange membrane systems, that of the cationic functional groups attached to the polymers should be paid much attention since many preceding studies pointed out the degradation of the cationic functional groups.[^5^, ^6^, ^7^]

**Figure 1:**
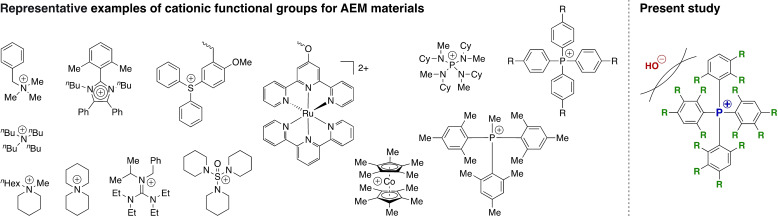
Representative examples of previously reported cationic functional groups for AEM materials and the molecular design of the present study.[^6^, ^7^, ^12^, ^34^, ^36^, ^41^, ^42^, ^43^, ^45^, ^47^, ^49^, ^51^, ^56^]

Benzyltrimethylammonium (**BTMA**) has been often studied as a cationic building block for AEMs (Figure 1),[^4^, ^5^, ^7^, ^8^, ^9^, ^10^, ^11^, ^12^, ^13^] while it gradually degrades under forced conditions by means of the nucleophilic substitution on the *α*‐carbons of the nitrogen.[^7^, ^11^] For example, **BTMA** degrades approximately 90% in 1 M KOH/CD_3_OH at 80 °C for 30 d,^[7]^ and a polymeric membrane containing **BTMA** moieties also loses more than 50% of its ion exchange capacity after immersion in a 1 M NaOH aqueous solution at 80 °C for 30 d.^[14]^

The alkaline stability of cationic functional groups has been studied to improve the robustness of the AEM materials.[^5^, ^6^, ^7^, ^15^, ^16^, ^17^] Concerning nitrogen‐based cations, open‐chain,[^11^, ^12^, ^18^, ^19^, ^20^, ^21^] cyclic,[^5^, ^6^, ^9^, ^12^, ^22^, ^23^, ^24^, ^25^, ^26^] bicyclic,[^6^, ^9^, ^12^, ^27^] and spirocyclic alkyl‐substituted ammoniums,[^12^, ^24^, ^28^, ^29^] tetraaryl‐substituted ammoniums,[^30^, ^31^, ^32^] guanidiniums,[^33^, ^34^] and imidazoliums[^6^, ^35^, ^36^, ^37^, ^38^, ^39^, ^40^] have been investigated and their molecular design proved to be effective to improve their alkaline stability (Figure 1). As cationic functional groups, cations of other elements such as sulfur (triarylsulfoniums,^[41]^ tris(dialkylamino)sulfoniums, and tris(dialkylamino)sulfoxoniums^[42]^) and transition metals (cationic terpyridine complexes of Ru, Ni, and Co[^43^, ^44^] and cobaltoceniums[^45^, ^46^]) were also studied besides phosphorus‐containing cations (Figure 1).

Noonan *et al*. have focused on a tetrakis(dialkylamino)phosphonium cation and prepared a polyethylene derivative functionalized with the corresponding moieties as AEM materials, which showed no significant changes in hydroxide conductivity after immersion in 15 M KOH aqueous solution at 22 °C for 140 d (Figure 1).^[47]^ Yan and coworkers reported the synthesis, alkaline stability, and AEM performance of a poly(ether sulfone)‐based ionomer with a benzyltris(2,4,6‐trimethoxyphenyl)phosphonium structure.^[48]^ They also investigated the alkaline stability of various monoalkyltriarylphosphonium cations, where methyltris(2,4,6‐trimethylphenyl)phosphonium cation was found to have the most excellent alkaline stability (<20% degradation after 5000 h at 80 °C in 1 M KOD CD_3_OD/D_2_O (5/1 vol.)) (Figure 1).[^49^, ^50^]

Tetraarylphosphoniums (TAPs) are expected to serve as another promising motif for the alkaline‐stable cations, because the TAP cations, unlike phosphonium cations with one or more aliphatic substituents, are inert to S_N_2‐ and E2‐type reactions and ylide formation. Previously, several examples on the synthesis and evaluation of polymers containing TAP units have been reported. For example, Smith and coworkers synthesized TAP‐containing polymers by the Ni‐catalyzed coupling reactions of aryl bromides/triflates and triarylphosphines, in which some of the polymers proved to be stable in 6 M NaOH (aq) at 65 °C for at least 24 h.[^51^, ^52^, ^53^] A poly(ether) with tetraphenylphosphonium moieties has been applied to composite anion exchange membranes with polysulfone, which shows less than a 2% decrease in ionic conductivity after immersion in a 1 M NaOH solution at ambient temperature for 300 h.^[54]^ Miller and coworkers synthesized arylene ether ketone/sulfone‐type polymers containing tetraphenylphosphonium moieties from bis(hydroxyphenyl)diphenylphosphonium salts and 4,4’‐difluorobenzophenone or 4,4’‐difluorodiphenylsulfone, whose films exhibit no degradation after immersion in a 6 M NaOH solution at 65 °C for 48 h.^[55]^ Wang and coworkers reported the synthesis of a series of poly(phosphazene)s bearing TAP moieties having electron‐donating *para* substituents, where the membrane containing *p*‐methoxy‐substituted TAP moieties showed higher stability (7.6% decrease in ionic conductivity after immersion in 1 M KOH (aq) at 60 °C for 10 d) in comparison with that containing unsubstituted ones (21.8%).^[56]^

Although these examples may suggest that TAP is an attractive building block to achieve an alkaline‐stable cationic functional group, at least within the authors’ knowledge, its systematic molecular design has scarcely been studied yet. So as to design robust AEM materials based on TAP building blocks, it is essentially important to carry out studies on the structure‐stability relationship of a variety of substituted TAP cations. Since the alkaline decomposition of tetraphenylphosphonium (**1a**) cation has been reported to take place by the nucleophilic attack of the anion (hydroxide/alkoxide) to the cationic phosphorus center,[^56^, ^57^, ^58^] it is expected that the alkaline stability of TAP cations would be improved by increasing the steric bulkiness around the phosphorus centers. However, the synthetic tools of TAP building blocks have been limited compared to those of phosphonium cations containing aliphatic substituents. The use of the aryne chemistry would be a rational approach for the designed synthesis of TAP building blocks. Wittig *et al*. and independently Rémond *et al*. have reported that the reaction of triarylphosphines with arynes proceeds under mild conditions to produce aryl‐substituted phosphoniums.[^59^, ^60^] In the report of Rémond *et al*., they briefly described the synthesis of a few TAP salts with sterically demanding substituents such as (phenyl)tri(*o*‐tolyl)phosphonium triflate (**2a**•OTf) by the reaction of tri(*o*‐tolyl)phosphine with 2‐trimethylsilylphenyl triflate, besides simple TAP salts such as tetraphenylphosphonium triflate (**1a**•OTf), while their alkaline stability has not been commented. Lopez‐Leonardo *et al*. have described that the reaction of triarylphosphine sulfides likewise proceeds to give arylthioaryl‐substituted phosphoniums.^[61]^ With expectation that these aryne‐based reactions would take place also in the presence of more sterically demanding substituents, we describe the synthesis and alkaline‐resistant performance of various TAP cations with sterically demanding substituents (Figure 1).

## Results and Discussion

### Synthesis of TAP Salts

**Scheme 1:**
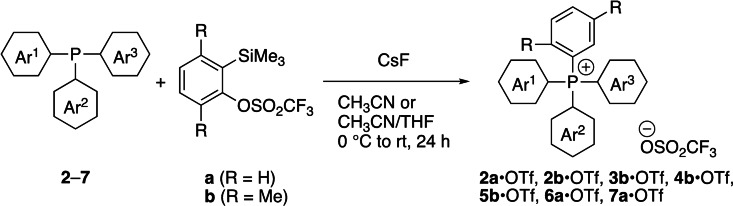
Synthesis of TAP salts by reaction of phosphines with arynes. THF=tetrahydrofuran.

Following the reaction conditions for **2a**•OTf,^[60]^ triarylphosphines (**2**–**7**) were subjected to the reaction with an aryne precursor possessing 3,6‐dimethyl substituents (**b**) to obtain the corresponding TAP salts (Scheme 1 and Table 1). The triarylphosphines with plural methyl substituents (**2**–**5**) afforded the phosphonium salts (**2b**•OTf–**5b**•OTf) in excellent yields (92–98%), after the purification by silica gel column chromatography. On the other hand, tris(2,6‐dimethylphenyl)phosphine and tris(2,4,6‐trimethylphenyl)phosphine (**6** and **7**, respectively) did not afford the objective TAP salts by the reaction with the precursor (**b**) under the examined conditions, presumably due to their large steric hindrance around the phosphorus atoms. Instead, the phosphonium salts (**6a**•OTf and **7a**•OTf) were obtained in very good to excellent yields (91 and 88%, respectively) by the reaction with a less sterically demanding precursor (**a**). Although the results obtained from the series of experiments might indicate the limitation of the combinations of the aryl substituents, the authors would like to emphasize that the TAP salts with high steric defense are now in hand, which have never been accessible previously by the other synthetic methods.

**Table 1:** Structures and yields of TAP salts.

Phosphines/phosphine sulfides	Aryne precursors	TAP salts (isolated yields)
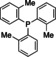 **2**	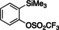 **a**	**2a**•OTf^[a]^
**2**	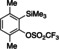 **b**	**2b**•OTf (92%)
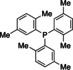 **3**	**b**	**3b**•OTf (94%)
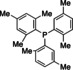 **4**	**b**	**4b**•OTf (98%)
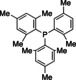 **5**	**b**	**5b**•OTf (97%)
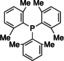 **6**	**a**	**6a**•OTf (91%)
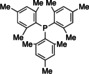 **7**	**a**	**7a**•OTf (88%)
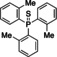 **8**	**a**	**8a**•OTf (81%)^[b]^

[a] See, ref [60]. [b] Reaction scheme, see the Supporting Information.

In addition, based on the reaction of triarylphosphine sulfides with arynes to give arylthioaryl‐substituted phosphonium salts,^[61]^ tri(*o*‐tolyl)phosphine sulfide (**8**) was reacted with the precursor (**a**) to produce a TAP salt carrying the *ortho* arylthio group (**8a**•OTf) in 81% yield (the synthetic scheme is shown in the Supporting Information).

All the TAP salts thus obtained were characterized by the ^1^H, ^19^F, and ^31^P nuclear magnetic resonance (NMR) spectroscopy and mass spectrometry (details: see the Supporting Information). In each ^31^P NMR spectrum of the TAP salts (**2a**•OTf, **2b**•OTf, **3b**•OTf, **4b**•OTf, **5b**•OTf, **6a**•OTf, **7a**•OTf, and **8a**•OTf), the phosphorus atom in the TAP cations appeared in the range of 14.5–23.4 ppm. In their ^19^F NMR spectra, only triflate anion (−77.9 to −78.0 ppm) derived from the aryne precursors was observed as the counter anion, and no peak for fluoride anion (around −150 ppm),[^62^, ^63^] which might be derived from CsF, was detected.

### Degradation Behavior of TAP Cations

The alkaline degradation behavior of the present TAP cations was evaluated in KOH/CD_3_OH solutions, following the conditions employed for the alkaline degradation experiments of several organic cations.^[6]^ That is, CD_3_OH was used instead of CD_3_OD to avoid any hydrogen/deuterium exchange processes, which may result in the complex NMR spectra, and neither H_2_O nor H_2_O/CD_3_OH was used since the degradation of the organic cations takes place faster in non‐aqueous media, allowing the observation in the shorter period of time.^[5]^ The progress of the degradation of the TAP cations and the decomposition products were investigated by the ^31^P NMR spectroscopy and gas chromatography/mass spectrometry (GC/EI‐MS).

**Figure 2:**
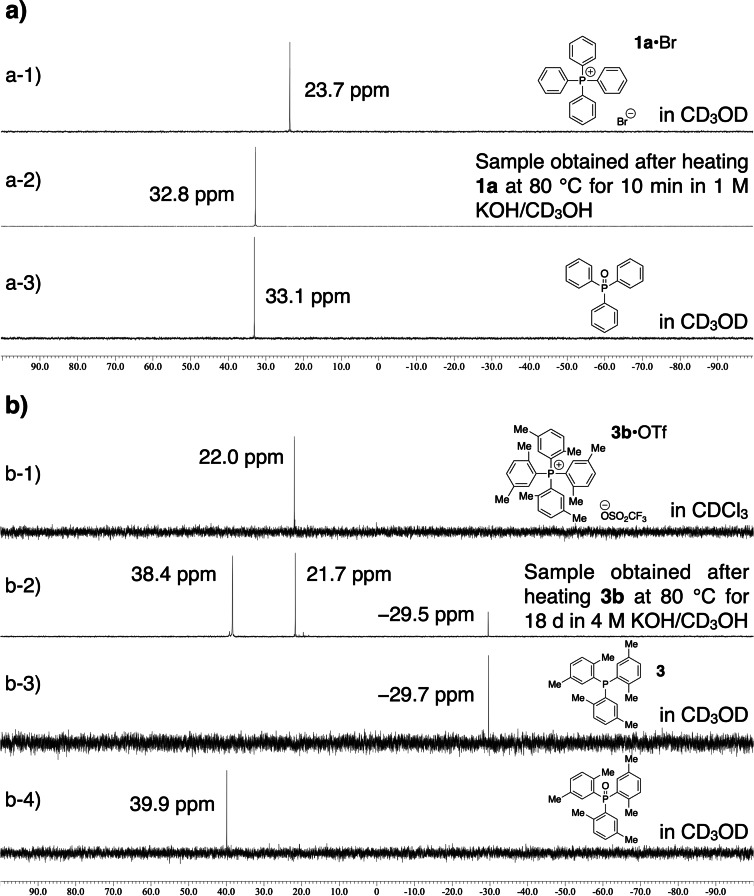
a) Stacked ^31^P NMR spectra of a‐1) **1a**•Br in CD_3_OD, a‐2) the sample obtained after heating **1a** at 80 °C for 10 min in 1 M KOH/CD_3_OH, and a‐3) triphenylphosphine oxide in CD_3_OD. b) Stacked ^31^P NMR spectra of b‐1) **3b**•OTf in CDCl_3_, b‐2) the sample obtained after heating **3b** at 80 °C for 18 d in 4 M KOH/CD_3_OH, b‐3) tris(2,5‐dimethylphenyl)phosphine (**3**) in CD_3_OD, and b‐4) tris(2,5‐dimethylphenyl)phosphine oxide in CD_3_OD.

**Scheme 2:**
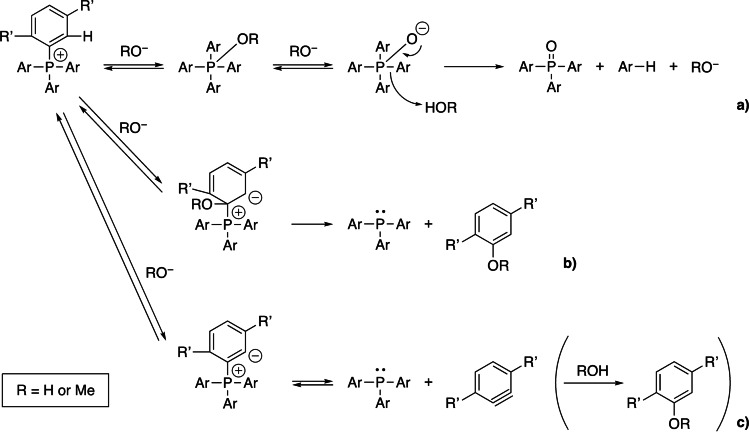
Possible mechanisms of the degradation of **1a** (R’=H, Ar=phenyl) and **3b** (R’=Me, Ar=2,5‐dimethylphenyl).

In the case of tetraphenylphosphonium (**1a**), complete degradation took place in 1 M KOH/CD_3_OH at 80 °C within 10 min, and a single peak attributable to the phosphorus atom of triphenylphosphine oxide was observed at 32.8 ppm in the ^31^P NMR spectrum (Figure 2a). As described in the introduction, the degradation of tetraphenylphosphonium cation in aqueous alkaline media takes place by the nucleophilic attack of hydroxide to the phosphorus atom to produce triphenylphosphine oxide. Accordingly, the degradation process operating under the present conditions would be the same as in the preceding studies (Scheme 2a, R’=H and Ar=phenyl).[^56^, ^57^, ^58^]

To suppress the degradation of the TAP cations by the nucleophilic attack toward the cationic phosphorus centers, the molecular design of the four aromatic substituents was carried out from the viewpoint of their steric factors. As expected, the introduction of sterically demanding aromatic substituents on the phosphorus atoms dramatically slowed down the alkaline degradation as described in detail in the following kinetic studies. For example, approximately 35% of **3b** survived after heating at 80 °C for 18 d in 4 M KOH/CD_3_OH. In the ^31^P NMR spectrum of a sample obtained after being kept under these conditions, two peaks were newly observed at −29.5 ppm and 38.4 ppm, which are attributable to the phosphorus atoms of tris(2,5‐dimethylphenyl)phosphine (**3**) and its oxide, respectively (Figure 2b). In addition, in the GC/EI‐MS measurement of the sample obtained after the prolonged alkaline degradation test of **3b** (i.e., at 80 °C for 98 d), peaks with *m/z* values of 106, 139, 346, and 362 were observed, which are consistent with those of *p*‐xylene, 2,5‐dimethylanisole whose three hydrogen atoms were replaced with three deuterium atoms (2,5‐dimethylanisole‐*d*_3_, C_9_H_9_D_3_O), tris(2,5‐dimethylphenyl)phosphine (**3**), and tris(2,5‐dimethylphenyl)phosphine oxide, respectively (Figure S53). Tris(2,5‐dimethylphenyl)phosphine oxide and *p*‐xylene are likely to be produced *via* the degradation pathway shown in Scheme 2a (R’=Me and Ar=2,5‐dimethylphenyl), whereas tris(2,5‐dimethylphenyl)phosphine (**3**) and 2,5‐dimethylanisole‐*d*_3_ may be produced *via* the nucleophilic *ipso* attack of CD_3_O^−^ ion (Scheme 2b, R’=Me and Ar=2,5‐dimethylphenyl) and/or *via* the proton abstraction from the *ortho* position that leads to the formation of the aryne species (Scheme 2c, R’=Me and Ar=2,5‐dimethylphenyl). The results indicate that sterically demanding aromatic substituents are effective to suppress the degradation caused by the nucleophilic attack toward the cationic phosphorus center, which consequently allows us to detect other slow degradation pathways to produce triarylphosphines.

### Kinetic Analyses

**Figure 3:**
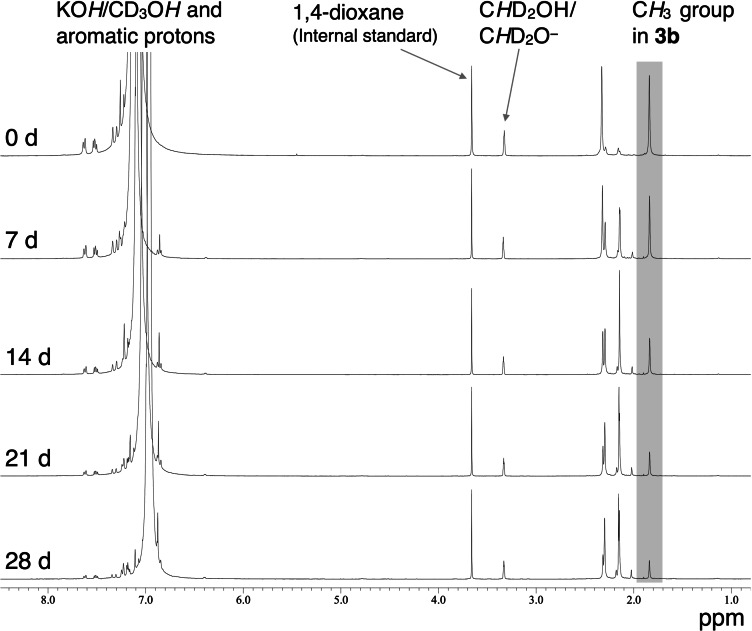
^1^H NMR spectra of **3b** over 28 d at 80 °C in a 4 M KOH/CD_3_OH containing 1,4‐dioxane as an internal standard.

**Figure 4:**
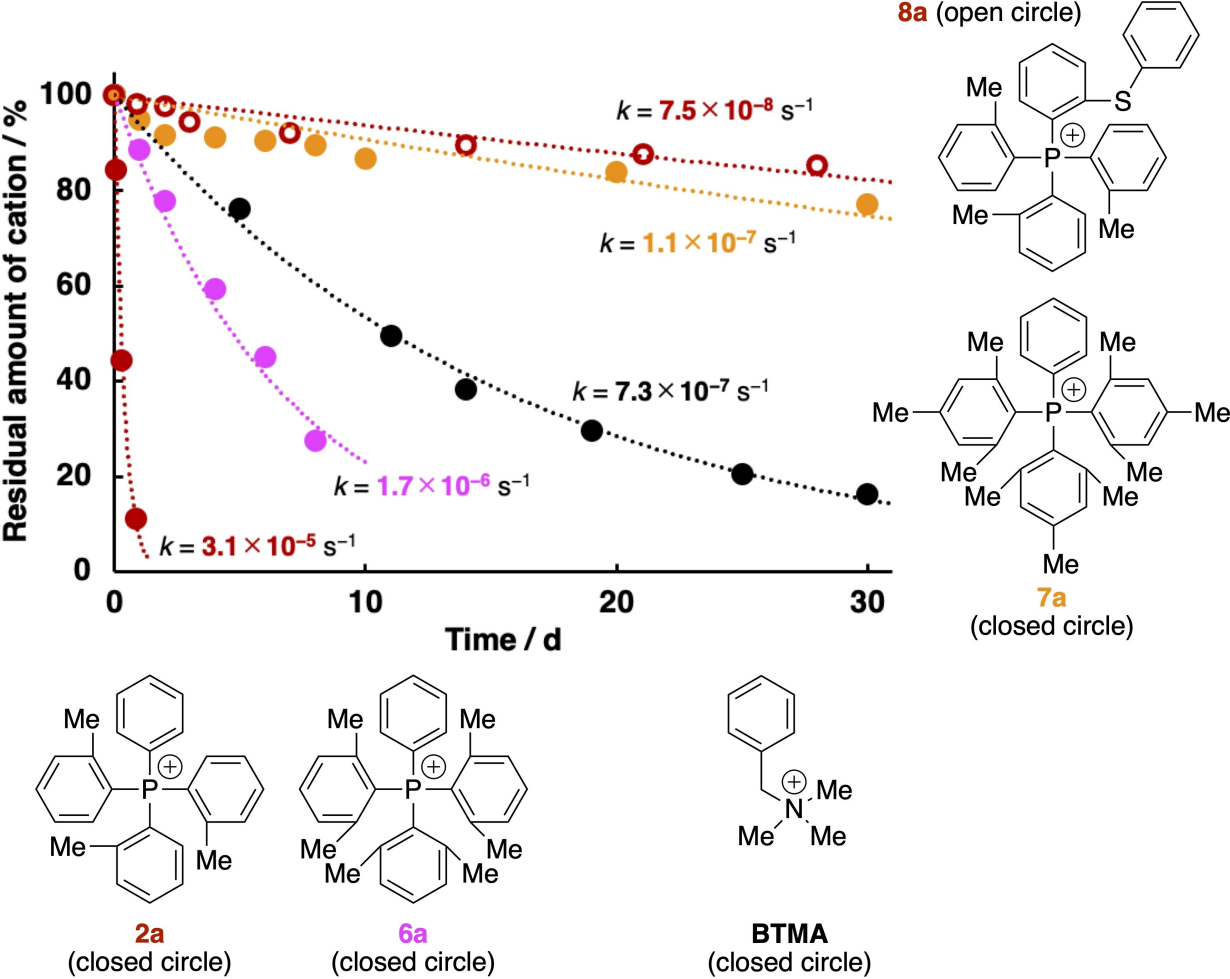
Degradation curves of TAP cations (**2a** and **6a**–**8a**) in 1 M KOH/CD_3_OH and **BTMA** in 1 M KOH/CD_3_OD at 80 °C. Circles represent measured values. Dotted lines were fitted using Equation (1).

**Figure 5:**
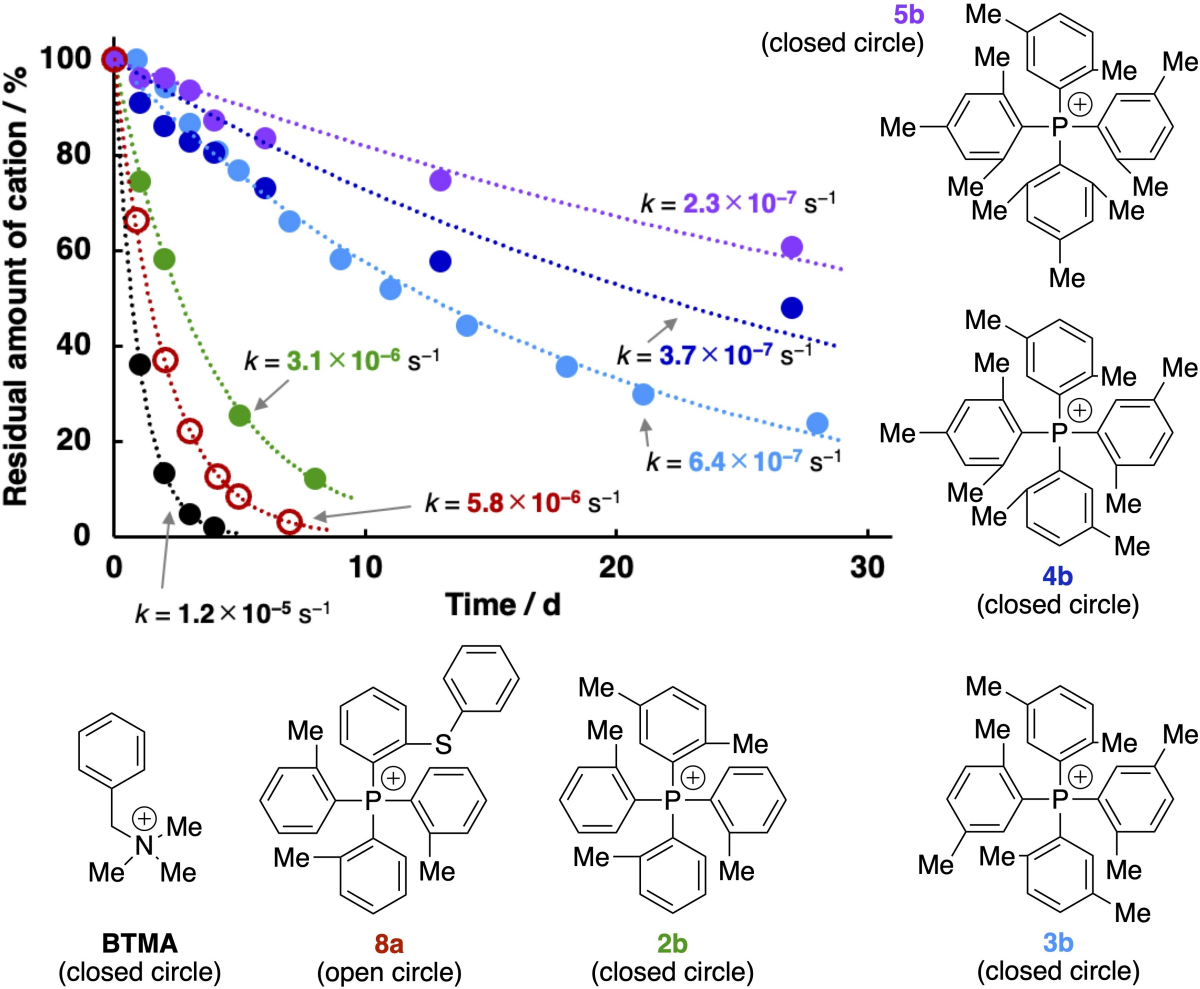
Degradation curves of TAP cations (**2b**–**5b** and **8a**) and **BTMA** in 4 M KOH/CD_3_OH at 80 °C. Circles represent measured values. Dotted lines were fitted using Equation (1).

The kinetic analyses of the alkaline degradation of the TAP cations were performed in the KOH/CD_3_OH solutions. The residual amounts of the TAP cations were determined by the ^1^H NMR spectroscopy, because the ^31^P NMR spectroscopy requires a longer measurement time for the quantitative evaluation due to the long *T*_1_ relaxation of the ^31^P nuclei of the TAP cations (Figures S54 and S55). The TAP salts (approximately 20 mg) were dissolved in 1 M or 4 M KOH/CD_3_OH solutions (0.6 mL) containing a small amount of 1,4‐dioxane as an internal standard to quantify the amounts of the TAP cations. The initial amounts of the TAP cations were determined by integrating the proton signals of the methyl groups relative to the internal standard in the ^1^H NMR spectra of the freshly prepared samples (0 d in Figure 3). The samples were then heated at 80 °C in an oil bath and the degradation of the TAP cations was followed by the ^1^H NMR spectra (Figure 3). The residual amounts of the TAP cations thus obtained are plotted against time in Figures 4 and 5 for the 1 and 4 M KOH solutions, respectively.

At present, the detailed degradation mechanism of the TAP cations and the order of the overall reaction kinetics are not fully understood, as mentioned above. However, the degradation of the TAP cations would follow first‐order kinetics with respect to their concentrations, and the concentration of the nucleophiles (i.e., hydroxide and methoxide) can be assumed to be constant since the excess amount of KOH was used in the alkaline stability tests performed in the present study. Therefore, the degradation rate is most likely expressed as
(1)

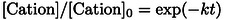




where [Cation], [Cation]_0_, *t*, and *k* are the concentration of the cations (TAP or **BTMA**), the initial concentration of the cations, time, and a rate constant, respectively. The obtained degradation curves can be fitted well by Equation (1), as indicated by the dashed lines in Figures 4 and 5, and the rate constants (*k* values) are also shown there (the plots of [Cation]_0_/[Cation] on logarithmic scales versus time are shown in Figures S56 and S57).

In a series of the TAP cations (**1a**, **2a**, and **6a**), more than 50% of **2a** and **6a** degraded within 7 h and 6 d, respectively, in 1 M KOH/CD_3_OH at 80 °C (Figure 4), although they are more stable than **1a**, which degraded completely within 10 min under the same conditions. The alkaline stability is in the order of **1a**<**2a**<**6a**, which may indicate that the alkaline stability of the TAP cations can be improved by introducing the methyl groups at the *ortho* positions. In addition, the introduction of the methyl groups at the *para* positions also appeared to stabilize the TAP cations. Namely, **7a** degraded only 23% in 1 M KOH/CD_3_OH at 80 °C for 30 d and has a *k* value of 1.1×10^−7^ s^−1^, while **6a** without the methyl groups at the *para* positions has a *k* value of 1.7×10^−6^ s^−1^.

The TAP cation with an arylthio group in the *ortho* position (**8a**) degraded only 15% in 1 M KOH/CD_3_OH at 80 °C for 28 d and has a *k* value of 7.5×10^−8^ s^−1^. Compared to **2a** without the arylthio group (*k*=3.1×10^−5^ s^−1^), the dramatically improved alkaline stability of **8a** may suggest that the arylthio group may also serve as a sterically demanding substituent to protect the cationic center. On the other hand, the arylthio group may not affect the stability of **8a** from the viewpoint of the electronic factors, because there is no significant difference in the positive charges of the phosphorus centers of **8a** and **2a** as estimated from the density functional theory calculations (Table S9).

In 4 M KOH/CD_3_OH, **8a** degraded completely within 7 d (Figure 5). In a series of the TAP cations (**2b**–**5b**), only 12% of **2b** survived for 8 d in 4 M KOH/CD_3_OH at 80 °C, while approximately 20, 50, and 60% of **3b**, **4b**, and **5b**, respectively, survived for 27–28 d, despite under the forced alkaline conditions as mentioned above. The alkaline stability is in the order of **2b** (*k*=3.1×10^−6^ s^−1^) <**3b** (*k*=6.4×10^−7^ s^−1^) <**4b** (*k*=5.3×10^−7^ s^−1^) <**5b** (*k*=2.3×10^−7^ s^−1^), which would correlate with the number of the methyl groups on the aromatic substituents. By comparing the degradation rate constants (*k* values), **5b** was found to be 52 times more stable than the benchmark cation (**BTMA** with a *k* value of 1.2×10^−5^ s^−1^ in 4 M KOH/CD_3_OH). These results indicate that our molecular design of introducing sterically demanding substituents on the cationic phosphorus centers is effective to improve the alkaline stability of the TAP cations.

## Conclusions

Tetraarylphosphonium (TAP) cations with highly sterically demanding aromatic substituents were designed to develop new alkaline‐stable cationic building blocks for application to anion‐exchange membranes (AEMs). Taking advantage of highly reactive arynes, the TAP salts were obtained in 88–92% yields under mild conditions from triarylphosphines with plural methyl groups on their aromatic substituents. In addition, a TAP salt carrying the *ortho* arylthio group was obtained in 81% yield from tri(*o*‐tolyl)phosphine sulfide. The degradation of the TAP cations proved to be suppressed dramatically by the plural methyl substituents as well as the arylthio group. For example, approximately 60% of bis(2,5‐dimethylphenyl)bis(2,4,6‐trimethylphenyl)phosphonium survived for 27 d under the forced alkaline conditions (i.e., in 4 M KOH/CD_3_OH at 80 °C), while the complete degradation of tetraphenylphosphonium took place within 10 min in 1 M KOH/CD_3_OH at that temperature. Through the decomposition of the alkaline‐stable TAP cations, not only triarylphosphine oxides, which are often reported to form *via* the nucleophilic attack toward the cationic phosphorus center, but also triarylphosphines were detected, which suggested the presence of other degradation mechanisms such as the nucleophilic *ipso* attack of CD_3_O^−^ ion and/or the proton abstraction from the *ortho* position due to the sterically demanding aromatic substituents. In the kinetic analyses of the degradation of the TAP cations, bis(2,5‐dimethylphenyl)bis(2,4,6‐trimethylphenyl)phosphonium was found to be the most alkaline stable among the TAP cations synthesized in the present study which exhibits 52 times higher alkaline stability compared to the benchmark cation, benzyltrimethylammonium.

On the basis of the present molecular design, a new class of highly alkaline‐stable organic cations has been established. Further molecular design of super robust cations as well as the macromolecular engineering to fabricate the TAP cations into polymers are in progress.

## Conflict of Interests

The authors declare no conflict of interest.

1

## Supporting information

As a service to our authors and readers, this journal provides supporting information supplied by the authors. Such materials are peer reviewed and may be re‐organized for online delivery, but are not copy‐edited or typeset. Technical support issues arising from supporting information (other than missing files) should be addressed to the authors.

Supporting Information

## Data Availability

The data that support the findings of this study are available in the supplementary material of this article.
