# Supplementary material for: Tetraarylphosphonium Cations with Excellent Alkaline‐Resistant Performance for Anion‐Exchange Membranes
Source: ChemSusChem. 2025 Jan 16;18(9):e202402366. doi: 10.1002/cssc.202402366 (PMC12051230; doi:10.1002/cssc.202402366)
Supplement: Supplementary file 1 — Supporting Information [file CSSC-18-e202402366-s001.pdf]

ChemSusChem

Supporting Information

Tetraarylphosphonium Cations with Excellent Alkaline-Resistant Performance for Anion-Exchange Membranes

Ryoyu Hifumi, Yoshikazu Toyama, Keisuke Ikeda, Tetsuaki Hashimoto, Tomohiro Imai, Shinsuke Inagi, and Ikuyoshi Tomita*

Supporting Information

Tetraarylphosphonium Cations with Excellent Alkaline-Resistant Performance for Anion-Exchange Membranes

Ryoyu Hifumi, Yoshikazu Toyama, Keisuke Ikeda, Tetsuaki Hashimoto, Tomohiro Imai, Shinsuke Inagi, Ikuyoshi Tomita*

Department of Chemical Science and Engineering, School of Materials and Chemical Technology, Institute of Science Tokyo, Nagatsuta-cho 4259-G1-9, Midori-ku, Yokohama 226-8501, Japan.

E-mail: tomita@cap.mac.titech.ac.jp (I. Tomita)

Contents

1. General Information

2. Materials

3. Synthetic Procedures and Characterization

    3.1 Synthesis of Phosphines and a Phosphine Sulfide

    3.2 Synthesis of Tetraarylphosphonium Salts by Reaction of Phosphines or a Phosphine Sulfide with Arynes

4. Decomposition Product Analysis

5. Relaxation Times of Tetraarylphosphonium Salts

6. Residual Amounts of Tetraarylphosphonium Cations

7. Kinetic Plots for Degradation of Tetraarylphosphonium Cations

8. Computational Results

9. References

1. General Information

All the reactions were performed under argon or nitrogen.

Nuclear magnetic resonance (^{1}H, ^{13}C, ^{19}F, and ^{31}P NMR) spectra were recorded on a JEOL ECP-300 instrument at 300, 75, 282, and 121 MHz or a JEOL ECZ-400S instrument at 400, 100, 376, and 161 MHz, respectively. Samples were analyzed in chloroform-d_1 ($CDCl_3$), benzene-d_6 (C_6D_6), methanol-d_4 (CD_3OD), or methanol-d_3 (CD_3OH), and the chemical shift values were expressed relative to tetramethylsilane as an internal or external standard for ^{1}H and ^{13}C NMR spectra, $CFCl_3$ as an external standard for ^{19}F NMR spectra, and 85% H_3PO_4 as an external standard for ^{31}P NMR spectra.

High-resolution mass spectra (HRMS) were obtained on a Bruker micrOTOF II spectrometer.

Gas chromatography/mass spectrometry (GC/MS) was performed on Agilent 7890B GC System/JEOL JMS-700 instruments. GC conditions were as follows: capillary column = HP-5 (Agilent technology), carrier = He (flow rate: 1 mL/min), split ratio = 1 : 40, injection temperature = 280 °C, oven = 50 °C, 2.5 min hold, 20 °C/min heating to 280 °C, 280 °C, 3 min hold. Mass analysis was performed in electron impact mode.

Melting points were determined in capillary tubes using a Stuart SMP10 melting point apparatus.

The density functional theory (DFT) calculations with the Becke-three-parameter-Lee–Yang–Parr hybrid (B3LYP) were performed using the Gaussian 16 (Revision C.01) program package.[S1] The 6-31g(d,p) basis set was used for the geometry optimizations and the natural bond orbital analyses.

2. Materials

Phosphorus trichloride (PCl_3) was purchased from Kanto Chemical and was used as received. Benzyltrimethylammonium chloride (**BTMA**•Cl) and 2-bromo-*m*-xylene were purchased from Tokyo Chemical Industry and were used as received. 2-Bromo-*p*-xylene was purchased from Angene Chemical and was used as received. 2-Bromo-1,3,5-trimethylbenzene, sulfur powder, and tetraphenylphosphonium bromide (**1a**•Br) were purchased from Sigma-Aldrich and were used as received. Potassium hydroxide (KOH) was purchased from FUJIFILM Wako Pure Chemical and was used as received.

Tri(*o*-tolyl)phosphine (**2**) was purchased from Wako Pure Chemical Industries and were purified by the recrystallization from ethanol. Magnesium turnings and cesium fluoride (CsF) were purchased from Nacalai Tesque and Sigma-Aldrich, respectively, and were heated with a heat gun under vacuum prior to use.

2-(Trimethylsilyl)phenyl triflate (precursor **a**) and 3,6-dimethyl-2-(trimethylsilyl)phenyl triflate (precursor **b**) were synthesized, according to the methods reported in the literature.[S2,S3]

Tetrahydrofuran (THF) and toluene were distilled over sodium prior to use. Acetonitrile (CH_3CN) was distilled over phosphorus pentoxide prior to use. 1-Methyl-2-pyrrolidone (NMP) was distilled over CaH_2 under vacuum prior to use. Other solvents (hexane, ethyl acetate, methanol, ethanol, $CHCl_3$, and CH_2Cl_2) were used as received. $CDCl_3$ and C_6D_6 were purchased from Kanto Chemical, and CD_3OH was purchased from TAIYO NIPPON SANSO. These solvents were used as received.

Silica gel (SiO_2) for column chromatography was purchased from Kanto Chemical (Silica gel 60N, spherical, neutral, particle size 63–210μm) and was used as received.

3. Synthetic Procedures and Characterization

3.1 Synthesis of Phosphines and a Phosphine Sulfide

Tris(2,5-dimethylphenyl)phosphine (3)

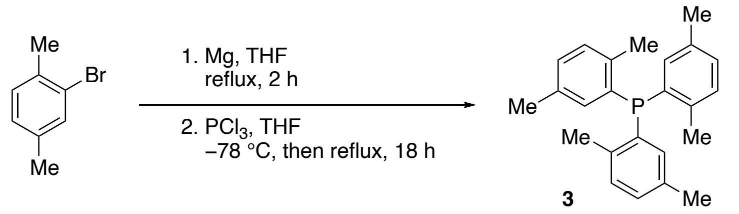

A solution of 2-bromo-*p*-xylene (4.44 g, 24.0 mmol, 4.00 eq.) in THF (24 mL) was slowly added to magnesium turnings (0.583 g, 24.0 mmol, 4.00 eq.) at ambient temperature. The resulting mixture was stirred under reflux for 2 h. After cooling to ambient temperature, the prepared Grignard reagent was slowly added to a solution of PCl_3 (0.824 g, 6.00 mmol, 1 eq.) in THF (15 mL) at −78 °C. The reaction mixture was stirred for 15 min at ambient temperature and for 18 h under reflux. After adding saturated NH_4Cl aq. to the reaction mixture to quench the reaction, the mixture was extracted with ethyl acetate and the organic layer was dried over $MgSO_4$. After the removal of the volatile fractions by evaporation, the residue was purified by column chromatography (SiO_2, hexane as an eluent) and recrystallization from $CHCl_3$/ethanol to give **3** as colorless crystals (1.87 g, 5.40 mmol, 90.0% yield).

^{1}H NMR (400 MHz, $CDCl_3$) δ 2.16 (s, 9H), 2.32 (s, 9H), 6.53 (s, 3H), 7.03–7.12 (6H) ppm.

^{13}C{^{1}H} NMR (100 MHz, $CDCl_3$) δ 20.7 (d, $^3J_{C–P}$ = 21.2 Hz), 21.1, 129.3, 129.8 (d, $J_{C–P}$ = 5.3 Hz), 133.4, 134.2 (d, $J_{C–P}$ = 10.6 Hz), 135.2, 139.5 (d, $J_{C–P}$ = 26.0 Hz) ppm.

^{31}P{^{1}H} NMR (161 MHz, $CDCl_3$) δ −27.8 (s) ppm.

HRMS (ESI) *m/z*: $[M + H]^+$ calcd for $C_{24}H_{28}P$, 347.1923; found, 347.1922.

The data are consistent with the literature.[S4]

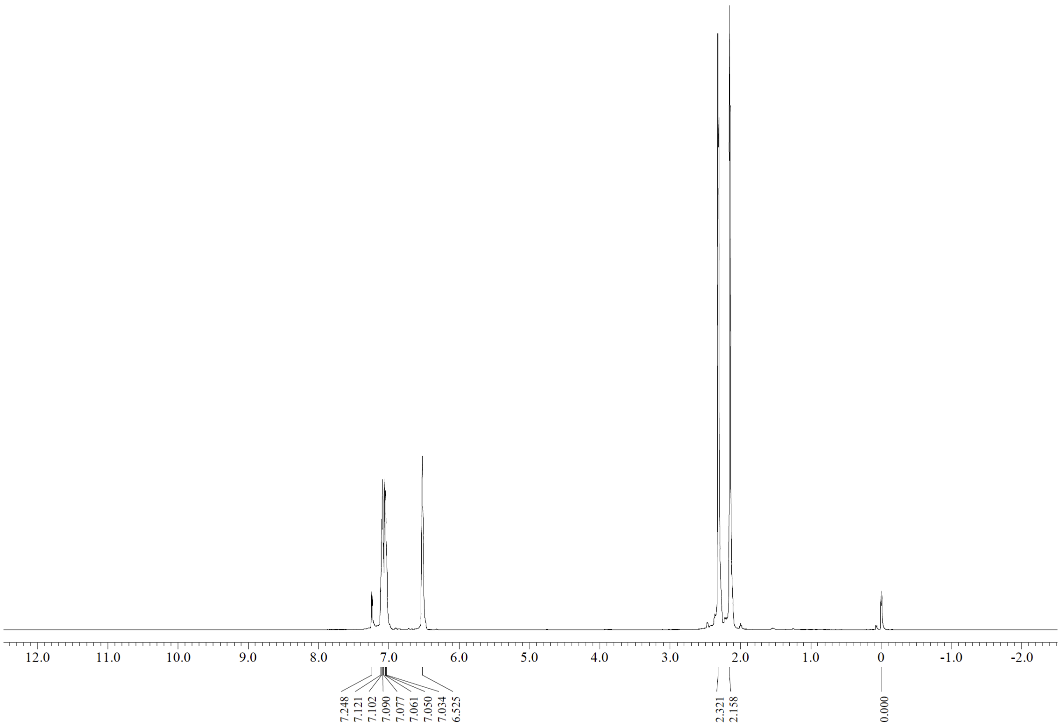

Figure S1. ^{1}H NMR spectrum of **3** in $CDCl_3$.

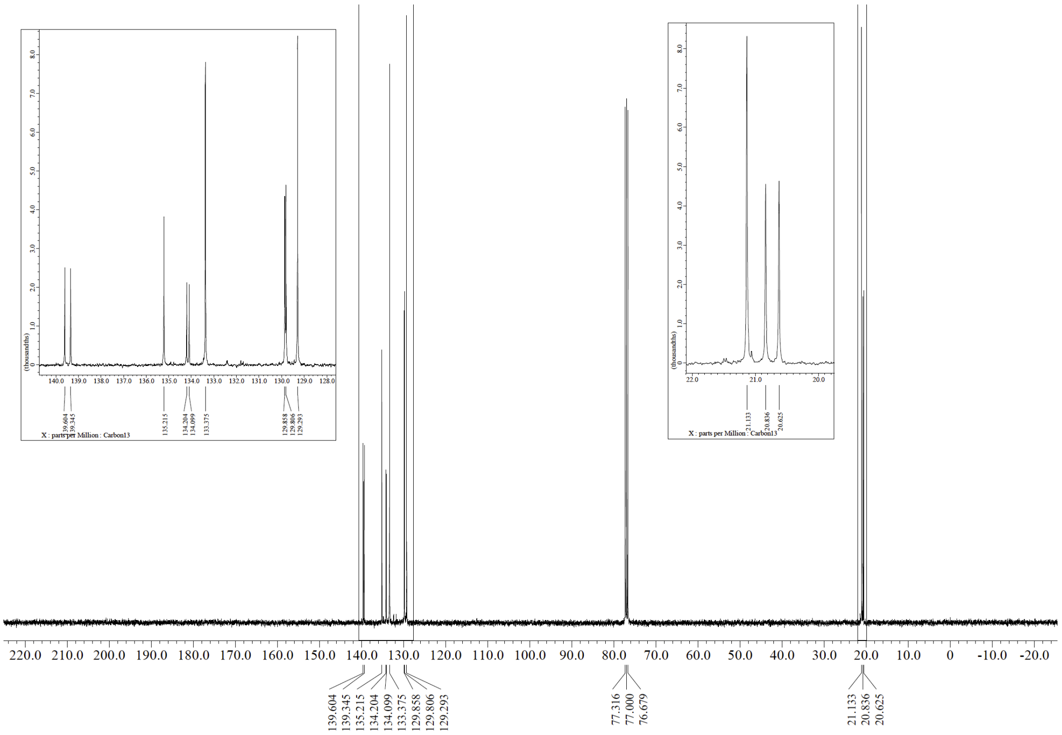

Figure S2. ^{13}C NMR spectrum of **3** in $CDCl_3$.

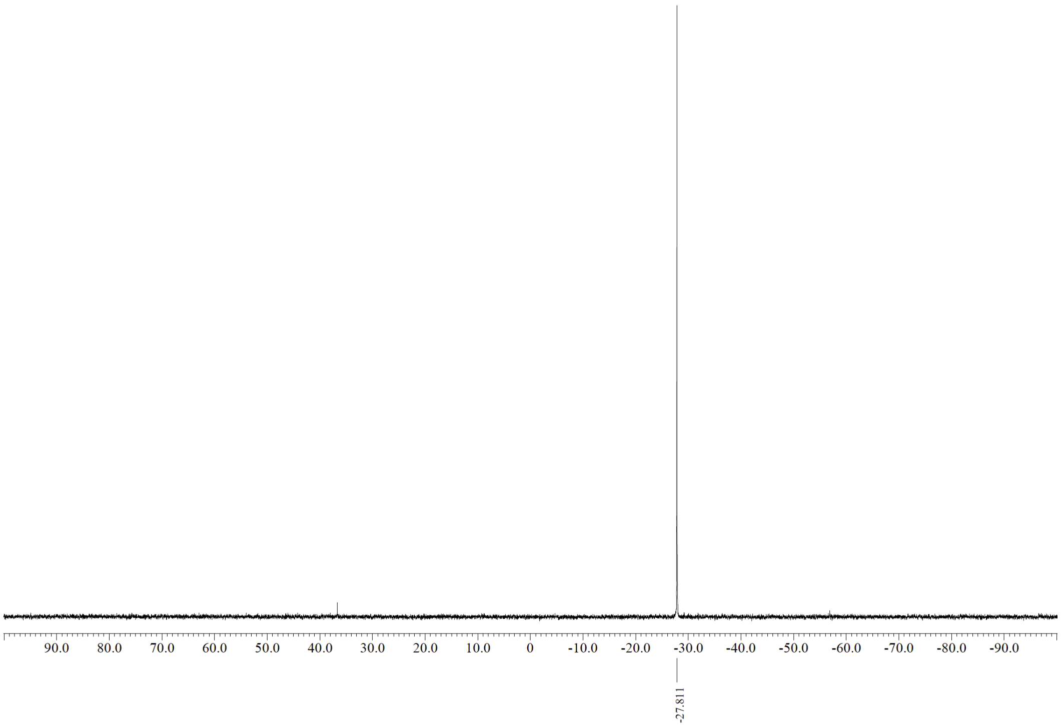

Figure S3. ^{31}P NMR spectrum of **3** in $CDCl_3$.

Bis(2,5-dimethylphenyl)(2,4,6-trimethylphenyl)phosphine (4)

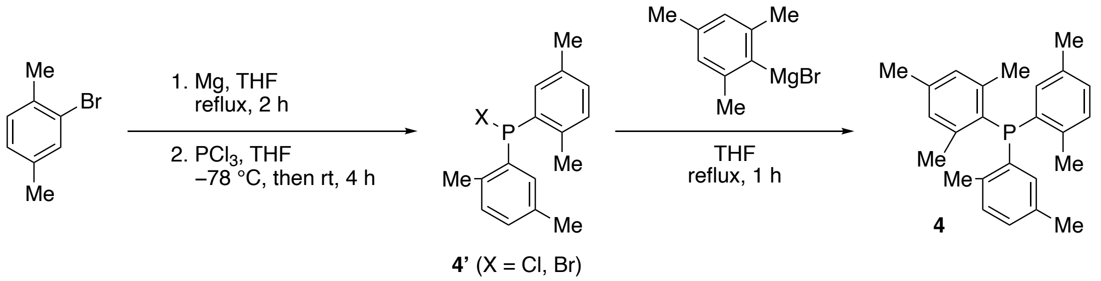

A solution of 2-bromo-*p*-xylene (2.26 g, 12.2 mmol, 2.04 eq.) in THF (12 mL) was slowly added to magnesium turnings (0.297 g, 12.2 mmol, 2.04 eq.) at ambient temperature. The resulting mixture was stirred under reflux for 2 h. After cooling to ambient temperature, the prepared Grignard reagent was slowly added to a solution of PCl_3 (0.824 g, 6.00 mmol, 1 eq.) in THF (15 mL) at −78 °C. The reaction mixture was allowed to warm to ambient

temperature and stirred for 4 h. After confirming the selective formation of **4’** by ^{31}P NMR spectroscopy in dry C_6D_6 (Figure S4), a solution of 2,4,6-trimethylphenylmagnesium bromide likewise prepared from magnesium turnings (0.160 g, 6.58 mmol, 1.10 eq.), 2-bromo-1,3,5-trimethylbenzene (1.31 g, 6.58 mmol, 1.10 eq.), and THF (6 mL) was added to the mixture containing **4’** at ambient temperature and the resulting reaction mixture was stirred for 1 h under reflux. After adding saturated NH_4Cl aq. to the reaction mixture to quench the reaction, the mixture was extracted with ethyl acetate and the organic layer was dried over $MgSO_4$. After the removal of the volatile fractions by evaporation, the residue was purified by column chromatography (SiO_2, hexane as an eluent) and recrystallization from $CHCl_3$/ethanol to give **4** as colorless crystals (1.05 g, 2.91 mmol, 48.5% yield).

Melting point: 138–140 °C.

^{1}H NMR (400 MHz, $CDCl_3$) δ 2.15 (s, 6H), 2.17 (s, 6H), 2.21 (s, 6H), 2.29 (s, 3H), 6.78 (d, $J_{H\text{–}P}$ = 3.8 Hz, 2H), 6.86 (d, $J_{H\text{–}P}$ = 2.2 Hz, 2H), 7.00–7.09 (4H) ppm.

^{13}C{^{1}H} NMR (100 MHz, $CDCl_3$) δ 20.7 (d, $^3J_{C\text{–}P}$ = 19.3 Hz), 21.1, 21.2, 23.5 (d, $^3J_{C\text{–}P}$ = 17.8 Hz), 127.0 (d, $J_{C\text{–}P}$ = 12.5 Hz), 128.6, 129.8 (d, $J_{C\text{–}P}$ = 4.3 Hz), 129.9 (d, $J_{C\text{–}P}$ = 4.3 Hz), 132.3 (d, $J_{C\text{–}P}$ = 1.9 Hz), 134.0 (d, $J_{C\text{–}P}$ = 13.0 Hz), 134.8, 138.7 (d, $J_{C\text{–}P}$ = 26.0 Hz), 139.3 (d, $J_{C\text{–}P}$ = 1.0 Hz), 145.2 (d, $J_{C\text{–}P}$ = 15.9 Hz) ppm.

^{31}P{^{1}H} NMR (161 MHz, $CDCl_3$) δ −28.1 (s) ppm.

HRMS (ESI) *m/z*: $[M + H]^+$ calcd for $C_{25}H_{30}P$, 361.2080; found, 361.2084.

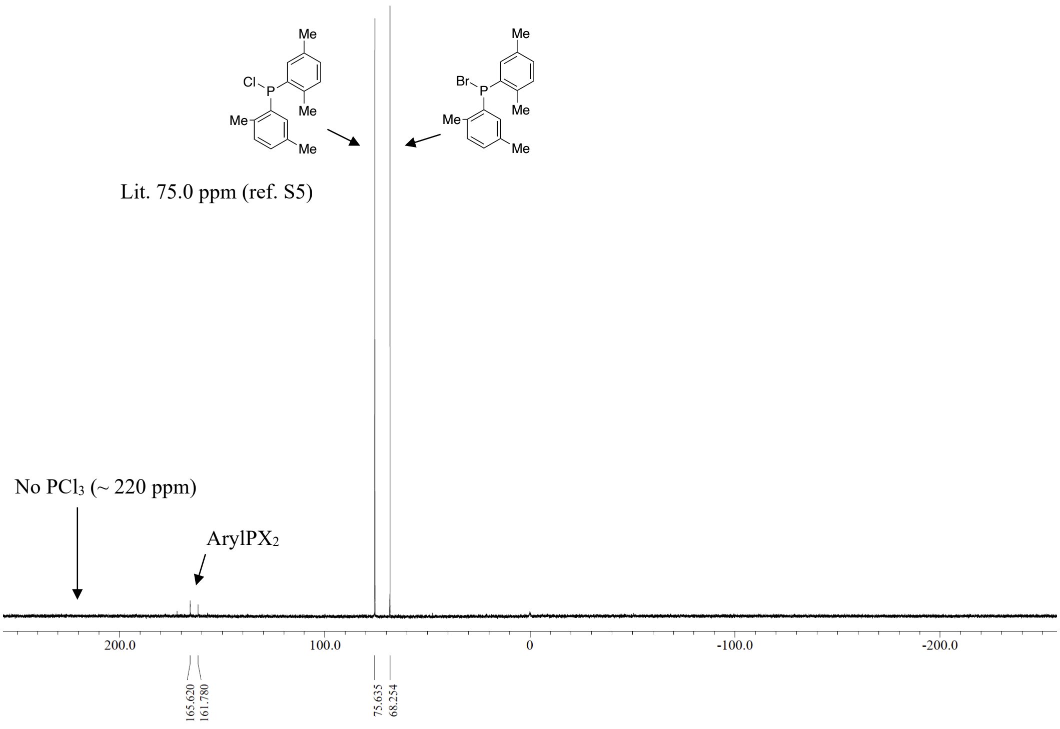

Figure S4. ^{31}P NMR spectrum of **4'** in dry C_6D_6.

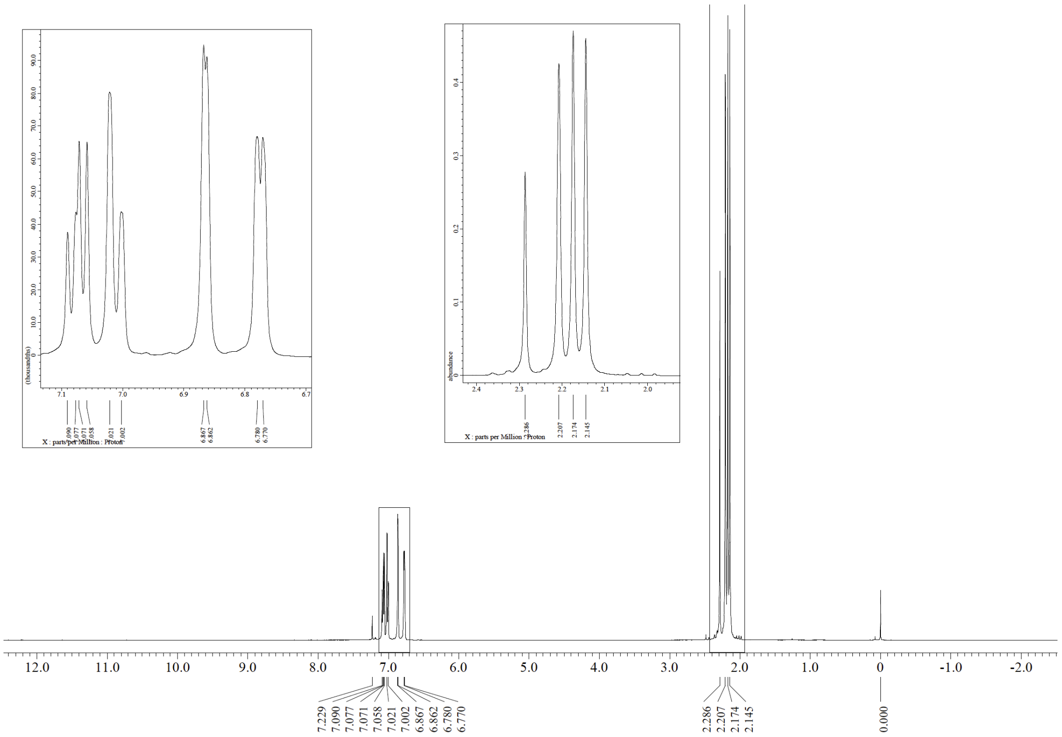

Figure S5. ^{1}H NMR spectrum of **4** in $CDCl_3$.

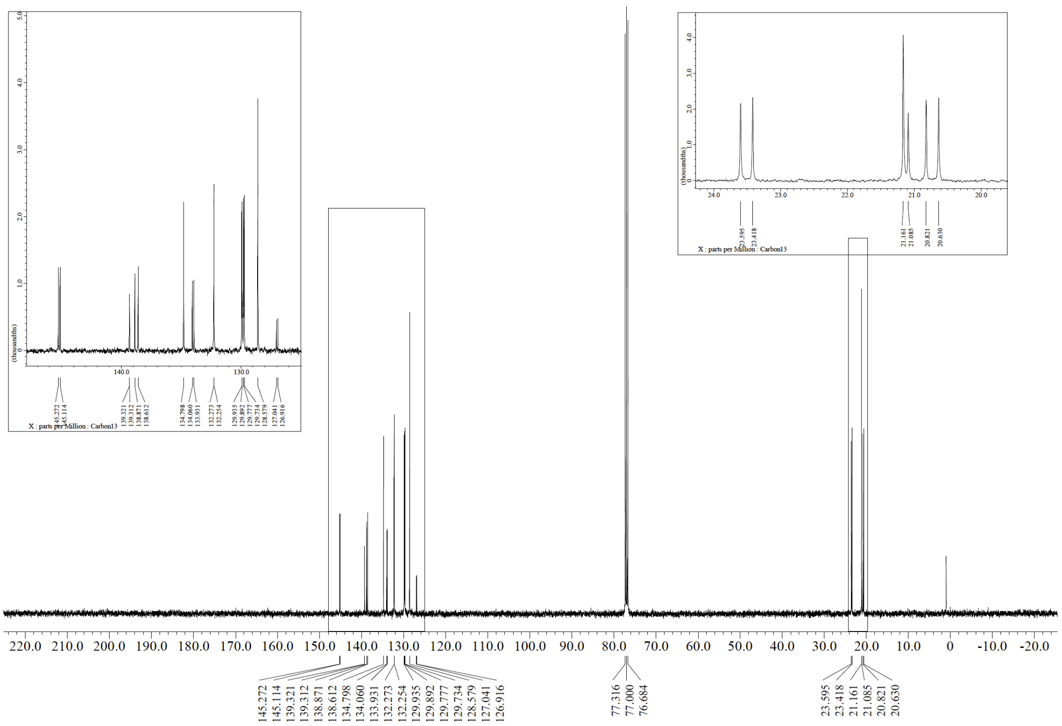

Figure S6. ^{13}C NMR spectrum of **4** in $CDCl_3$.

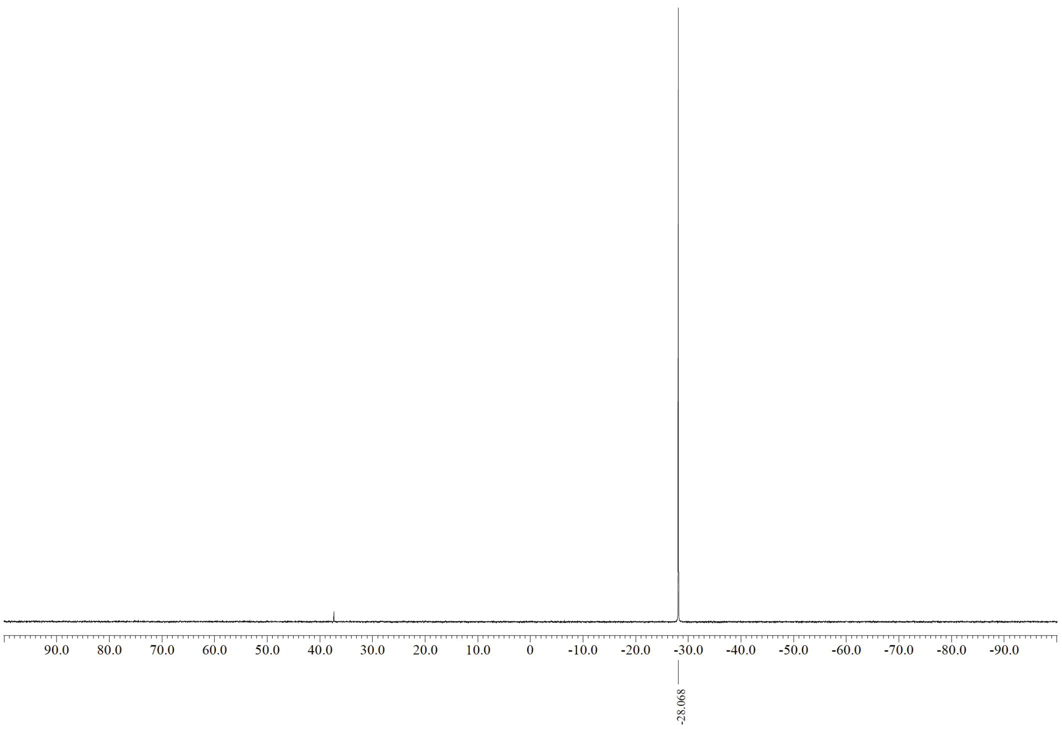

Figure S7. ^{31}P NMR spectrum of **4** in $CDCl_3$.

(2,5-Dimethylphenyl)bis(2,4,6-trimethylphenyl)phosphine (5)

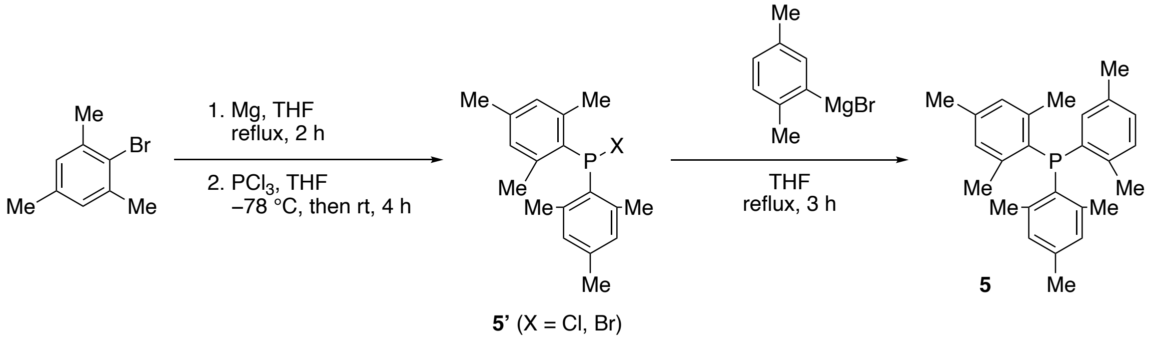

According to the procedure for the phosphine (**4**), (2,5-dimethylphenyl)bis(2,4,6-trimethylphenyl)phosphine (**5**) was likewise prepared from PCl_3 (1.65 g, 12.0 mmol, 1 eq.) in THF (30 mL), 2,4,6-trimethylphenylmagnesium bromide (prepared from magnesium turnings (0.592 g, 24.4 mmol, 2.03 eq.) and 2-bromo-1,3,5-trimethylbenzene (4.85 g, 24.4 mmol, 2.03 eq.) in THF (25 mL)), and 2,5-dimethylphenylmagnesium bromide (prepared from magnesium turnings (0.321 g, 13.2 mmol, 1.10 eq.) and 2-bromo-*p*-xylene (2.44 g, 13.2 mmol, 1.10 eq.) in THF (15 mL)). **5** was obtained as colorless crystals (1.41 g, 3.76 mmol, 31.3% yield).

^{1}H NMR (400 MHz, $CDCl_3$) δ 2.09 (s, 12H), 2.14 (s, 3H), 2.25 (s, 6H), 2.31 (s, 3H), 6.78–6.81 (5H), 6.98 (d, $J_{H–P}$ = 7.8 Hz, 1H), 7.05 (dd, J = 5.7 Hz, J = 7.4 Hz, 1H) ppm.

^{13}C{^{1}H} NMR (100 MHz, $CDCl_3$) δ 20.7 (d, $^3J_{C–P}$ = 23.6 Hz), 20.9, 21.2, 22.6 (d, $^3J_{C–P}$ = 16.4 Hz), 128.7, 129.5 (d, $J_{C–P}$ = 17.8 Hz), 129.8 (d, $J_{C–P}$ = 5.3 Hz), 129.8 (d, $J_{C–P}$ = 3.4 Hz), 132.2, 134.8, 136.0 (d, $J_{C–P}$ = 13.0 Hz), 137.9, 139.5 (d, $J_{C–P}$ = 29.9 Hz), 143.3 (d, $J_{C–P}$ = 15.9 Hz) ppm.

^{31}P{^{1}H} NMR (161 MHz, $CDCl_3$) δ −30.5 (s) ppm.

HRMS (ESI) *m/z*: $[M + H]^+$ calcd for $C_{26}H_{32}P$, 375.2236; found, 375.2231.

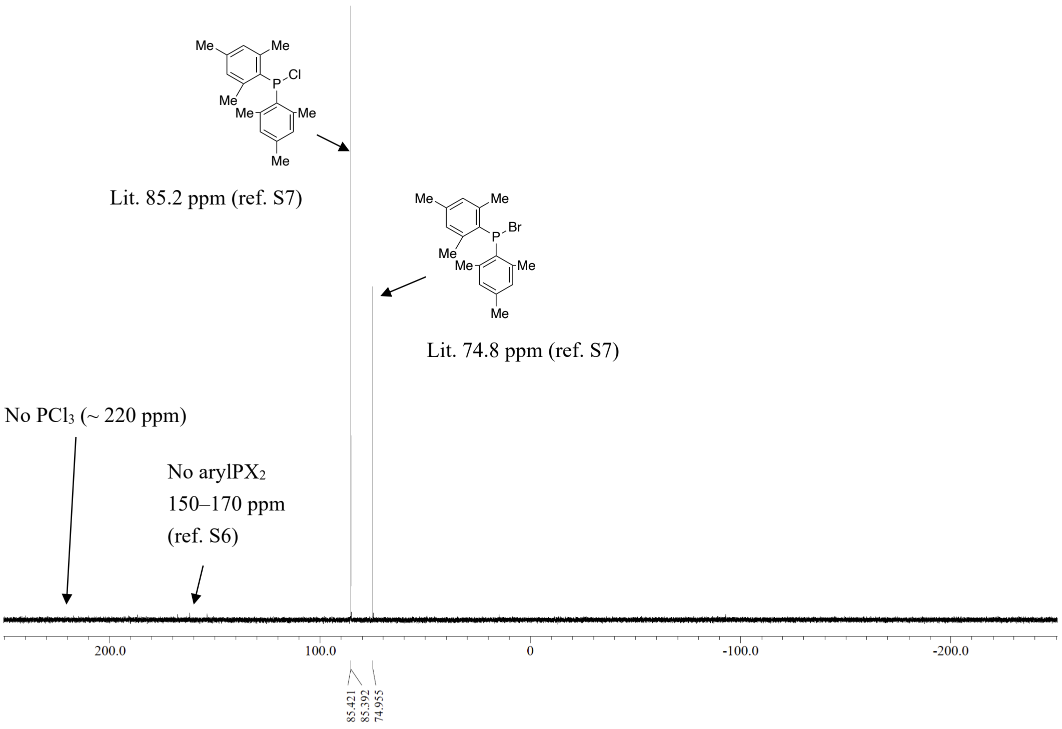

Figure S8. ^{31}P NMR spectrum of **5'** in dry C_6D_6.

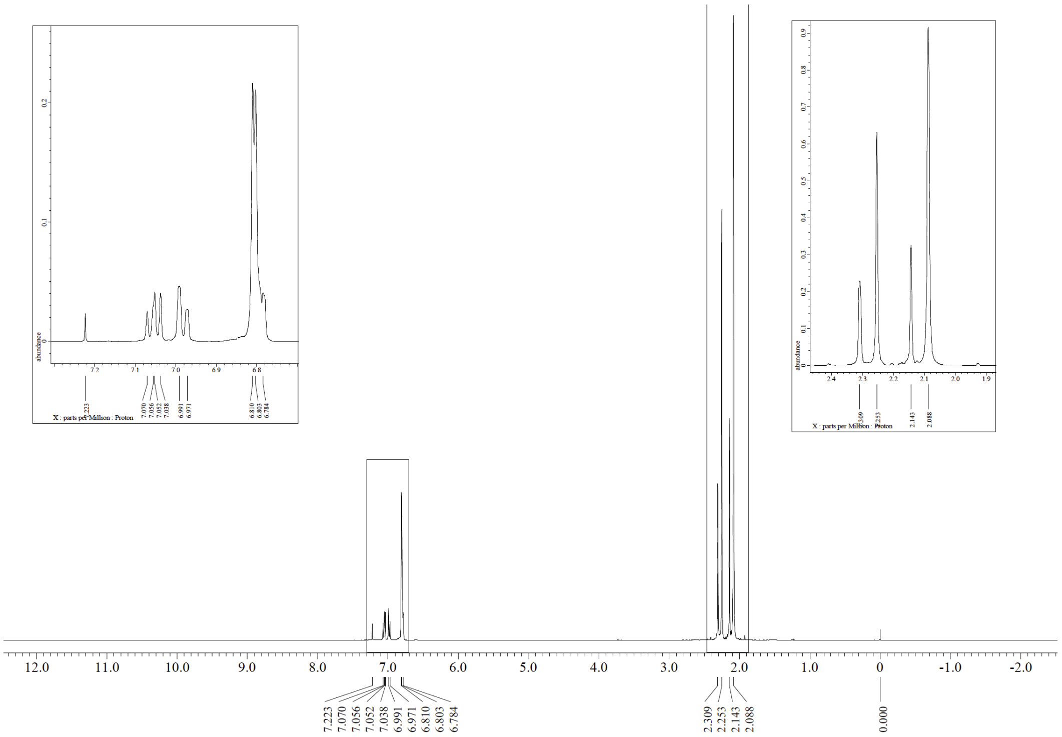

Figure S9. ^{1}H NMR spectrum of **5** in $CDCl_3$.

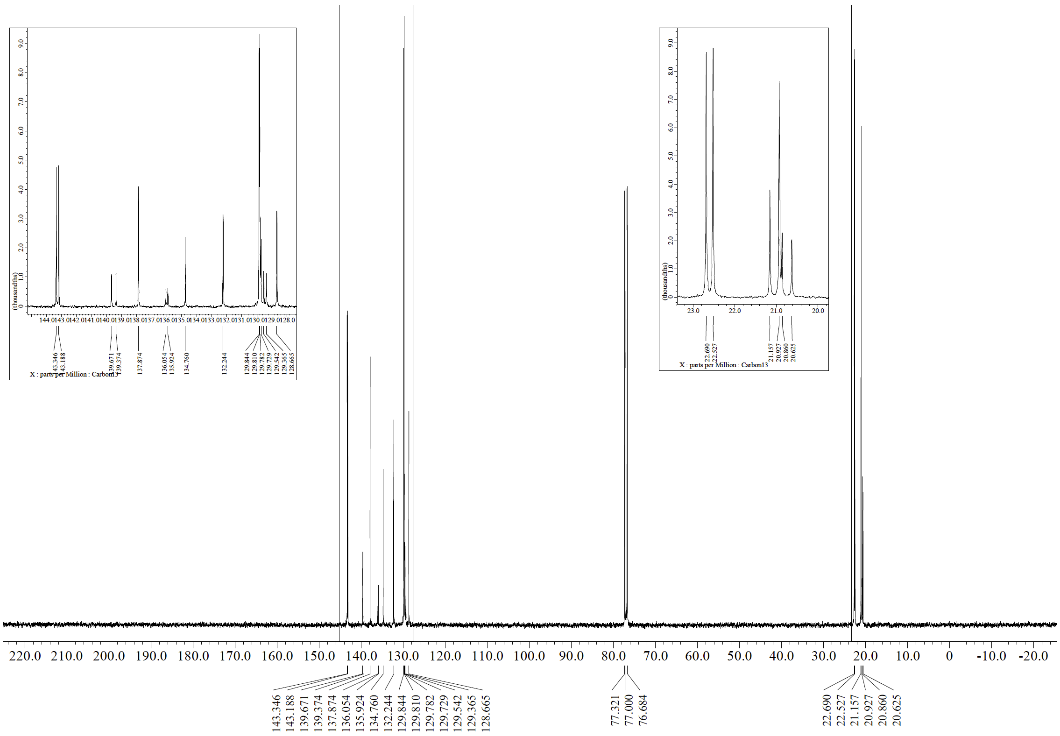

Figure S10. ^{13}C NMR spectrum of **5** in $CDCl_3$.

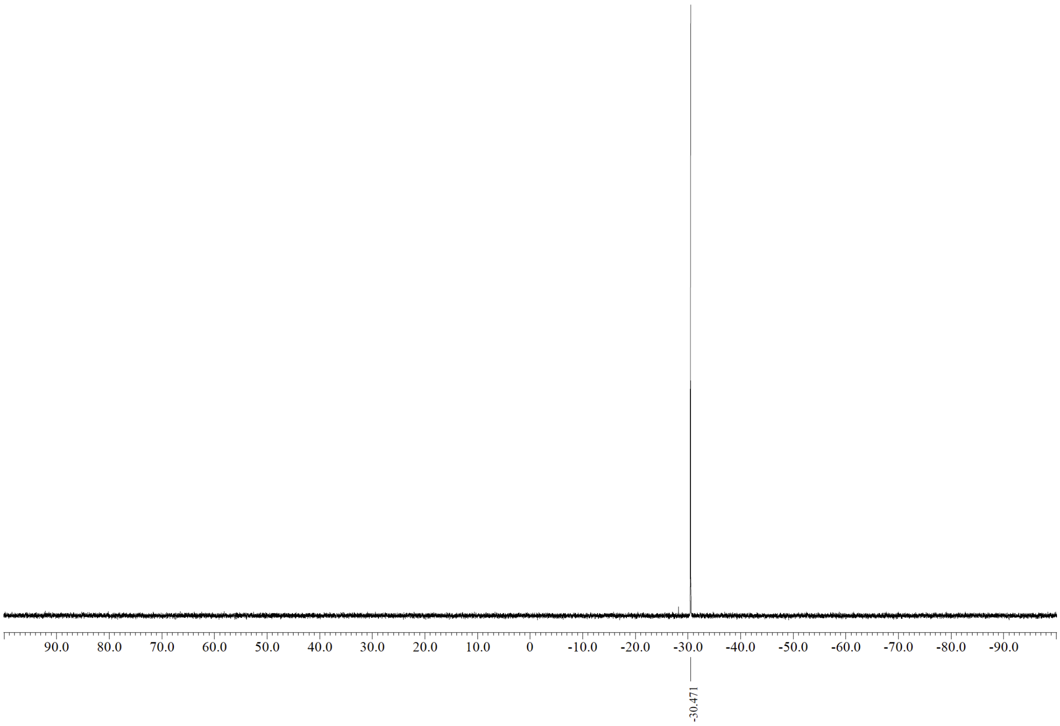

Figure S11. ^{31}P NMR spectrum of **5** in $CDCl_3$.

Tris(2,6-dimethylphenyl)phosphine (6)

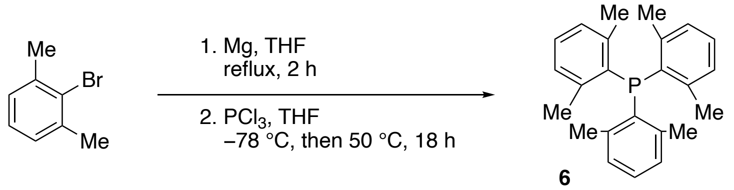

According to the procedure for the phosphine (**3**), tris(2,6-dimethylphenyl)phosphine (**6**) was likewise prepared from PCl_3 (0.837 g, 6.10 mmol, 1 eq.) in THF (15 mL) and 2,6-dimethylphenylmagnesium bromide (prepared from magnesium turnings (0.593 g, 24.4 mmol, 4.00 eq.) and 2-bromo-*m*-xylene (4.52 g, 24.4 mmol, 4.01 eq.) in THF (24 mL)). **6** was obtained as colorless crystals (0.924 g, 2.67 mmol, 43.8% yield).

^{1}H NMR (300 MHz, $CDCl_3$) δ 2.08 (s, 18H), 6.96 (dd, $^{4}J_{H\text{–}P}$ = 3.3 Hz, $^{3}J_{H\text{–}H}$ = 7.5 Hz, 6H), 7.11 (t, $^{3}J_{H\text{–}H}$ = 7.4 Hz, 3H) ppm.

^{13}C{^{1}H} NMR (100 MHz, $CDCl_3$) δ 22.9 (d, $^{3}J_{C\text{–}P}$ = 16.9 Hz), 128.0, 128.8, 134.7 (d, $J_{C\text{–}P}$ = 18.8 Hz), 142.7 (d, $J_{C\text{–}P}$ = 17.8 Hz) ppm.

^{31}P{^{1}H} NMR (161 MHz, $CDCl_3$) δ −33.9 (s) ppm.

HRMS (ESI) *m/z*: $[M + H]^+$ calcd for $C_{24}H_{28}P$, 347.1923; found, 347.1930.

The data are consistent with the literature.[S4]

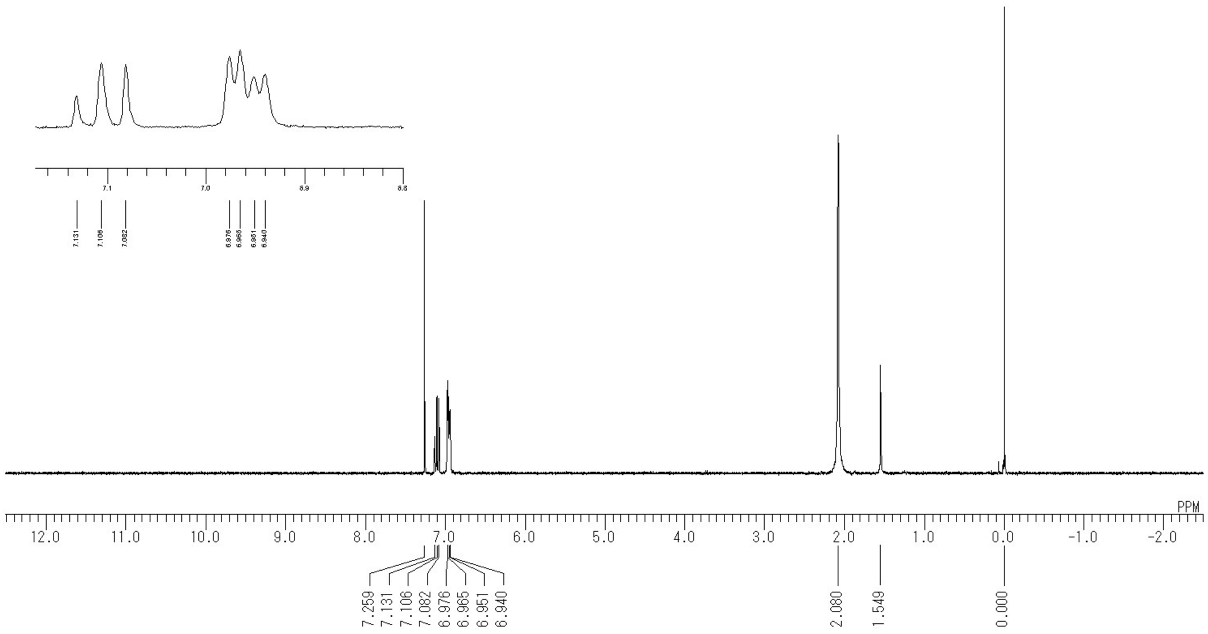

Figure S12. ^{1}H NMR spectrum of **6** in $CDCl_3$.

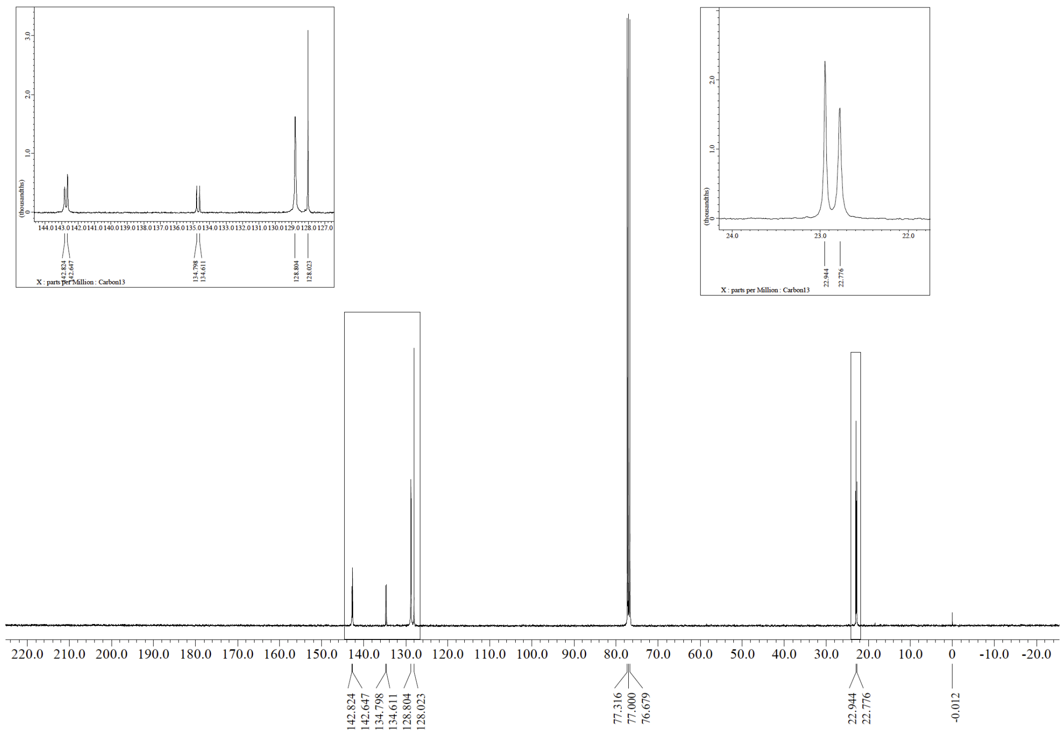

Figure S13. ^{13}C NMR spectrum of **6** in $CDCl_3$.

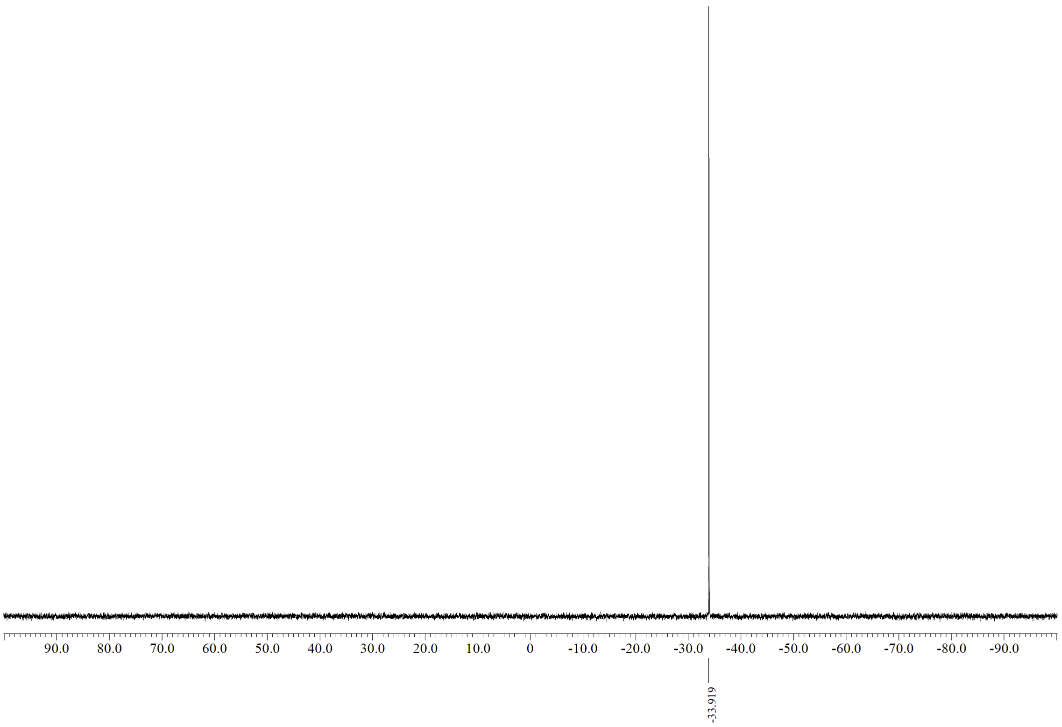

Figure S14. ^{31}P NMR spectrum of **6** in $CDCl_3$.

Tris(2,4,6-trimethylphenyl)phosphine (7)

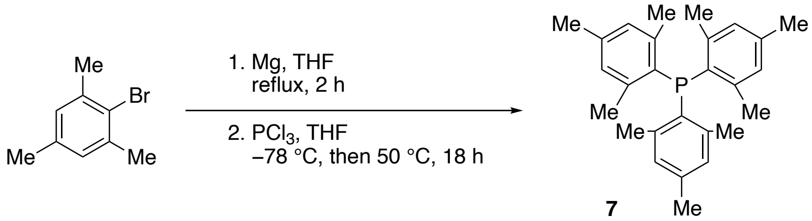

According to the procedure for the phosphine (**3**), tris(2,4,6-trimethylphenyl)phosphine (**7**) was likewise prepared from PCl_3 (0.822 g, 5.99 mmol, 1 eq.) in THF (15 mL) and 2,4,6-trimethylphenylmagnesium bromide (prepared from magnesium turnings (0.583 g, 24.0 mmol, 4.01 eq.) and 2-bromo-1,3,5-trimethylbenzene (4.78 g, 24.0 mmol, 4.01 eq.) in THF (24 mL)). **7** was obtained as colorless crystals (1.12 g, 2.88 mmol, 48.2% yield).

^{1}H NMR (400 MHz, $CDCl_3$) δ 2.04 (s, 18H), 2.25 (s, 9H), 6.77 (d, $^{4}J_{H\text{–}P}$ = 3.1 Hz, 6H) ppm.

$^{13}C\{^1H\}$ NMR (100 MHz, $CDCl_3$) δ 20.9, 22.7 (d, $^3J_{C–P}$ = 16.4 Hz), 129.6, 131.5 (d, $^1J_{C–P}$ = 18.3 Hz), 137.5, 142.6 (d, $^2J_{C–P}$ = 16.9 Hz) ppm.

$^{31}P\{^1H\}$ NMR (161 MHz, $CDCl_3$) δ −35.9 (s) ppm.

HRMS (ESI) *m/z*: $[M + H]^+$ calcd for $C_{27}H_{34}P$, 389.2393; found, 389.2393.

The data are consistent with the literature.[S8]

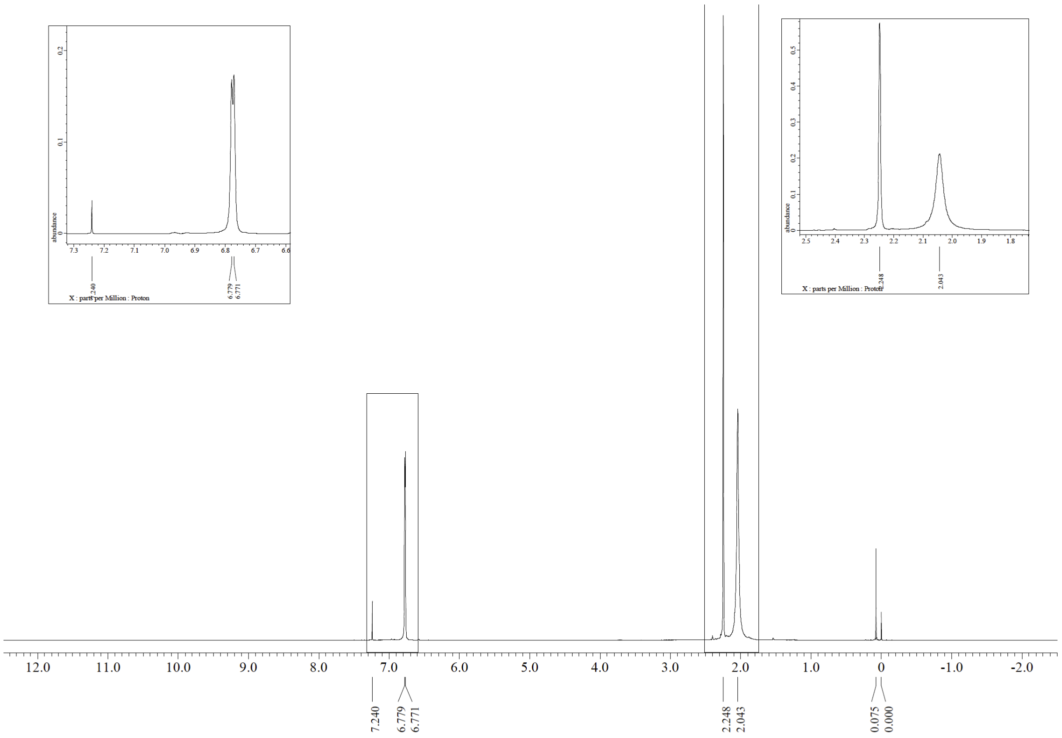

Figure S15. ^1H NMR spectrum of **7** in $CDCl_3$.

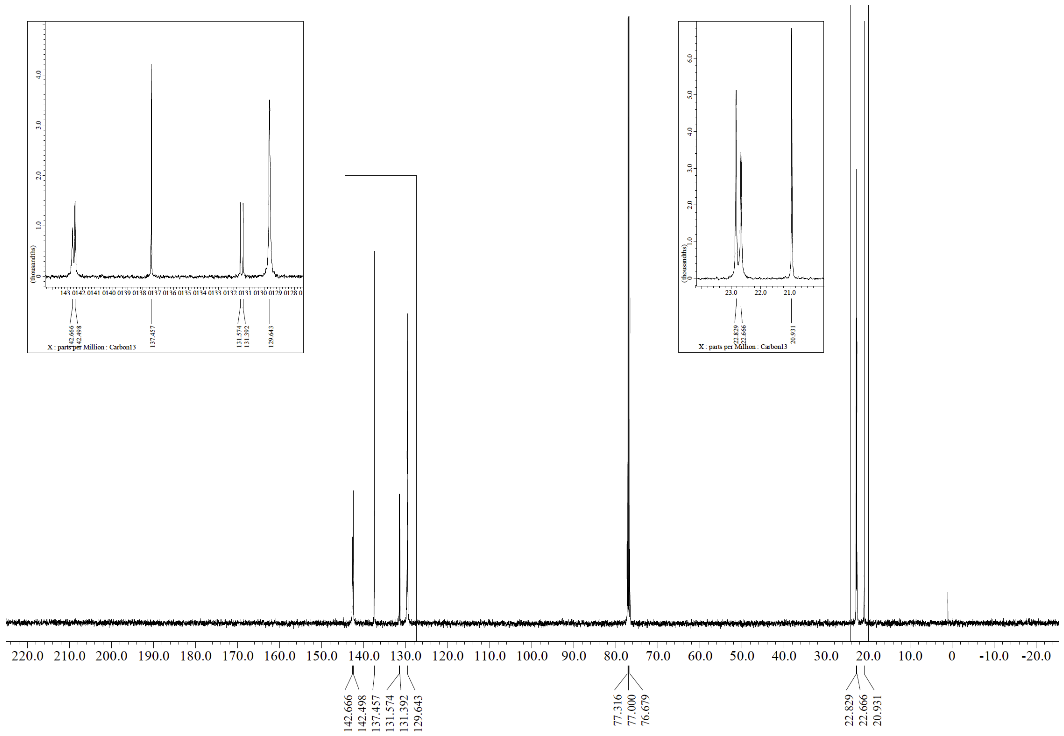

Figure S16. ^{13}C NMR spectrum of **7** in $CDCl_3$.

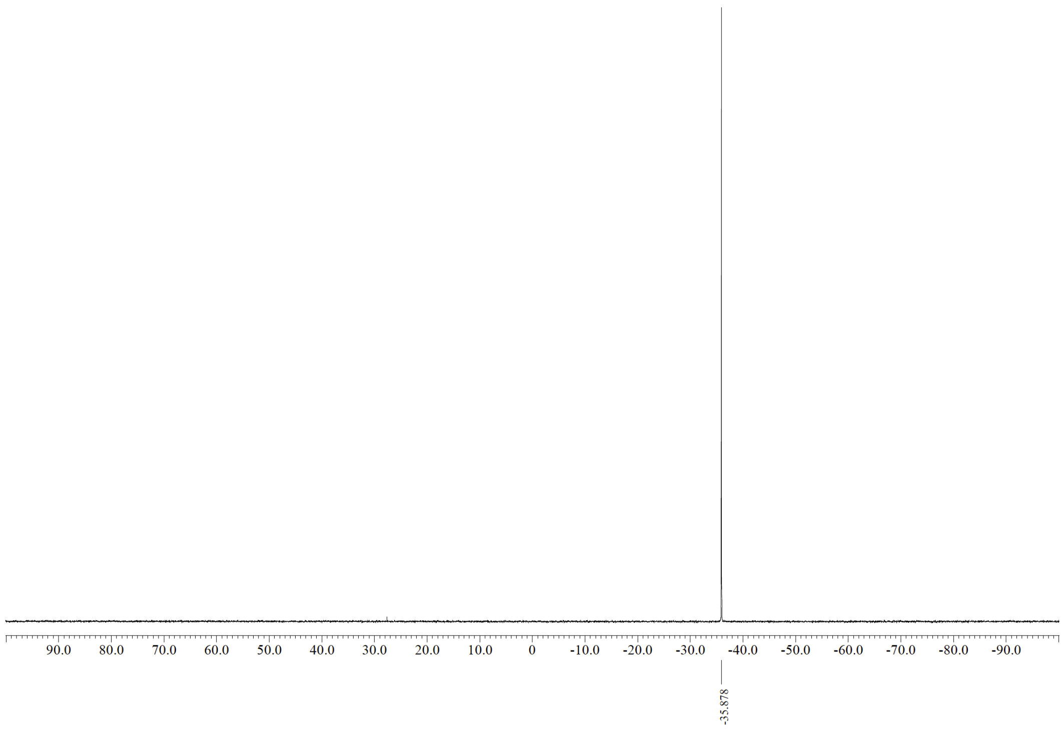

Figure S17. ^{31}P NMR spectrum of **7** in $CDCl_3$.

Tri(*o*-tolyl)phosphine sulfide (8)

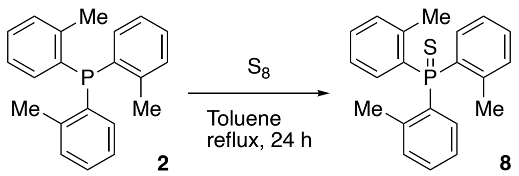

A mixture containing tri(*o*-tolyl)phosphine (**2**, 0.761 g, 2.50 mmol, 1 eq.) and sulfur powder (0.085 g, 0.33 mmol, 0.13 eq.) in toluene (25 mL) was stirred under reflux for 15 h. After the removal of the volatile fractions by evaporation, the residue was purified by column chromatography (SiO_2, hexane/CH_2Cl_2 = 2/1 vol/vol as an eluent) to give tri(*o*-tolyl)phosphine sulfide (**8**) as a white powder (0.696 g, 2.07 mmol, 82.7% yield).

^{1}H NMR (400 MHz, $CDCl_3$) δ 2.37 (s, 9H), 7.21–7.29 (6H), 7.40–7.45 (m, 3H), 7.60 (dd, $^{3}J_{H–H}$ = 7.7 Hz, $^{3}J_{H–P}$ =15.5 Hz, 3H) ppm.

^{13}C{^{1}H} NMR (100 MHz, $CDCl_3$) δ 22.8 (d, $^{3}J_{C–P}$ = 4.3 Hz), 125.9 (d, $^{3}J_{C–P}$ = 12.5 Hz), 129.4 (d, $^{1}J_{C–P}$ = 81.4 Hz), 131.7 (d, $^{4}J_{C–P}$ = 2.4 Hz), 132.6 (d, $^{2}J_{C–P}$ = 10.6 Hz), 133.3 (d, $^{3}J_{C–P}$ = 13.0 Hz), 142.7 (d, $^{2}J_{C–P}$ = 8.7 Hz) ppm.

^{31}P{^{1}H} NMR (121 MHz, $CDCl_3$) δ 42.8 (s) ppm.

HRMS (ESI) *m/z*: $[M + Na]^+$ calcd for $C_{21}H_{21}NaPS$, 359.0994; found, 359.0988.

The data are consistent with the literature.[S9]

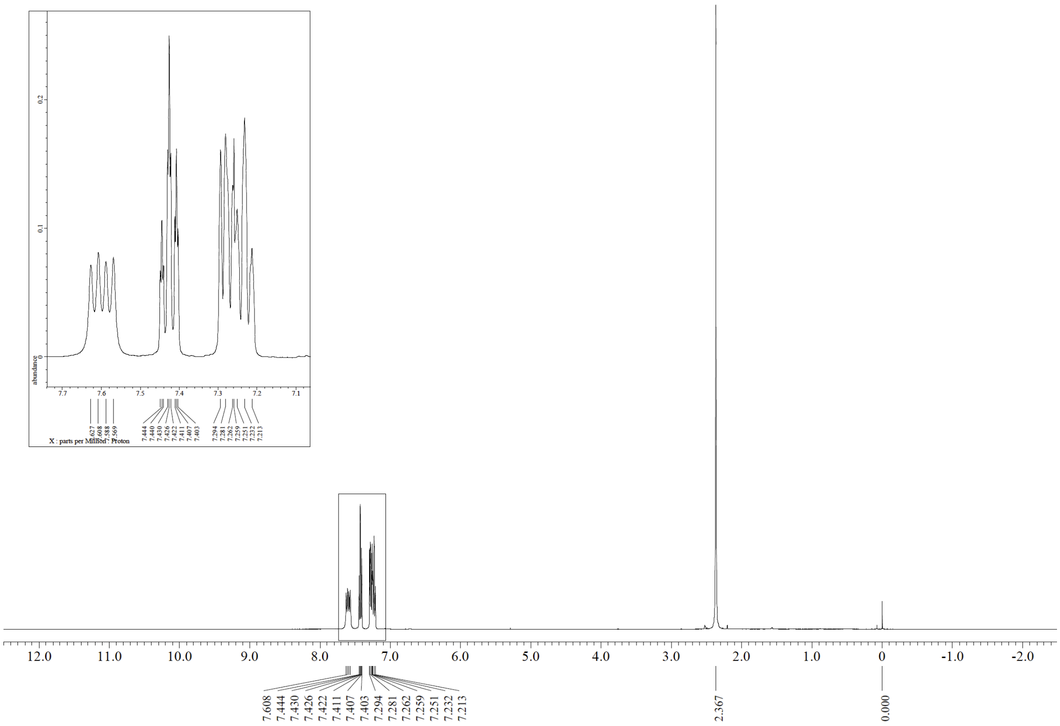

Figure S18. ^{1}H NMR spectrum of **8** in $CDCl_3$.

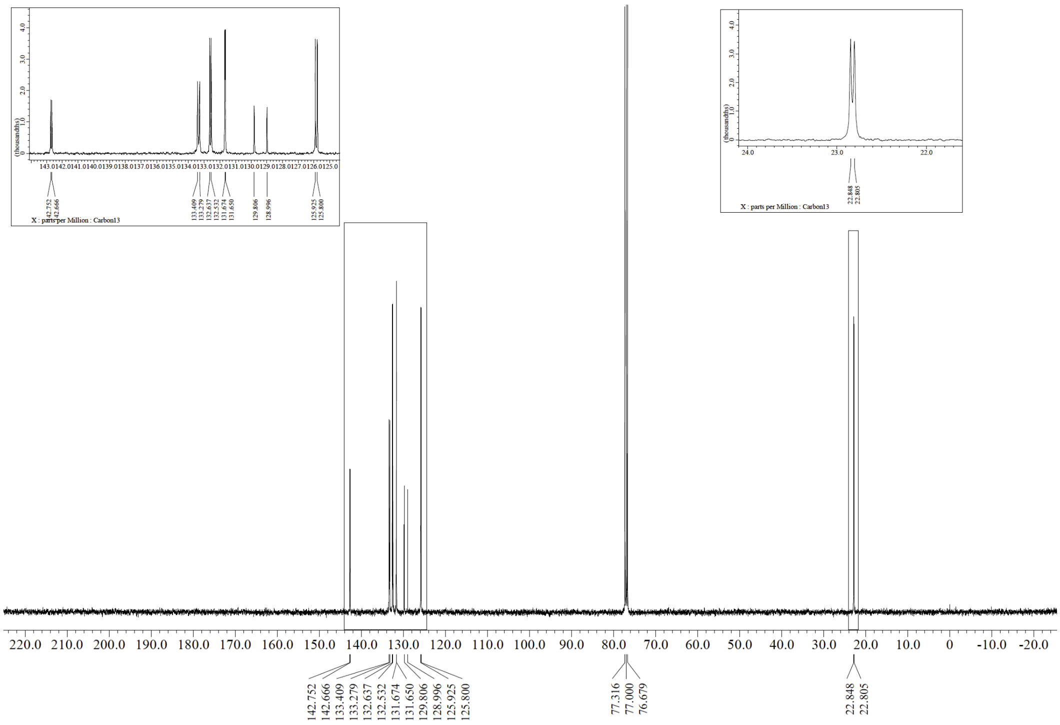

Figure S19. ^{13}C NMR spectrum of **8** in $CDCl_3$.

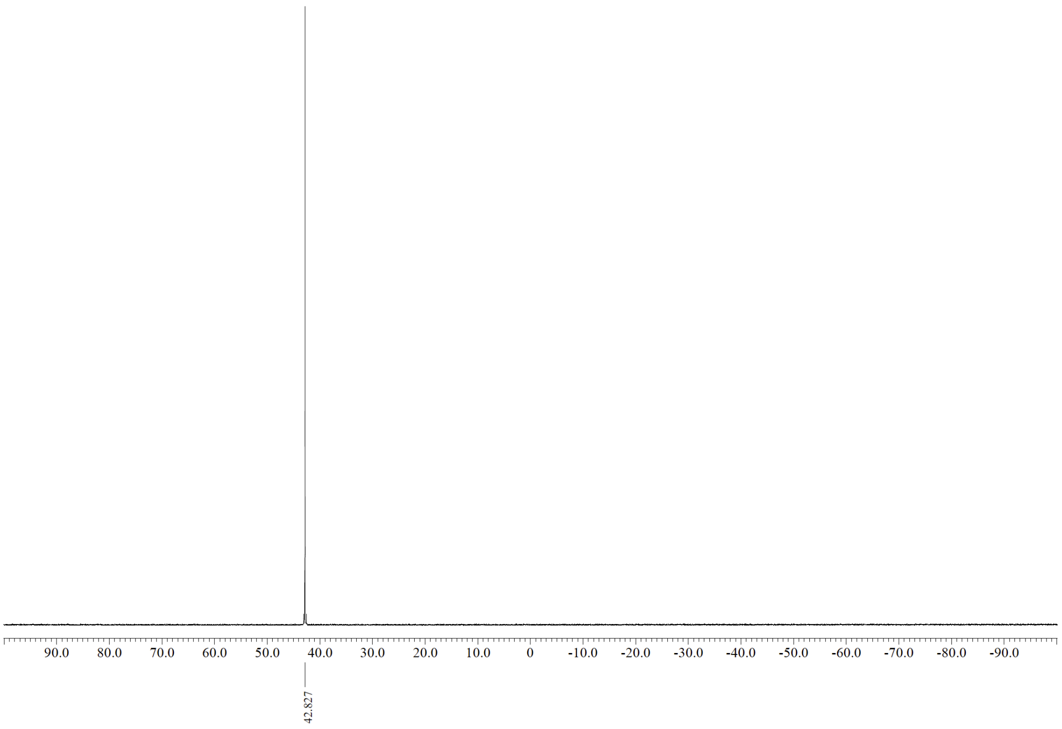

Figure S20. ^{31}P NMR spectrum of **8** in $CDCl_3$.

3.2 Synthesis of Tetraarylphosphonium Salts by Reaction of Phosphines or a Phosphine Sulfide with Arynes

(Phenyl)tri(*o*-tolyl)phosphonium triflate (2a•OTf)

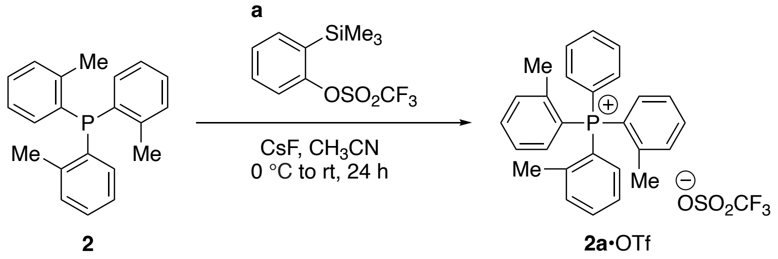

(Phenyl)tri(*o*-tolyl)phosphonium triflate (**2a**•OTf) was synthesized according to the method reported in the literature.[S10]

2-(Trimethylsilyl)phenyl triflate (**a**, 0.224 g, 0.751 mmol, 2.5 eq.) was slowly added to a mixture containing tri(*o*-tolyl)phosphine (**2**, 0.092 g, 0.30 mmol, 1 eq.) and CsF (0.273 g,

1.80 mmol, 5.9 eq.) in anhydrous CH_3CN (3 mL) at 0 °C. The reaction mixture was allowed to warm to ambient temperature and stirred for 24 h. After adding deionized water to the reaction mixture, the mixture was extracted with ethyl acetate and the organic layer was dried over $MgSO_4$. After the removal of the volatile fractions by evaporation, the residue was purified by column chromatography (SiO_2, CH_2Cl_2 to CH_2Cl_2/methanol = 20/1 vol/vol as an eluent) to give **2a**•OTf as a pale orange powder (0.111 g, 0.209 mmol, 69% yield).

^{1}H NMR (400 MHz, $CDCl_3$) δ 1.95 (s, 9H), 7.54–7.61 (11H), 7.75–7.82 (5H), 7.87–7.93 (1H) ppm.

^{13}C{^{1}H} NMR (100 MHz, $CDCl_3$) δ 22.8 (d, $^{3}J_{C\text{–}P}$ = 4.3 Hz), 115.8 (d, $^{1}J_{C\text{–}P}$ = 86.7 Hz), 117.8 (d, $^{1}J_{C\text{–}P}$ = 87.7 Hz), 120.8 (q, $^{1}J_{C\text{–}F}$ = 320.9 Hz, $\mathit{C}F_3SO_3^-$), 128.2 (d, $J_{C\text{–}P}$ = 13.5 Hz), 130.8 (d, $J_{C\text{–}P}$ = 13.0 Hz), 134.2 (d, $J_{C\text{–}P}$ = 11.1 Hz), 134.3 (d, $J_{C\text{–}P}$ = 10.6 Hz), 135.0 (d, $J_{C\text{–}P}$ = 12.5 Hz), 135.1 (d, $J_{C\text{–}P}$ = 2.9 Hz), 135.7 (d, $J_{C\text{–}P}$ = 2.9 Hz), 143.6 (d, $J_{C\text{–}P}$ = 8.7 Hz) ppm.

^{19}F NMR (376 MHz, $CDCl_3$) δ −78.0 (s) ppm.

^{31}P{^{1}H} NMR (161 MHz, $CDCl_3$) δ 22.3 (s) ppm.

HRMS (ESI) *m/z*: $[M - OTf]^+$ calcd for $C_{27}H_{26}P$, 381.1767; found, 381.1758.

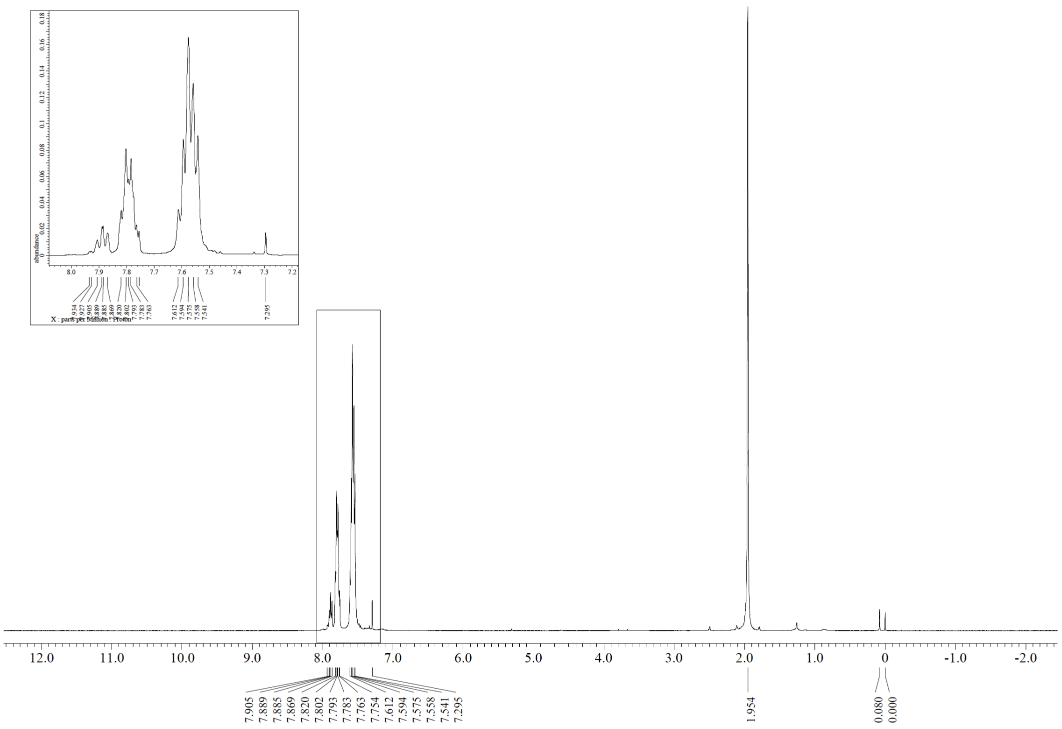

Figure S21. ^{1}H NMR spectrum of **2a**•OTf in $CDCl_3$.

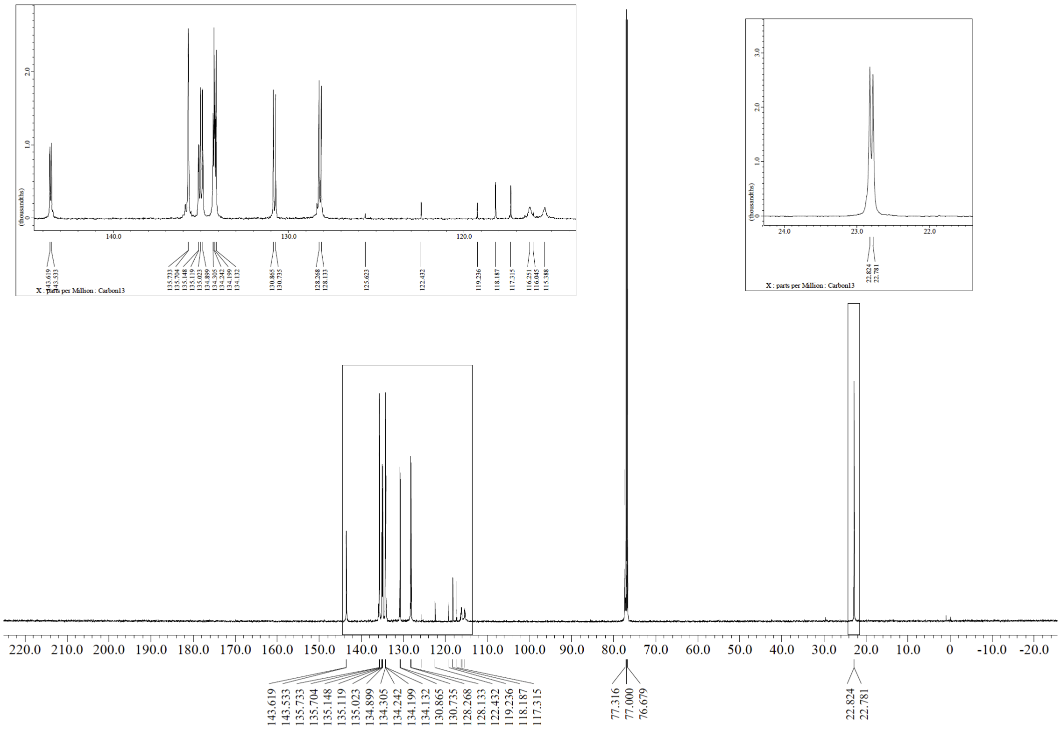

Figure S22. ^{13}C NMR spectrum of **2a**•OTf in $CDCl_3$.

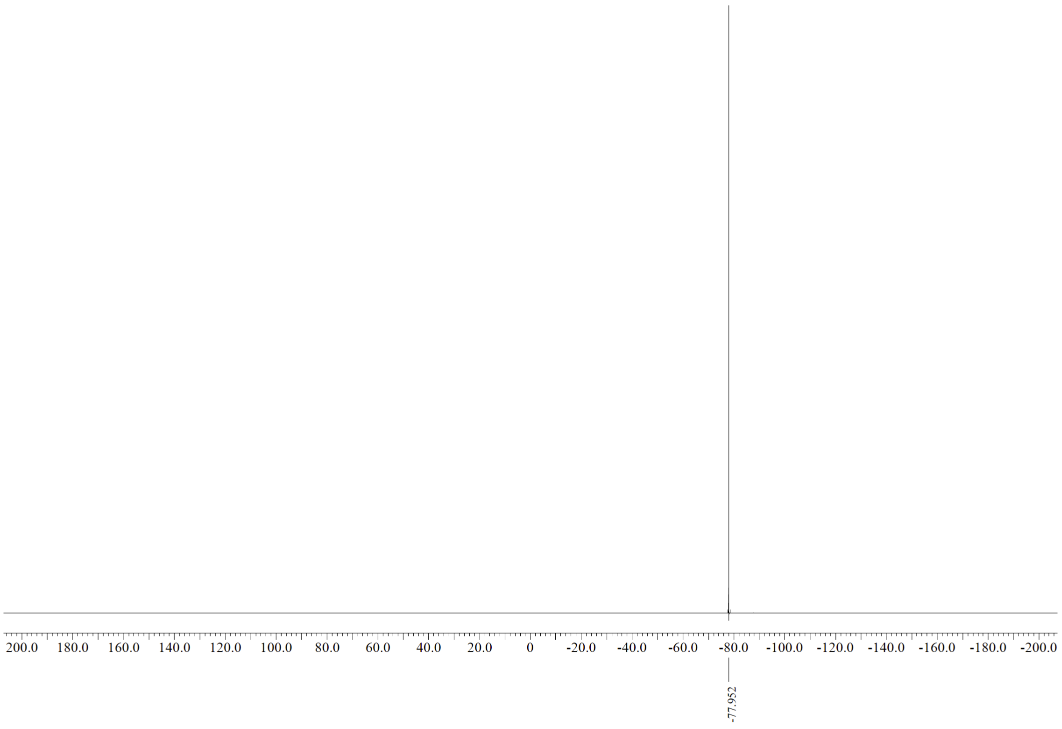

Figure S23. ^{19}F NMR spectrum of **2a**•OTf in $CDCl_3$.

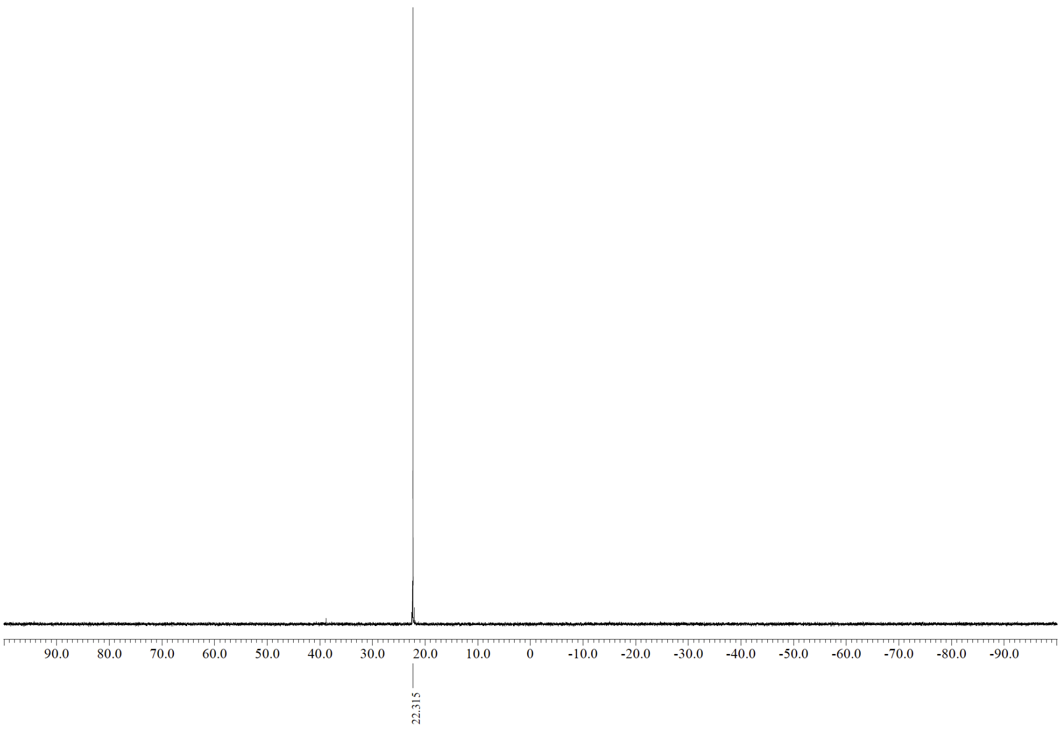

Figure S24. ^{31}P NMR spectrum of **2a**•OTf in $CDCl_3$.

(2,5-Dimethylphenyl)tri(*o*-tolyl)phosphonium triflate (2b•OTf)

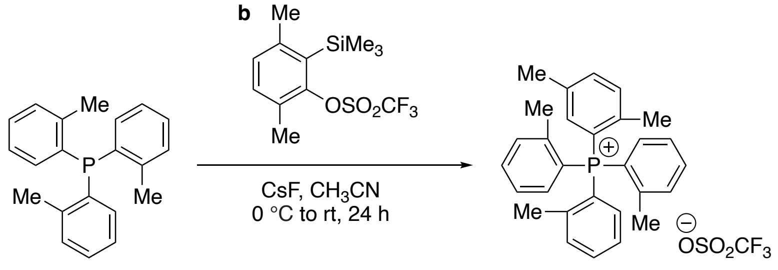

2 **2b**•OTf

According to the procedure for **2a**•OTf, **2b**•OTf was likewise prepared from tri(*o*-tolyl)phosphine (**2**, 0.090 g, 0.30 mmol, 1 eq.), CsF (0.278 g, 1.83 mmol, 6.2 eq.), and 3,6-dimethyl-2-(trimethylsilyl)phenyl triflate (**b**, 0.242 g, 0.741 mmol, 2.5 eq.) in CH_3CN (3 mL). After the purification by column chromatography (SiO_2, CH_2Cl_2 to CH_2Cl_2/methanol = 20/1 vol/vol as an eluent), **2b**•OTf was obtained as a pale orange powder (0.152 g, 0.272 mmol, 92% yield).

^{1}H NMR (400 MHz, $CDCl_3$) δ 1.85 (s, 3H), 1.90 (s, 3H), 1.92 (s, 3H), 1.95 (s, 3H), 2.37 (s, 3H), 7.35 (d, $^3J_{H–P}$ = 15.9 Hz, 1H), 7.44 (dd, $^3J_{H–H}$ = 6.8 Hz, $^4J_{H–P}$ = 6.8 Hz, 1H), 7.54–7.65 (10H), 7.77–7.83 (3H) ppm.

^{13}C{^{1}H} NMR (100 MHz, $CDCl_3$) δ 21.1, 22.3 (d, $J_{C–P}$ = 2.9 Hz), 22.8–22.9, 115.1 (d, $^1J_{C–P}$ = 84.3 Hz), 115.6 (d, $^1J_{C–P}$ = 84.8 Hz), 120.8 (q, $^1J_{C–F}$ = 321.3 Hz, *C*$F_3SO_3^-$), 128.1–128.4, 134.2–134.3, 134.9–135.2, 135.6, 136.4, 138.2 (d, $J_{C–P}$ = 13.0 Hz), 140.7 (d, $J_{C–P}$ = 8.2 Hz), 143.7–143.9 ppm.

^{19}F NMR (376 MHz, $CDCl_3$) δ −77.9 (s) ppm.

^{31}P{^{1}H} NMR (121 MHz, $CDCl_3$) δ 22.5 (s) ppm.

HRMS (ESI) *m/z*: [M − OTf]$^+$ calcd for $C_{29}H_{30}P$, 409.2080; found, 409.2069.

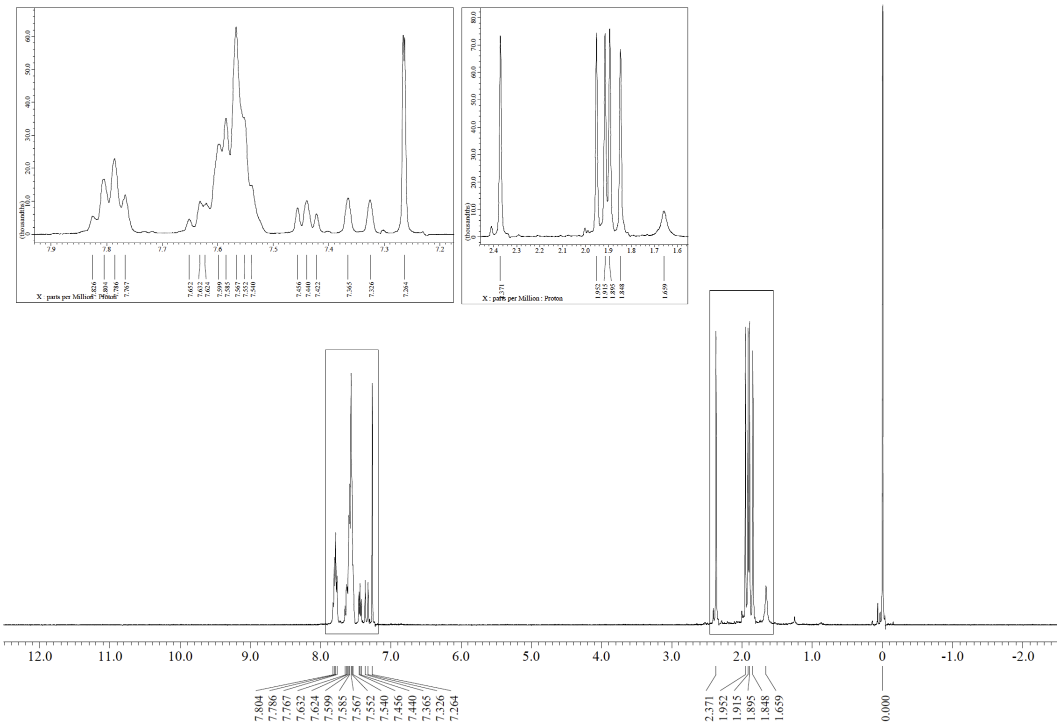

Figure S25. ^{1}H NMR spectrum of **2b**•OTf in $CDCl_3$.

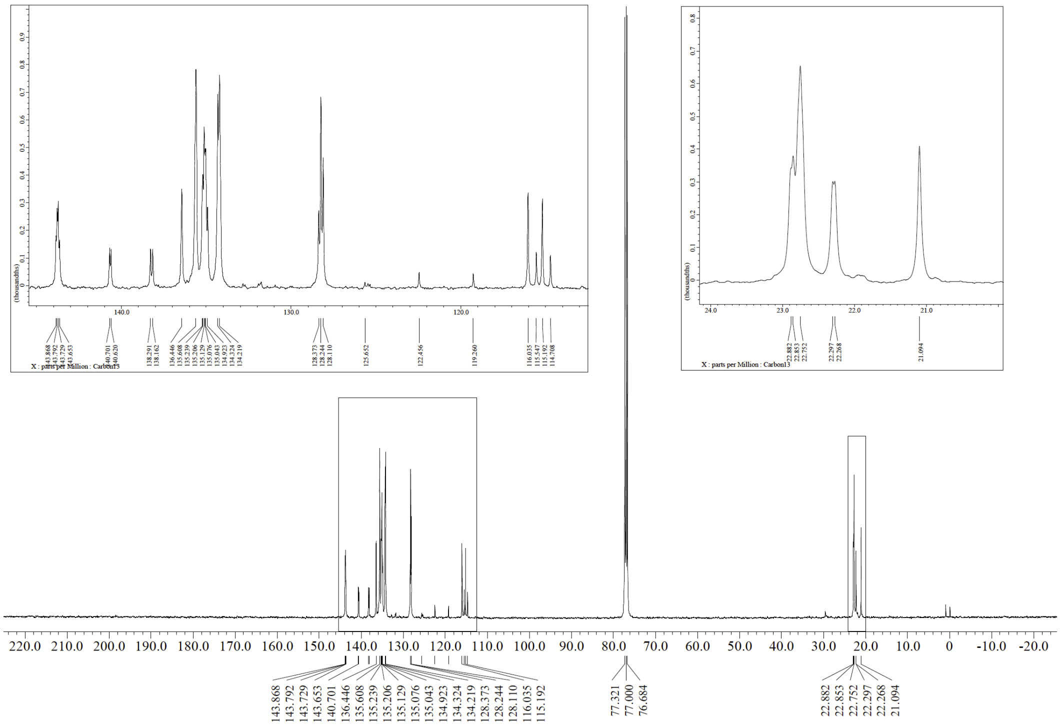

Figure S26. ^{13}C NMR spectrum of **2b**•OTf in $CDCl_3$.

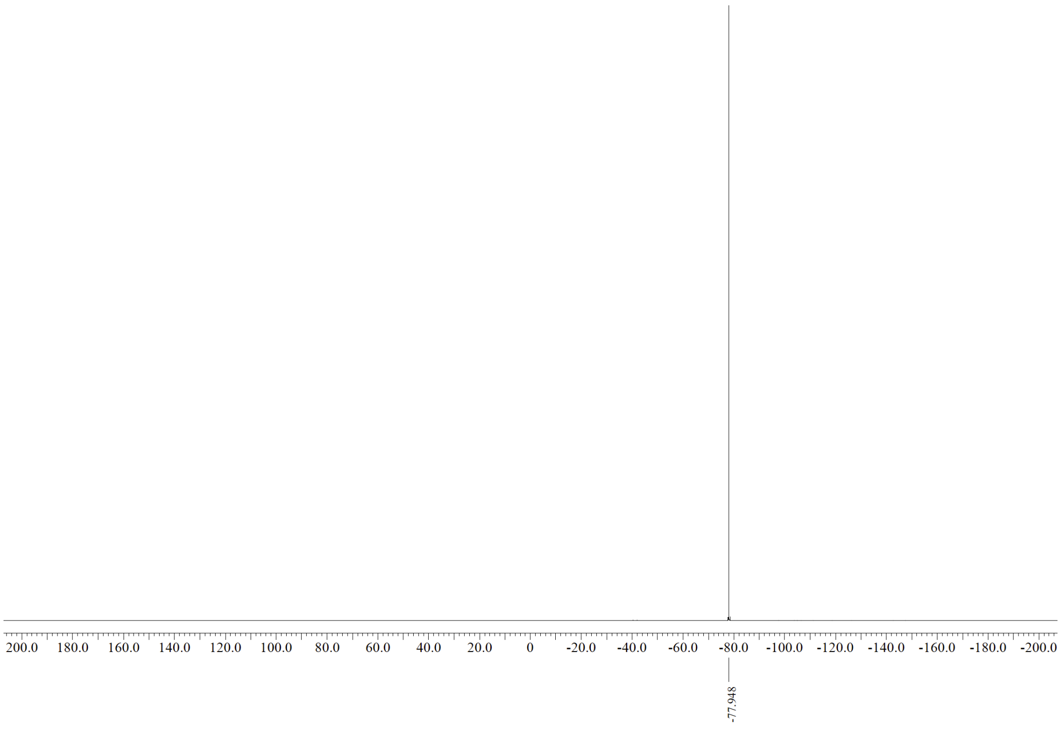

Figure S27. ^{19}F NMR spectrum of **2b**•OTf in $CDCl_3$.

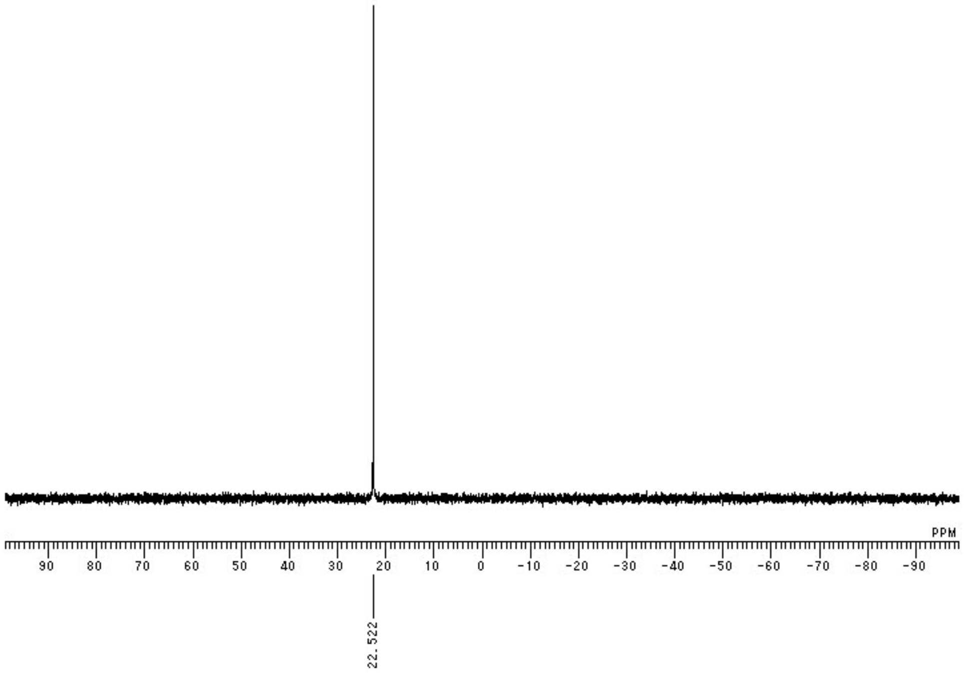

Figure S28. ^{31}P NMR spectrum of **2b**•OTf in $CDCl_3$.

Tetrakis(2,5-dimethylphenyl)phosphonium triflate (3b•OTf)

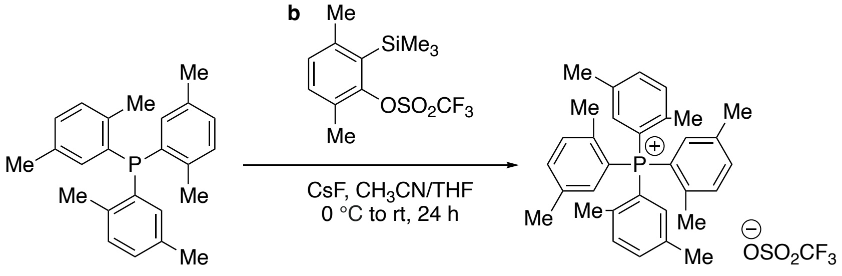

According to the procedure for **2a**•OTf, **3b**•OTf was likewise prepared from tris(2,5-dimethylphenyl)phosphine (**3**, 0.104 g, 0.300 mmol, 1 eq.), CsF (0.273 g, 1.80 mmol, 5.99 eq.), and 3,6-dimethyl-2-(trimethylsilyl)phenyl triflate (**b**, 0.490 g, 1.50 mmol, 5.00 eq.) in CH_3CN (2 mL) and THF (2 mL). After the purification by column chromatography (SiO_2, CH_2Cl_2/CH_3CN = 4/1 vol/vol as an eluent), **3b**•OTf was obtained as a pale orange powder (0.169 g, 0.281 mmol, 93.7% yield).

^{1}H NMR (400 MHz, $CDCl_3$) δ 1.86 (s, 12H), 2.37 (s, 12H), 7.31 (d, $^3J_{H–P}$ = 16.3 Hz, 4H), 7.45 (dd, $^4J_{H–P}$ = 5.9 Hz, $^3J_{H–H}$ = 7.7 Hz, 4H), 7.58 (d, $^3J_{H–H}$ = 7.8 Hz, 4H) ppm.

^{13}C{^{1}H} NMR (100 MHz, $CDCl_3$) δ 21.2, 22.3 (d, $^3J_{C–P}$ = 4.3 Hz), 115.6 (d, $^1J_{C–P}$ = 83.8 Hz), 134.2 (d, $J_{C–P}$ = 12.0 Hz), 135.1 (d, $J_{C–P}$ = 12.5 Hz), 136.4 (d, $^4J_{C–P}$ = 2.9 Hz), 138.3 (d, $J_{C–P}$ = 13.0 Hz), 140.6 (d, $J_{C–P}$ = 8.2 Hz) ppm.

^{19}F NMR (376 MHz, $CDCl_3$) δ −78.0 (s) ppm.

^{31}P{^{1}H} NMR (161 MHz, $CDCl_3$) δ 22.0 (s) ppm.

HRMS (ESI) *m/z*: [M − OTf]$^+$ calcd for $C_{32}H_{36}P$, 451.2549; found, 451.2546.

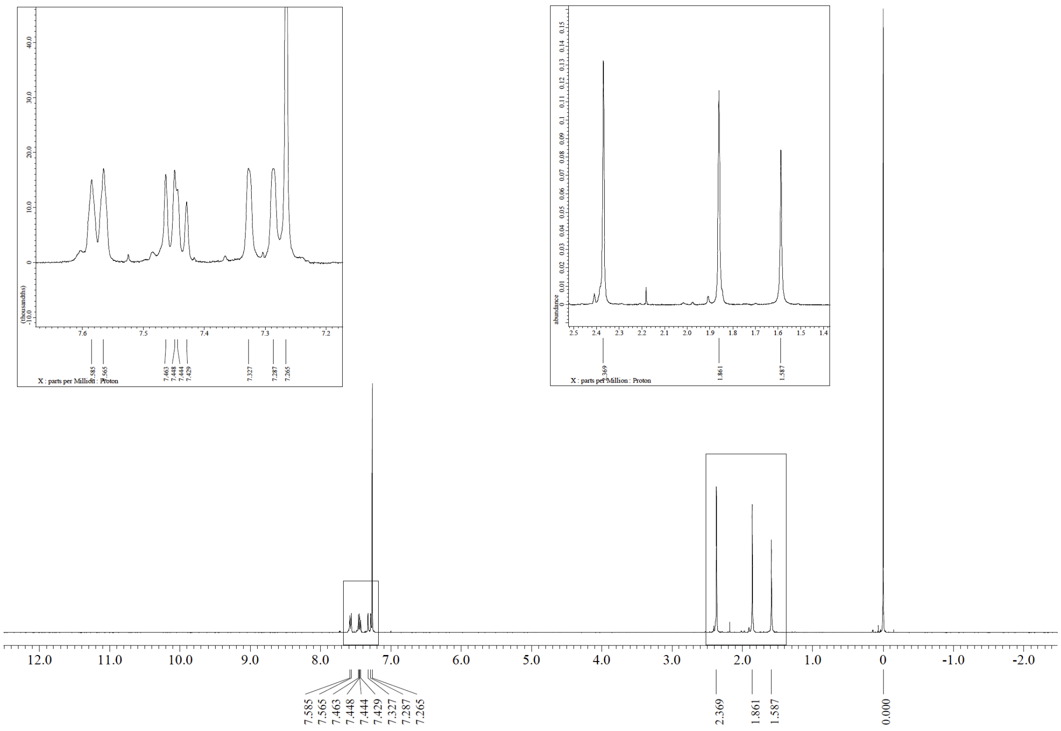

Figure S29. ^{1}H NMR spectrum of **3b**•OTf in $CDCl_3$.

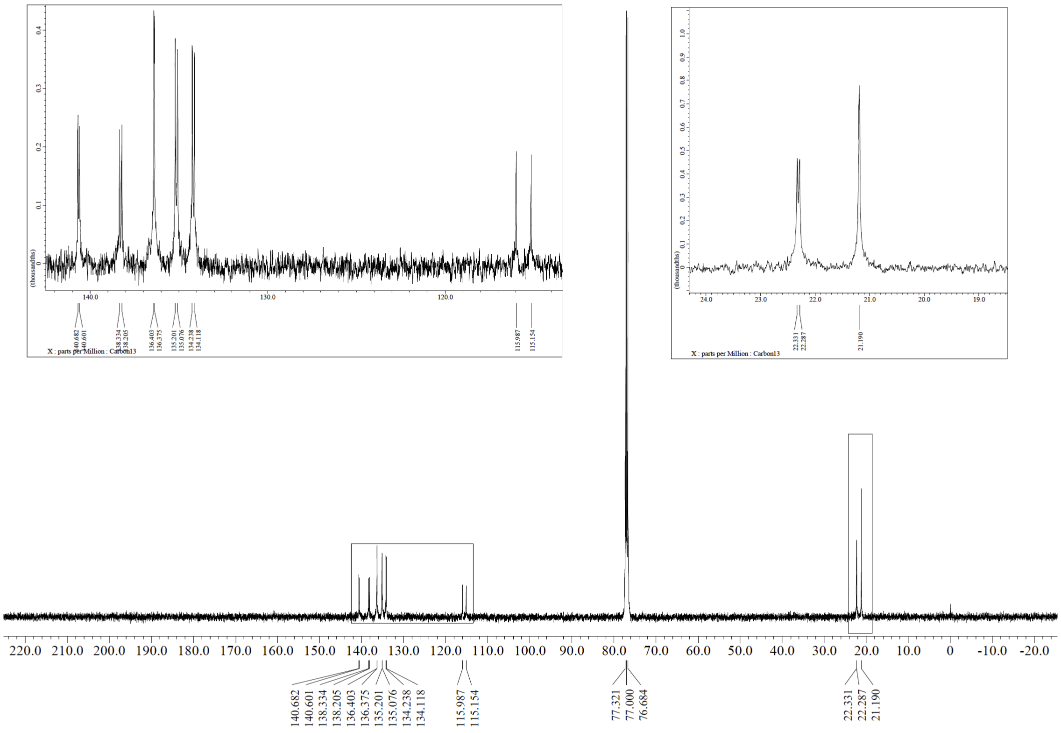

Figure S30. ^{13}C NMR spectrum of **3b**•OTf in $CDCl_3$.

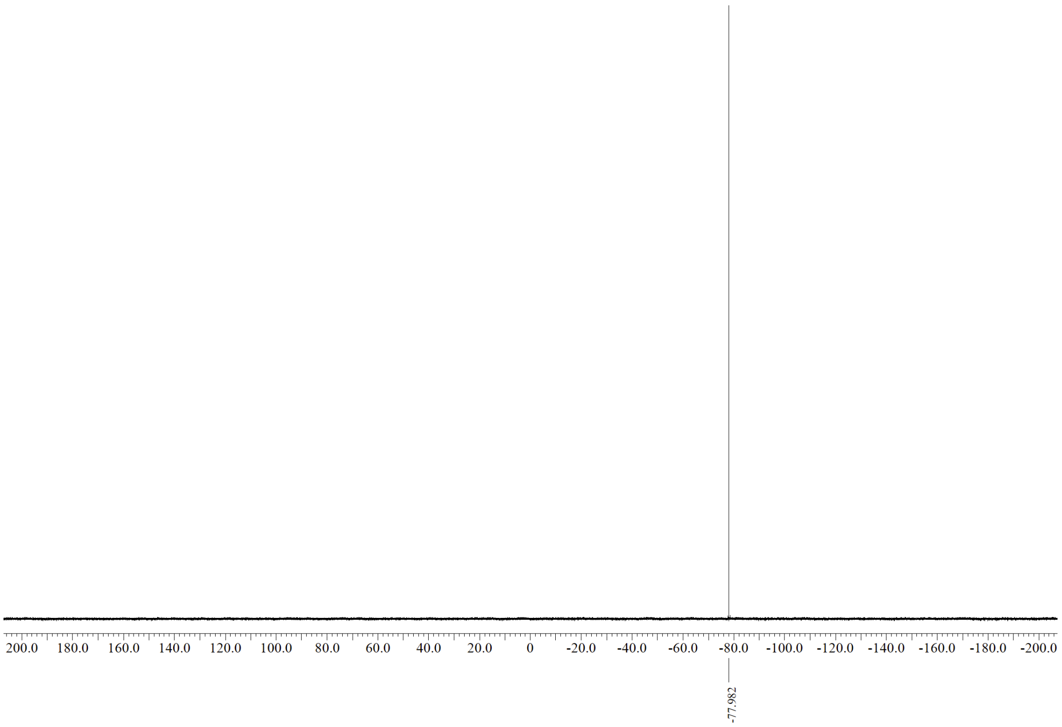

Figure S31. ^{19}F NMR spectrum of **3b**•OTf in $CDCl_3$.

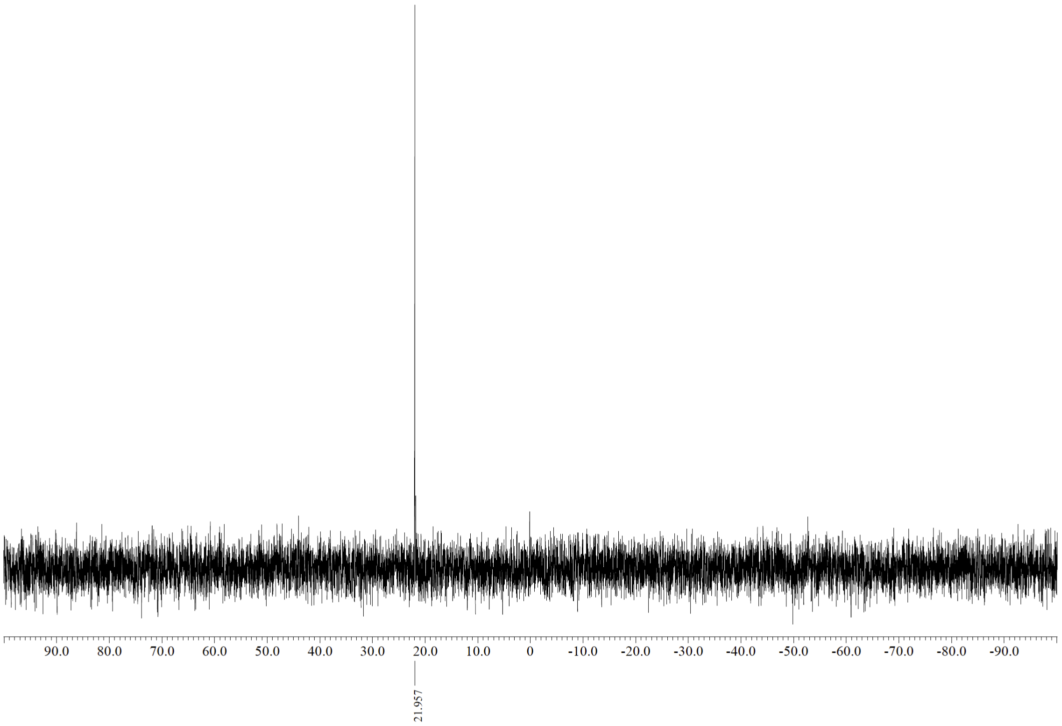

Figure S32. ^{31}P NMR spectrum of **3b**•OTf in $CDCl_3$.

Tris(2,5-dimethylphenyl)(2,4,6-trimethylphenyl)phosphonium triflate (4b•OTf)

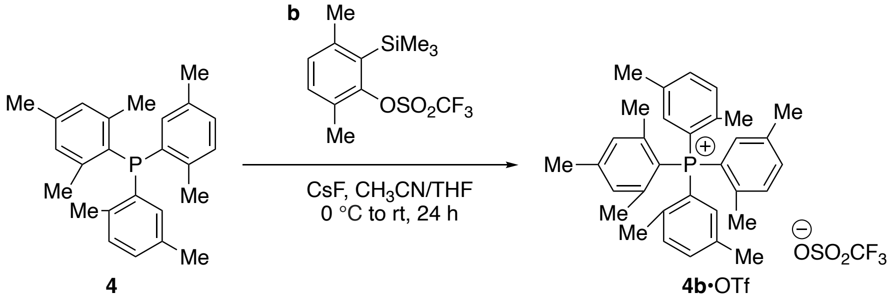

According to the procedure for **2a**•OTf, **4b**•OTf was likewise prepared from bis(2,5-dimethylphenyl)(2,4,6-trimethylphenyl)phosphine (**4**, 0.108 g, 0.300 mmol, 1 eq.), CsF (0.182 g, 1.20 mmol, 4.00 eq.), and 3,6-dimethyl-2-(trimethylsilyl)phenyl triflate (**b**, 0.391 g, 1.20 mmol, 4.00 eq.) in CH_3CN (1.5 mL) and THF (1.5 mL). After the purification by column chromatography (SiO_2, CH_2Cl_2/CH_3CN = 9/1 vol/vol as an eluent), **4b**•OTf was obtained as a pale orange powder (0.180 g, 0.293 mmol, 97.7% yield).

^{1}H NMR (300 MHz, $CDCl_3$) δ 1.72–1.74 (6H), 1.82 (s, 3H), 2.10–2.14 (6H), 2.34–2.42 (12H), 7.13–7.14 (2H), 7.30–7.56 (9H) ppm.

^{13}C{^{1}H} NMR (100 MHz, $CDCl_3$) δ 21.0 (d, J_{C-P} = 2.4 Hz), 21.1 (d, J_{C-P} = 3.4 Hz), 22.0 (d, J_{C-P} = 4.8 Hz), 22.4 (d, J_{C-P} = 3.4 Hz), 22.6–22.7, 25.3 (d, J_{C-P} = 4.8 Hz), 110.9, 111.8, 116.4, 117.3, 117.6, 118. 5, 119.3, 120.0, 120.8, 122.5, 133.4 (d, J_{C-P} = 11.6 Hz), 133.9–134.5, 135.2 (d, J_{C-P} = 12 .0 Hz), 135.9–136.0, 137.5–137.9, 140.0 (d, J_{C-P} = 8.2 Hz), 140.9–141.1, 144.4 (d, J_{C-P} = 9.6 Hz), 144.9 (d, J_{C-P} = 10.1 Hz), 145.9 (d, J_{C-P} = 3.4 Hz) ppm.

^{19}F NMR (376 MHz, $CDCl_3$) δ −78.0 (s) ppm.

^{31}P{^{1}H} NMR (161 MHz, $CDCl_3$) δ 18.0 (s) ppm.

HRMS (ESI) *m/z*: $[M - OTf]^+$ calcd for $C_{33}H_{38}P$, 465.2706; found, 465.2694.

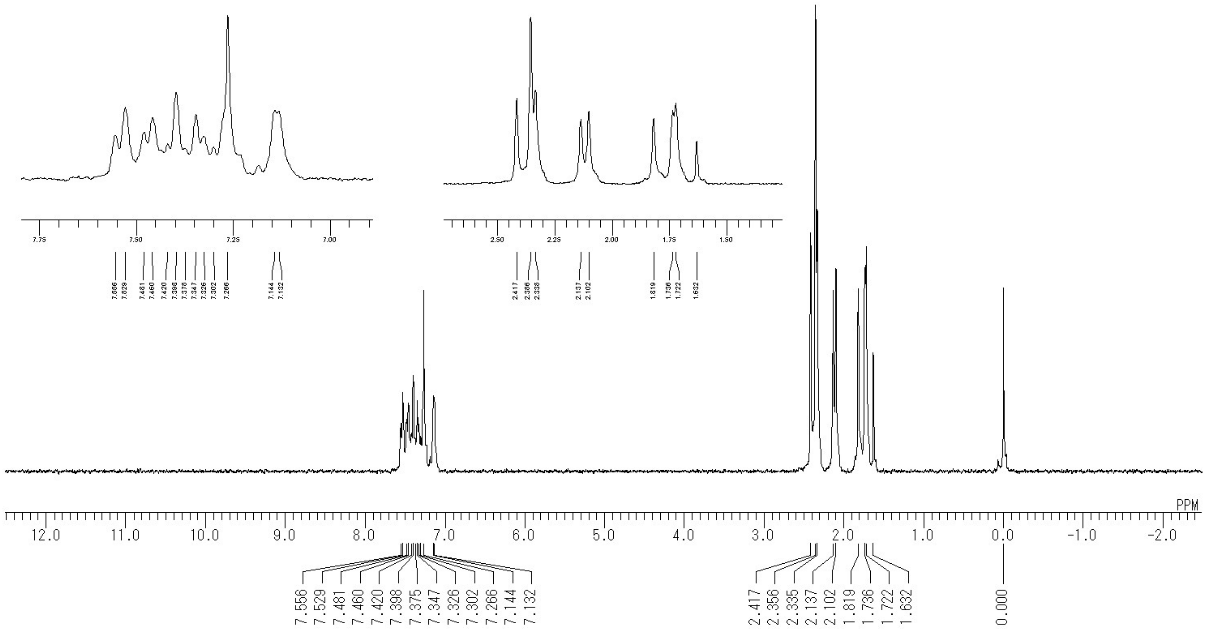

Figure S33. ^1H NMR spectrum of **4b**•OTf in $CDCl_3$.

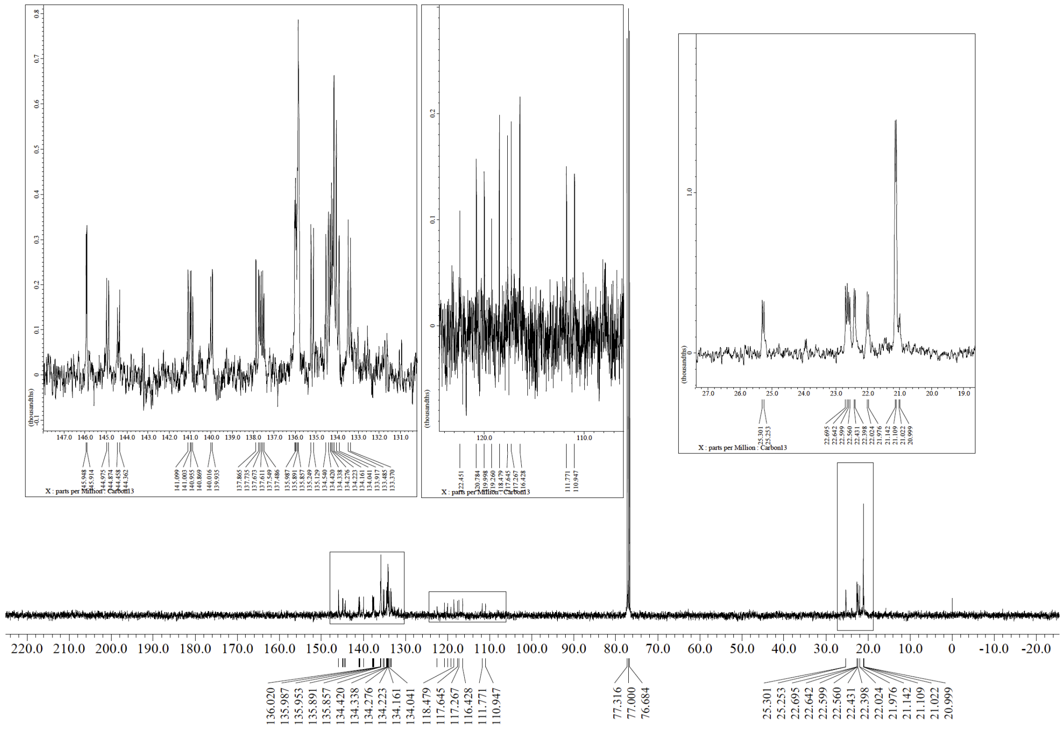

Figure S34. ^{13}C NMR spectrum of **4b**•OTf in $CDCl_3$.

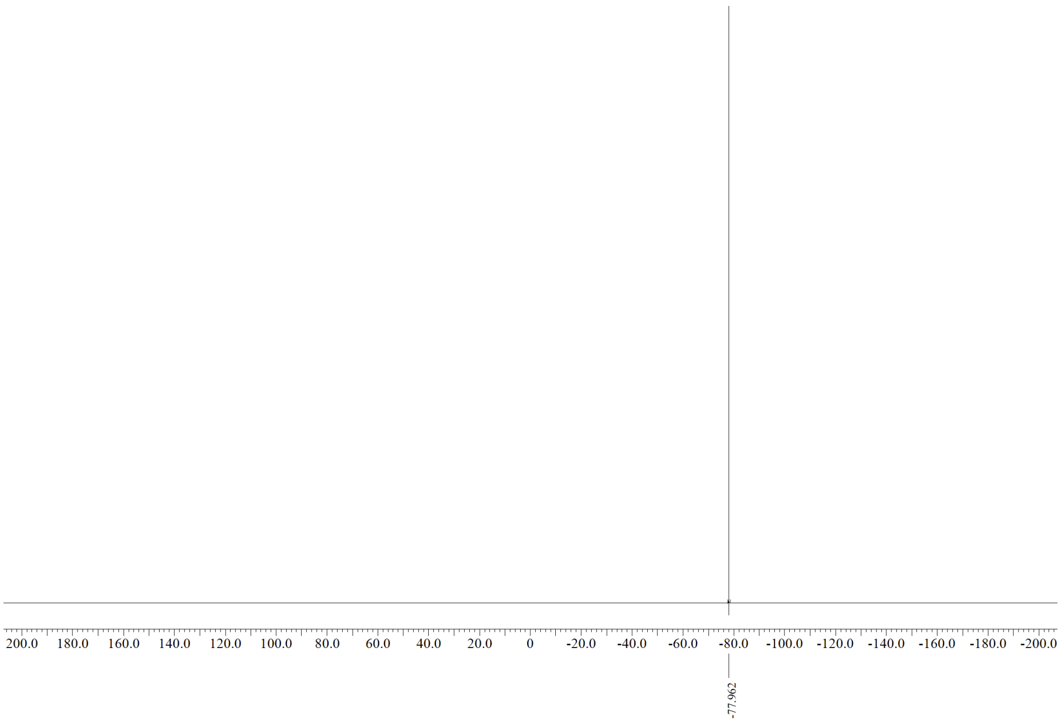

Figure S35. ^{19}F NMR spectrum of **4b**•OTf in $CDCl_3$.

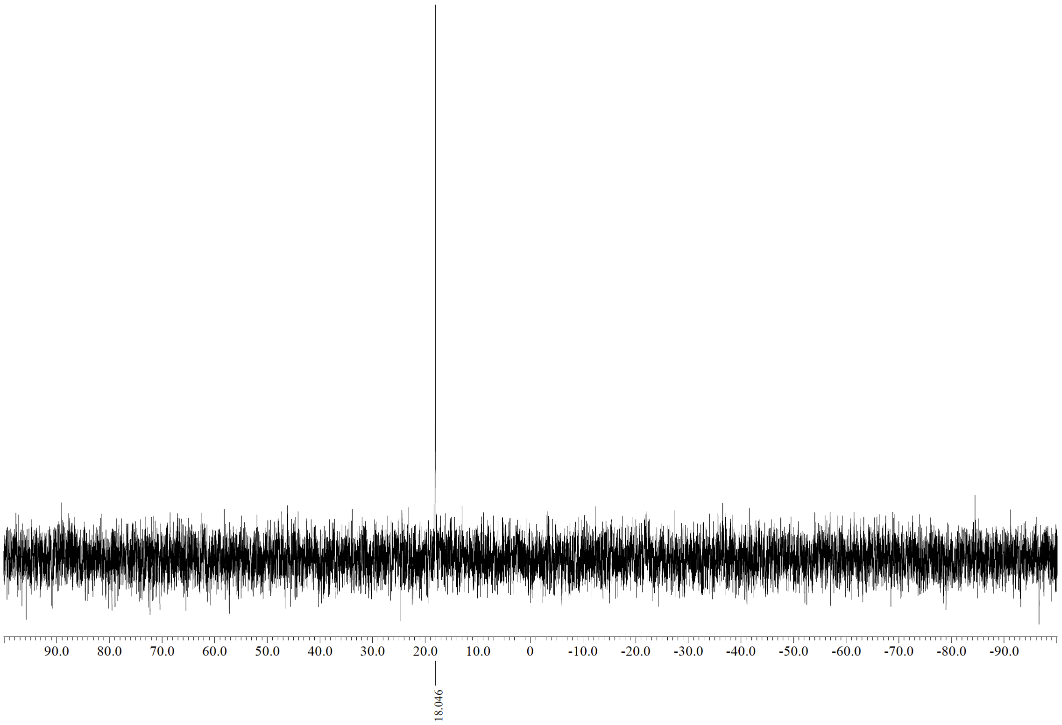

Figure S36. ^{31}P NMR spectrum of **4b**•OTf in $CDCl_3$.

Bis(2,5-dimethylphenyl)bis(2,4,6-trimethylphenyl)phosphonium triflate (5b•OTf)

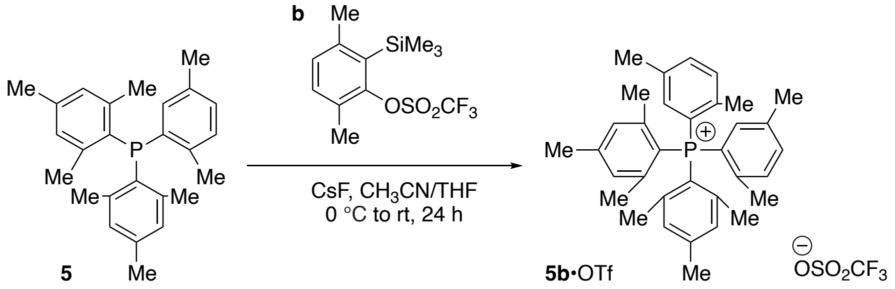

According to the procedure for **2a**•OTf, **5b**•OTf was likewise prepared from (2,5-dimethylphenyl)bis(2,4,6-trimethylphenyl)phosphine (**5**, 0.112 g, 0.299 mmol, 1 eq.), CsF (0.136 g, 0.895 mmol, 2.99 eq.), and 3,6-dimethyl-2-(trimethylsilyl)phenyl triflate (**b**, 0.294 g, 0.901 mmol, 3.01 eq.) in CH_3CN (2 mL) and THF (2 mL). After the purification by column chromatography (SiO_2, CH_2Cl_2/CH_3CN = 3/1 vol/vol as an eluent), **5b**•OTf was obtained as a pale orange powder (0.183 g, 0.291 mmol, 97.3% yield).

^{1}H NMR (400 MHz, $CDCl_3$) δ 1.72 (s, 3H), 1.94 (s, 3H), 2.07–2.20 (12H), 2.33–2.42 (12H), 7.01–7.26 (5H), 7.31–7.35 (1H), 7.38–7.51 (4H) ppm.

^{13}C{^{1}H} NMR (100 MHz, $CDCl_3$) δ 20.8–21.0, 21.6 (d, $J_{C–P}$ = 4.3 Hz), 22.2–22.5, 22.9 (d, $J_{C–P}$ = 5.3 Hz), 23.1 (d, $J_{C–P}$ = 4.3 Hz), 23.6, 24.3–24.5, 25.1, 29.4, 112.5, 113.3, 113.7, 114.6, 115.9, 117.0, 117.8, 118.9, 119.1, 119.7, 121.3, 121.4, 122.1, 122.2, 122.3, 125.5, 130.9 (d, $J_{C–P}$ = 11.1 Hz), 133.0–133.4, 133.7–134.3, 134.6 (d, $J_{C–P}$ = 11.6 Hz), 135.1–135.5, 136.1–136.3, 136.9 (d, $J_{C–P}$ = 12.5 Hz), 137.3 (d, $J_{C–P}$ = 12.5 Hz), 140.1 (d, $J_{C–P}$ = 8.7 Hz), 140.9 (d, $J_{C–P}$ = 9.6 Hz), 141.8 (d, $J_{C–P}$ = 9.6 Hz), 142.2 (d, $J_{C–P}$ = 10.1 Hz), 143.6 (d, $J_{C–P}$ = 9.6 Hz), 144.2–144.7, 145.0–145.5 ppm.

^{19}F NMR (376 MHz, $CDCl_3$) δ −77.9 (s) ppm.

^{31}P{^{1}H} NMR (161 MHz, $CDCl_3$) δ 14.7, 15.2, 16.1, 17.3 ppm.

HRMS (ESI) *m/z*: [M − OTf]$^+$ calcd for $C_{34}H_{40}P$, 479.2862; found, 479.2858.

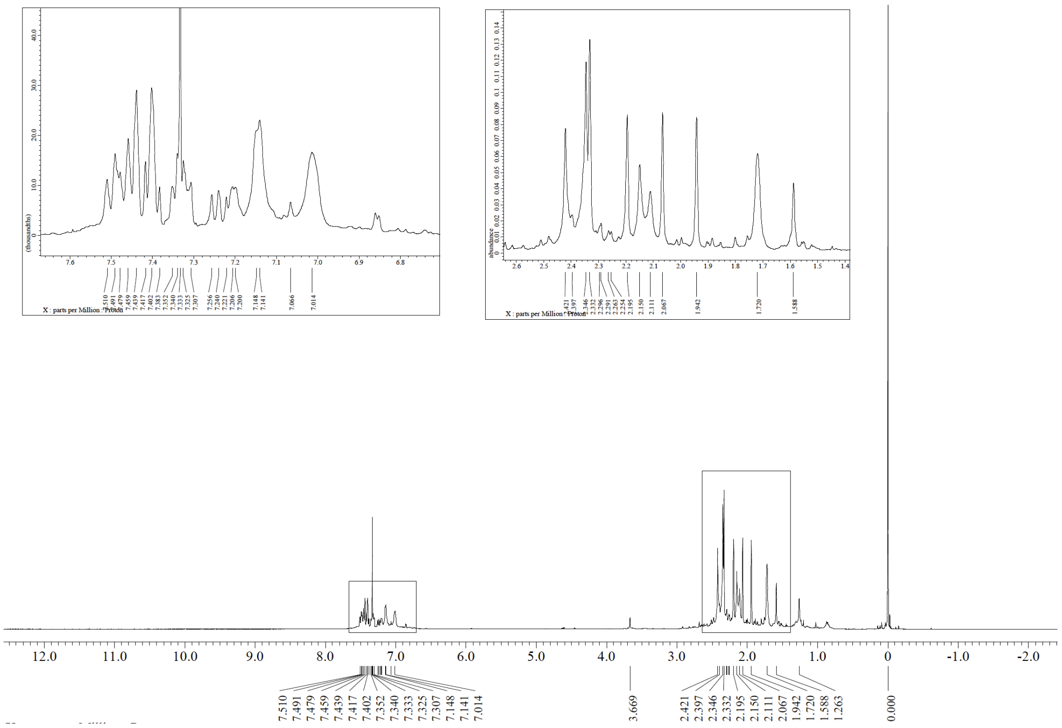

Figure S37. ^{1}H NMR spectrum of **5b**•OTf in $CDCl_3$.

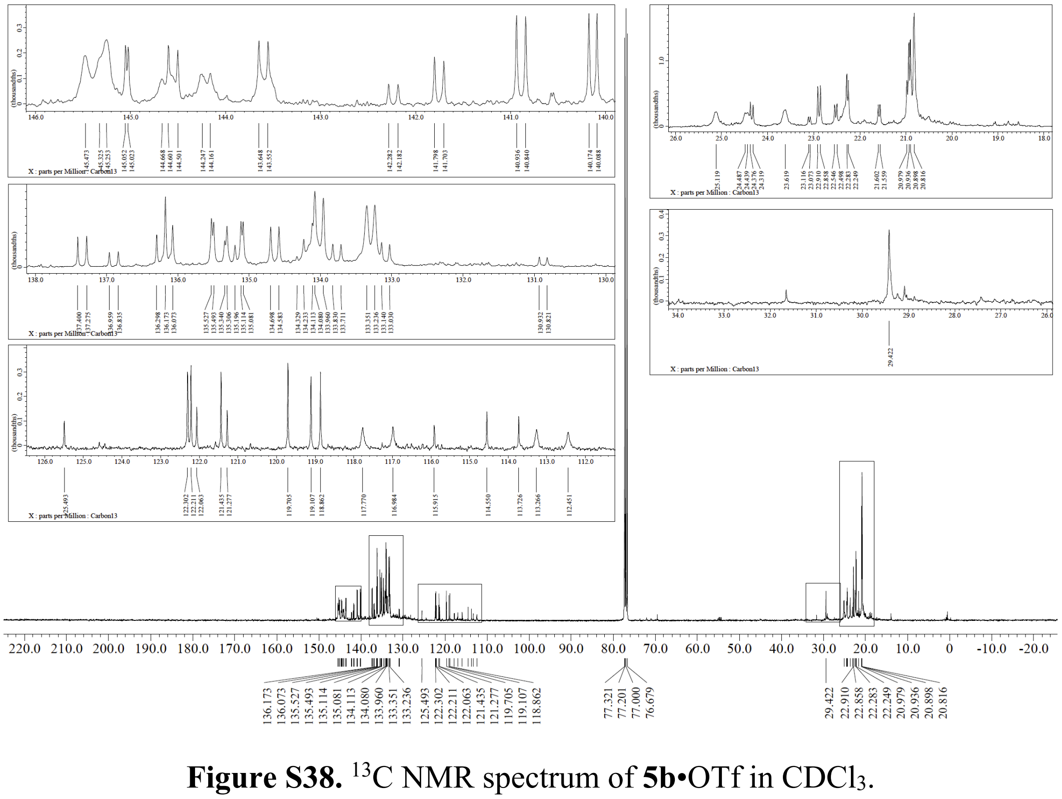

Figure S38. ^{13}C NMR spectrum of **5b**•OTf in $CDCl_3$.

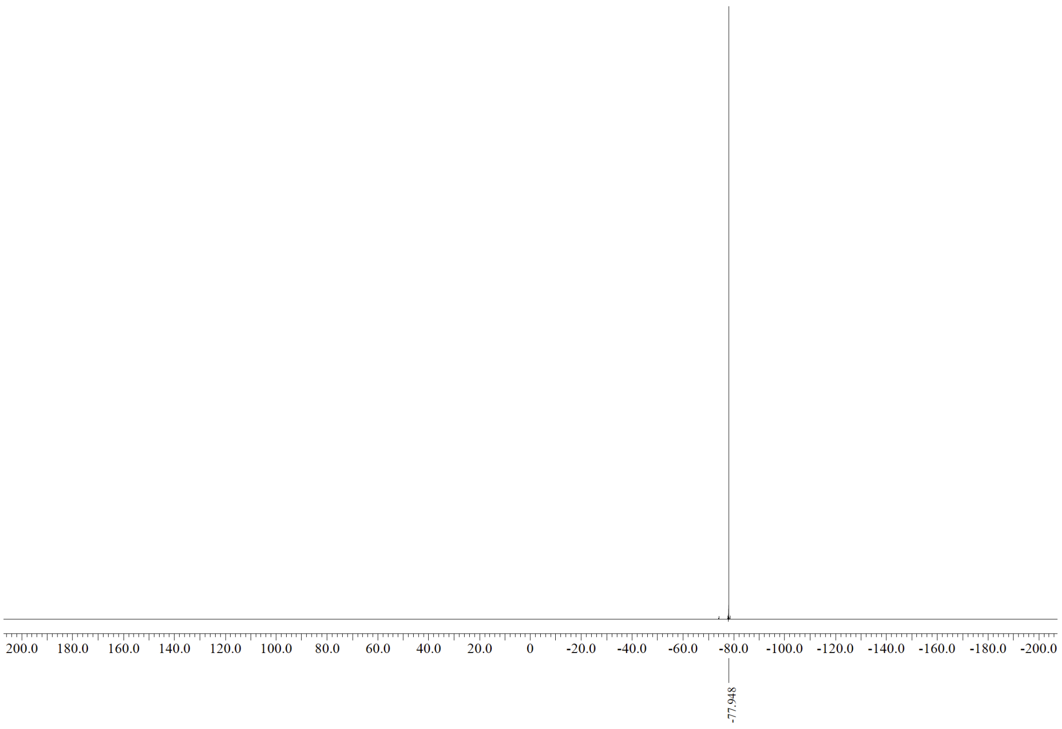

Figure S39. ^{19}F NMR spectrum of **5b•**OTf in $CDCl_3$.

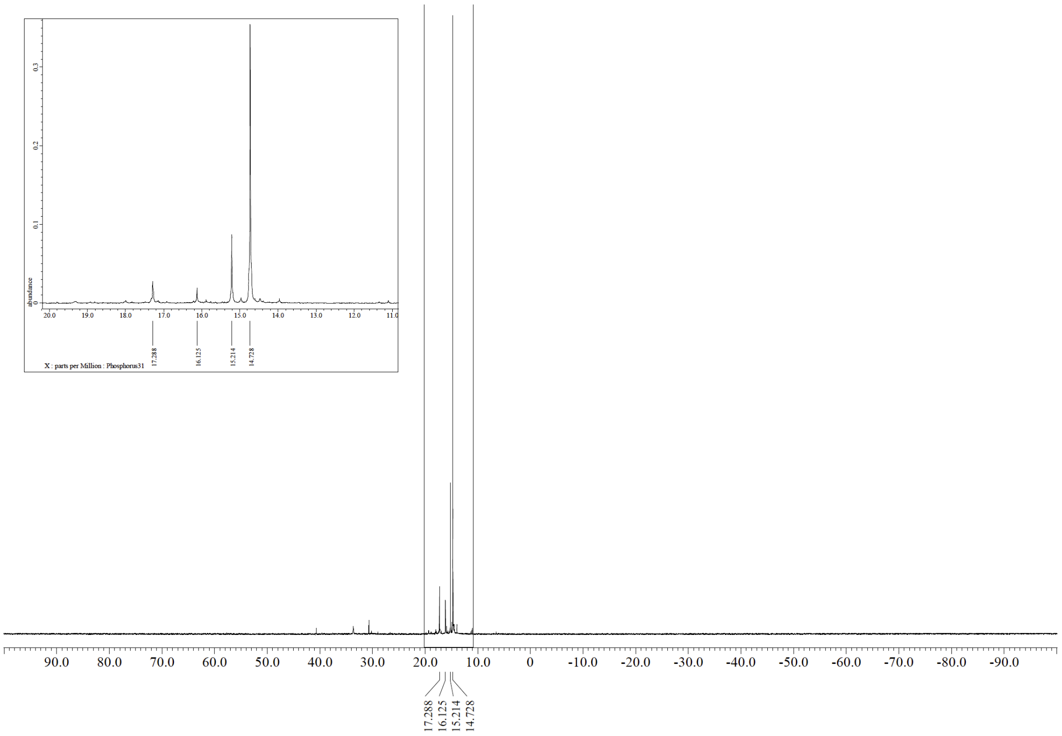

Figure S40. ^{31}P NMR spectrum of **5b•**OTf in $CDCl_3$.

Tris(2,6-dimethylphenyl)(phenyl)phosphonium triflate (6a•OTf)

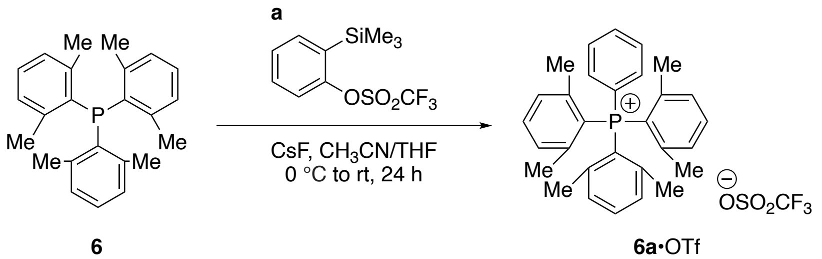

According to the procedure for **2a**•OTf, **6a**•OTf was likewise prepared from tris(2,6-dimethylphenyl)phosphine (**6**, 0.104 g, 0.300 mmol, 1 eq.), CsF (0.137 g, 0.902 mmol, 3.00 eq.), and 2-(trimethylsilyl)phenyl triflate (**a**, 0.268 g, 0.898 mmol, 2.99 eq.) in CH_3CN (1.5 mL) and THF (1.5 mL). After the purification by column chromatography (SiO_2, CH_2Cl_2/CH_3CN = 9/1 vol/vol as an eluent), **6a**•OTf was obtained as a pale orange powder (0.157 g, 0.274 mmol, 91.3% yield).

^{1}H NMR (400 MHz, $CDCl_3$) δ 1.90 (s, 9H), 2.23 (s, 9H), 7.23–7.31 (6H), 7.55–7.65 (7H), 7.70–7.74 (m, 1H) ppm.

^{13}C{^{1}H} NMR (100 MHz, $CDCl_3$) δ 23.9 (d, $^3J_{C-P}$ = 5.3 Hz), 25.4 (d, $^3J_{C-P}$ = 5.3 Hz), 120.9 (q, $^1J_{C-F}$ = 320.9 Hz, $\mathit{C}F_3SO_3^-$), 122.0 (d, $^1J_{C-P}$ = 77.5 Hz), 123.9 (d, $^1J_{C-P}$ = 81.4 Hz), 129.5 (d, J_{C-P} = 12.5 Hz), 132.6 (d, J_{C-P} = 10.6 Hz), 132.7 (d, J_{C-P} = 11.1 Hz), 134.3 (d, $^4J_{C-P}$ = 2.9 Hz), 134.5 (d, $^4J_{C-P}$ = 2.9 Hz), 136.8 (d, J_{C-P} = 10.6 Hz), 144.3 (d, J_{C-P} = 9.6 Hz), 144.6 (d, J_{C-P} = 10.1 Hz) ppm.

^{19}F NMR (376 MHz, $CDCl_3$) δ −77.9 (s) ppm.

^{31}P{^{1}H} NMR (161 MHz, $CDCl_3$) δ 14.9 (s) ppm.

HRMS (ESI) *m/z*: [M − OTf]$^+$ calcd for $C_{30}H_{32}P$, 423.2236; found, 423.2231.

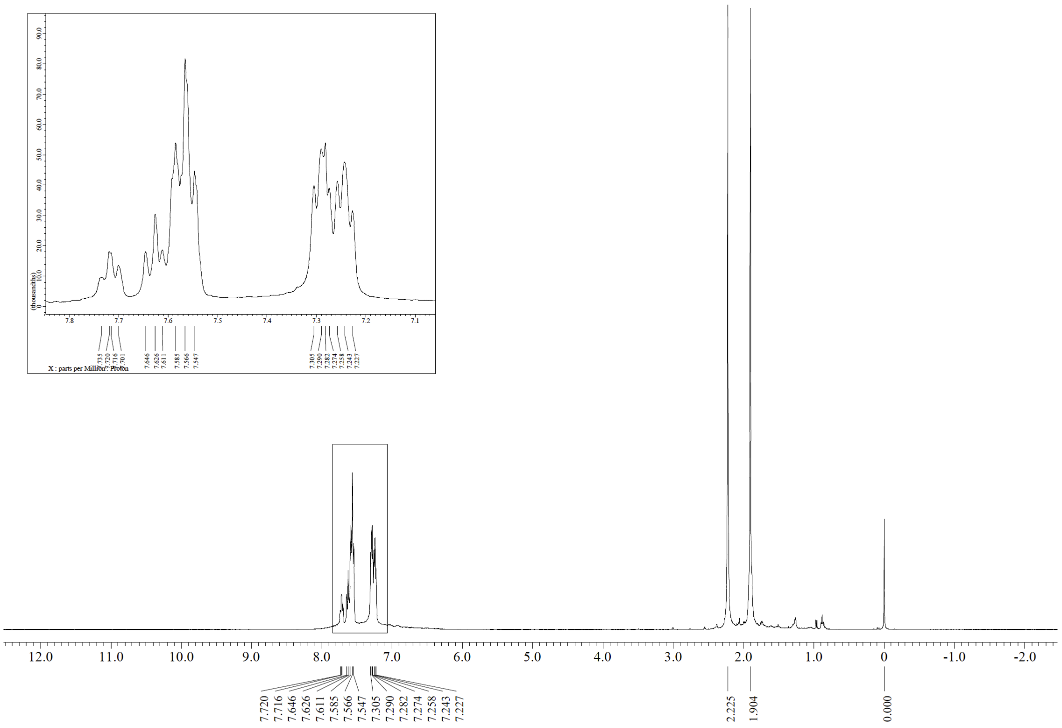

Figure S41. ^{1}H NMR spectrum of **6a**•OTf in $CDCl_3$.

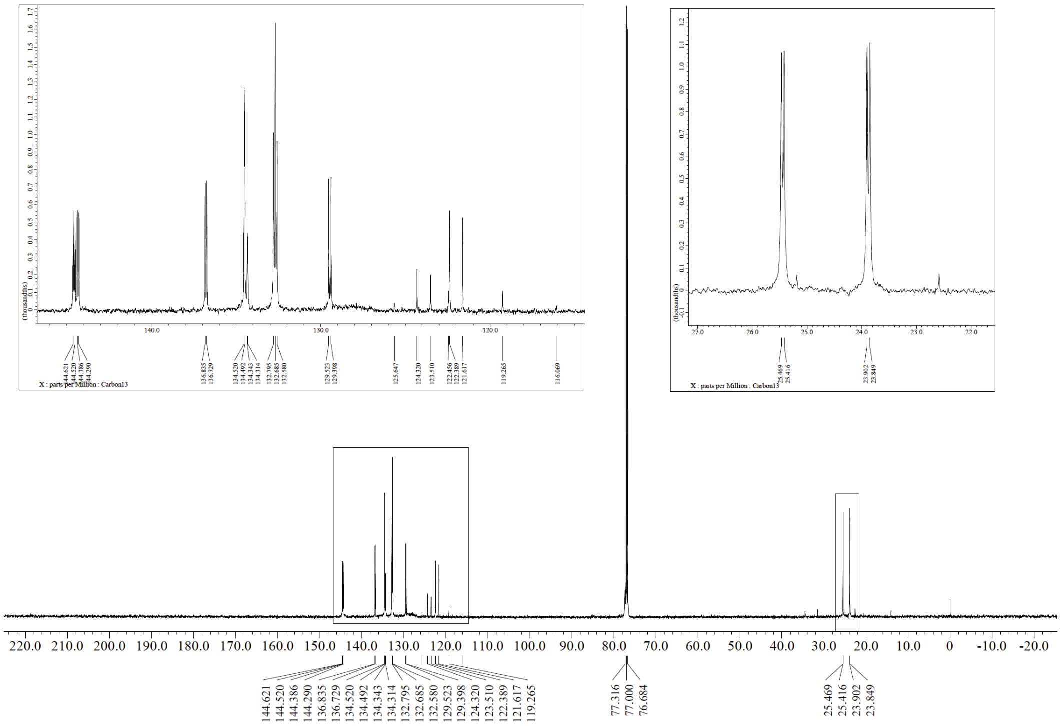

Figure S42. ^{13}C NMR spectrum of **6a**•OTf in $CDCl_3$.

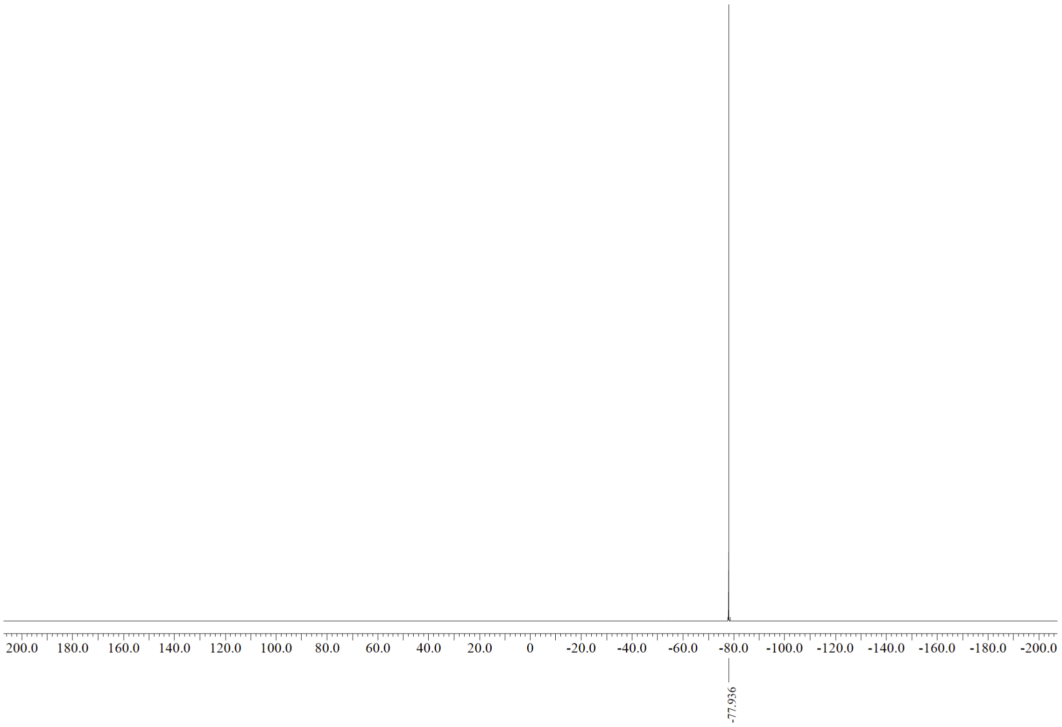

Figure S43. ^{19}F NMR spectrum of **6a**•OTf in $CDCl_3$.

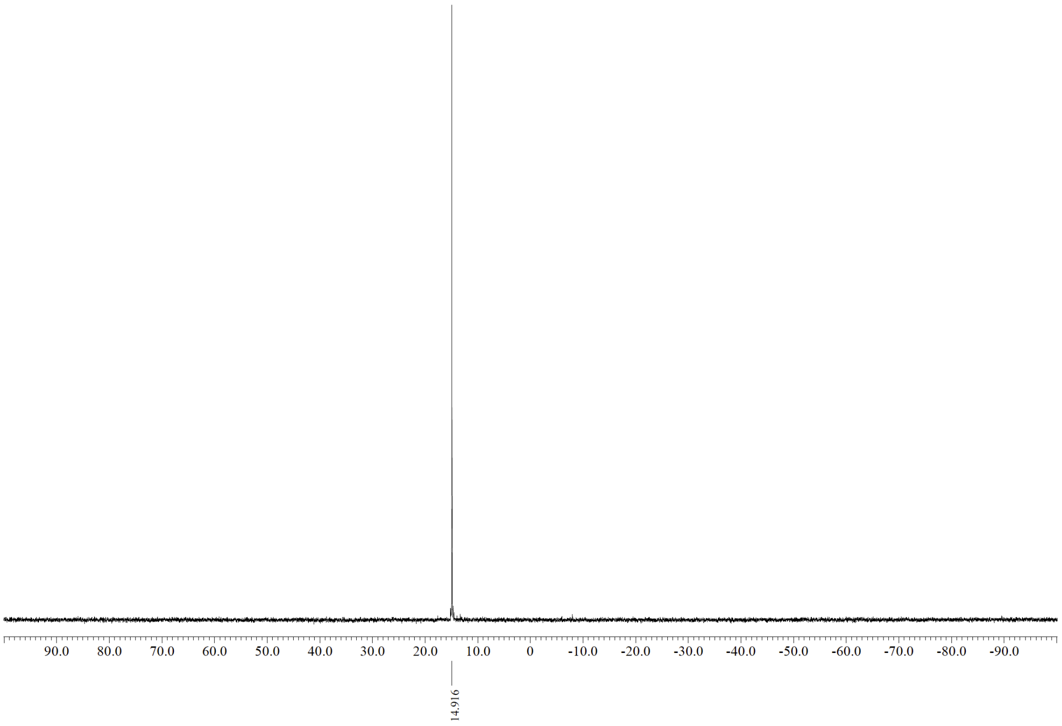

Figure S44. ^{31}P NMR spectrum of **6a**•OTf in $CDCl_3$.

(Phenyl)tris(2,4,6-trimethylphenyl)phosphonium triflate (7a•OTf)

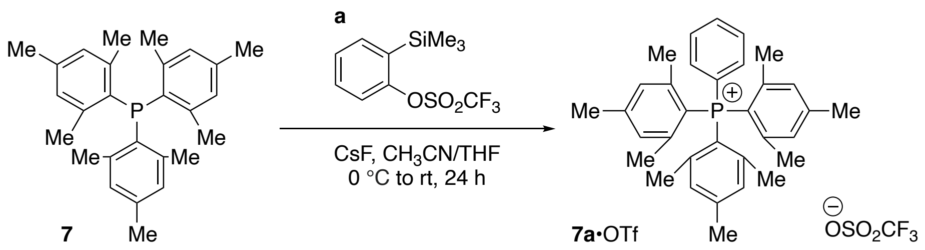

According to the procedure for **2a**•OTf, **7a**•OTf was likewise prepared from tris(2,4,6-trimethylphenyl)phosphine (**7**, 0.116 g, 0.299 mmol, 1 eq.), CsF (0.137 g, 0.902 mmol, 3.02 eq.), and 2-(trimethylsilyl)phenyl triflate (**a**, 0.268 g, 0.898 mmol, 3.01 eq.) in CH_3CN (2 mL) and THF (2 mL). After the purification by column chromatography (SiO_2, CH_2Cl_2/CH_3CN = 9/1 vol/vol as an eluent), **7a**•OTf was obtained as a pale orange powder (0.161 g, 0.262 mmol, 87.7% yield).

^1H NMR (400 MHz, $CDCl_3$) δ 1.84 (s, 9H), 2.17 (s, 9H), 2.37 (s, 9H), 7.03 (d, $J_{H–P}$ = 4.2 Hz, 3H), 7.07 (d, $J_{H–P}$ = 4.6 Hz, 3H), 7.48–7.62 (4H), 7.66–7.71 (m, 1H) ppm.

$^{13}C\{^1H\}$ NMR (100 MHz, $CDCl_3$) δ 21.0 (d, $^5J_{C–P}$ = 1.0 Hz), 23.7 (d, $^3J_{C–P}$ = 5.3 Hz), 25.3 (d, $^3J_{C–P}$ = 4.8 Hz), 118.7 (d, $^1J_{C–P}$ = 80.4 Hz), 124.8 (d, $^1J_{C–P}$ = 81.9 Hz), 129.3 (d, $J_{C–P}$ = 12.5 Hz), 133.4–133.6 (overlapping signals), 134.0 (d, $^4J_{C–P}$ = 3.4 Hz), 136.7 (d, $J_{C–P}$ = 10.6 Hz), 144.1 (d, $J_{C–P}$ = 10.1 Hz), 144.4 (d, $J_{C–P}$ = 10.6 Hz), 145.3 (d, $^4J_{C–P}$ = 3.4 Hz) ppm.

^{19}F NMR (376 MHz, $CDCl_3$) δ −78.0 (s) ppm.

$^{31}P\{^1H\}$ NMR (161 MHz, $CDCl_3$) δ 14.5 (s) ppm.

HRMS (ESI) *m/z*: $[M - OTf]^+$ calcd for $C_{33}H_{38}P$, 465.2706; found, 465.2709.

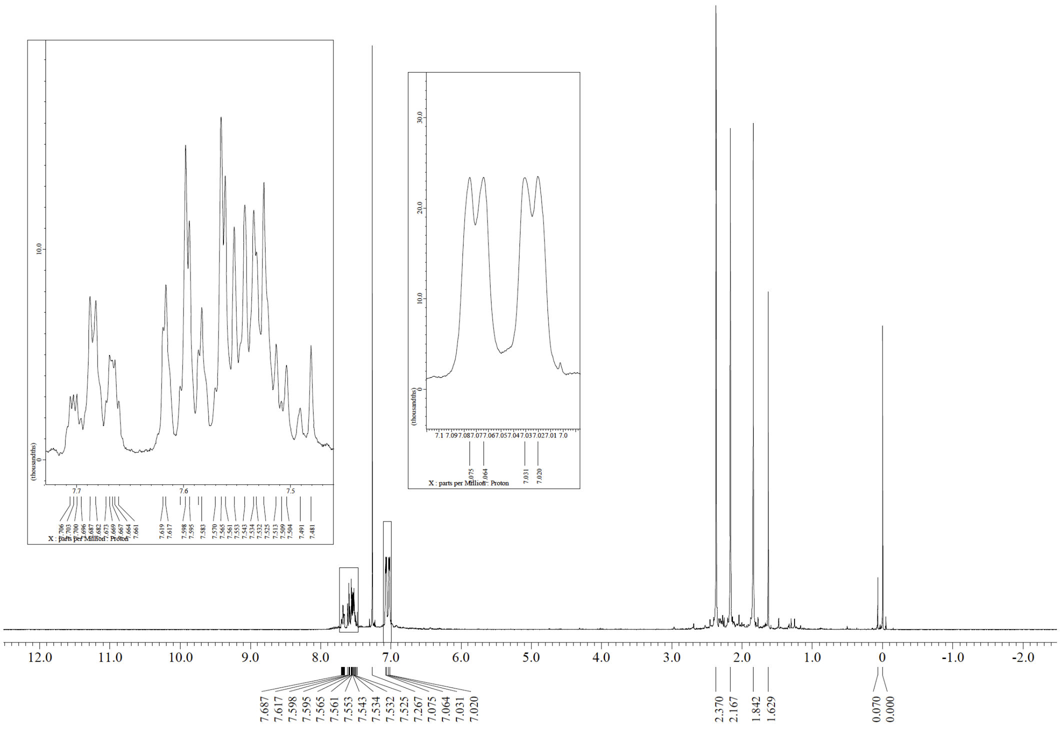

Figure S45. ^{1}H NMR spectrum of **7a**•OTf in $CDCl_3$.

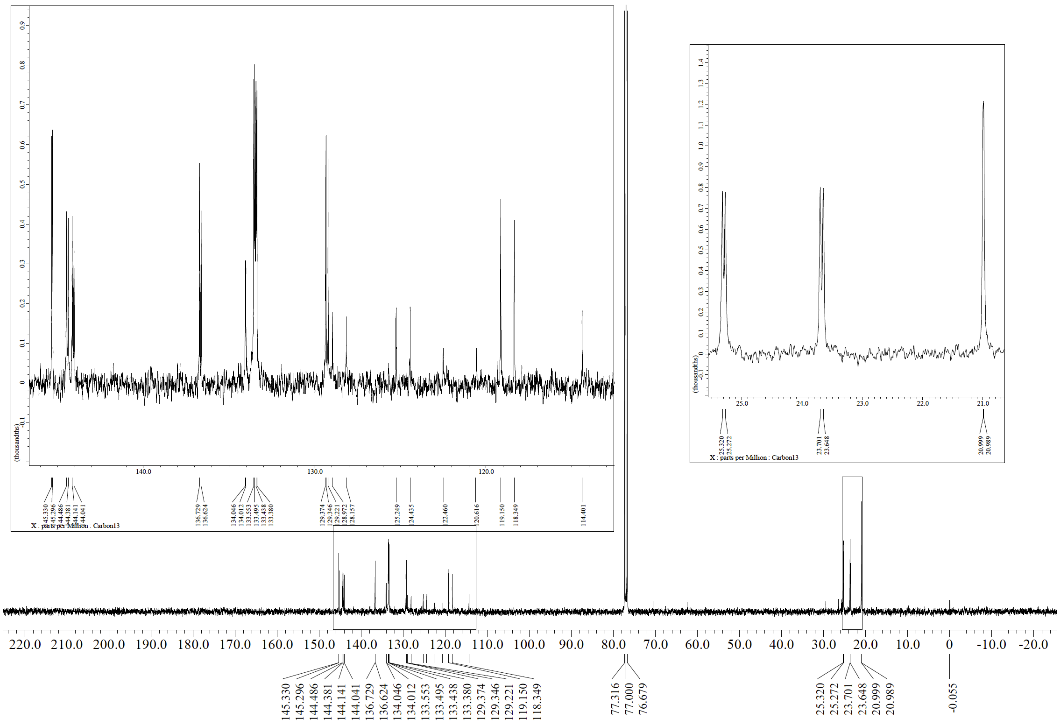

Figure S46. ^{13}C NMR spectrum of **7a**•OTf in $CDCl_3$.

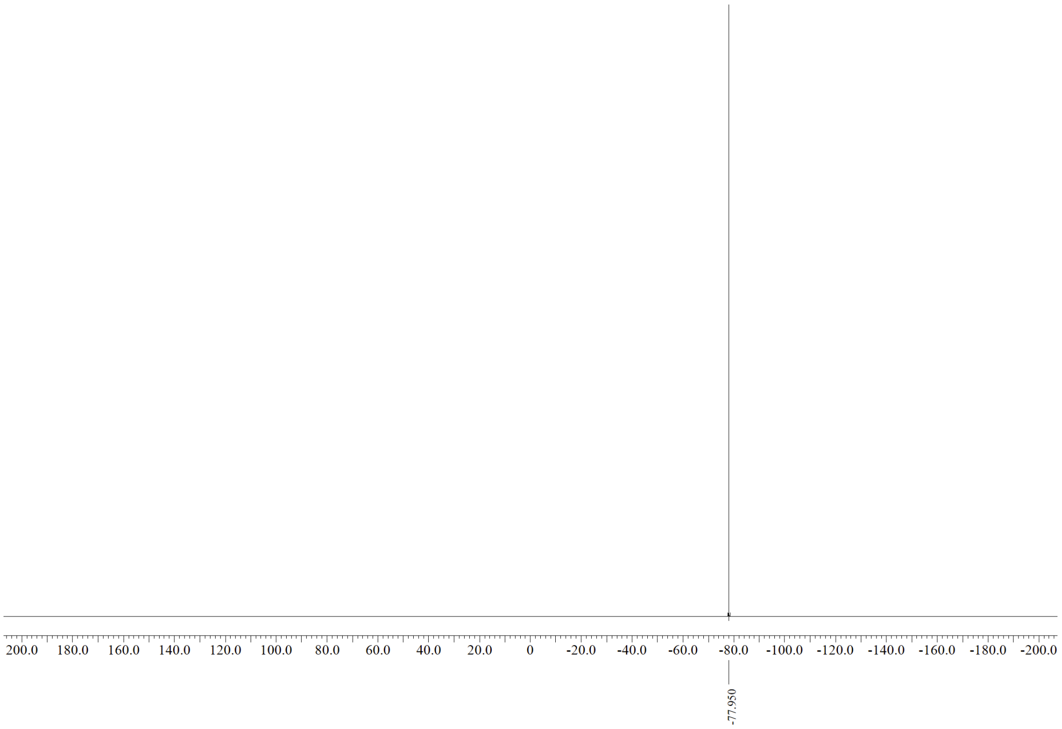

Figure S47. ^{19}F NMR spectrum of **7a**•OTf in $CDCl_3$.

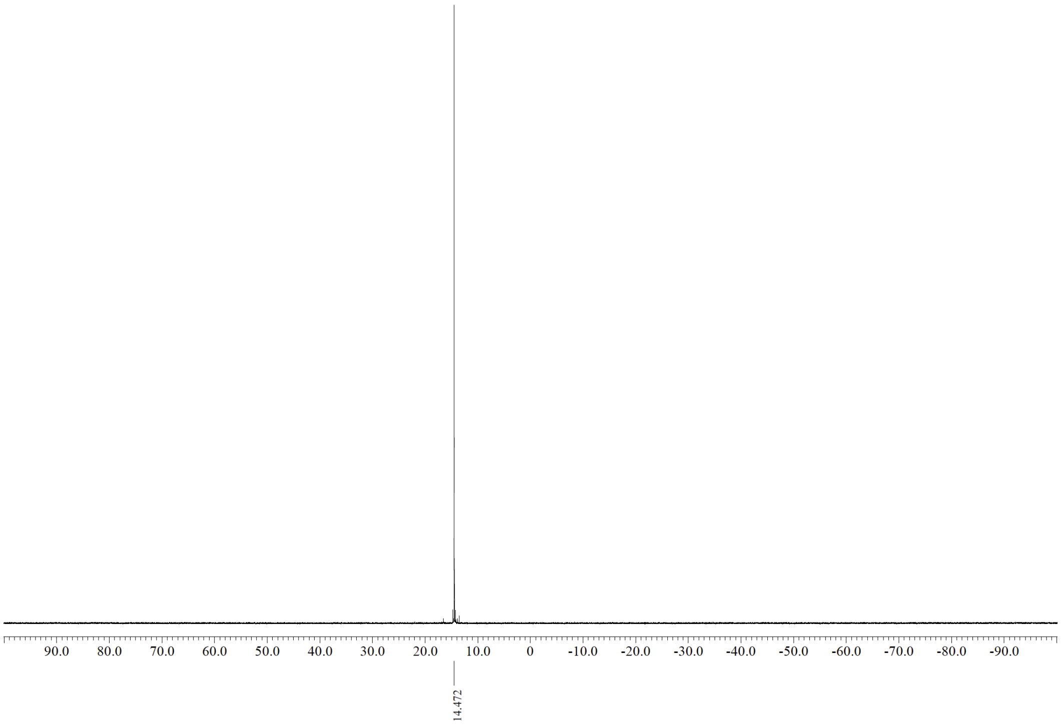

Figure S48. ^{31}P NMR spectrum of **7a**•OTf in $CDCl_3$.

[2-(Phenylthio)phenyl]tri(*o*-tolyl)phosphonium triflate (8a•OTf)

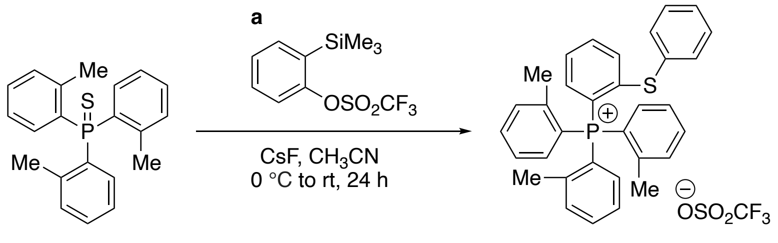

[2-(Phenylthio)phenyl]tri(*o*-tolyl)phosphonium triflate (**8a**•OTf) was synthesized according to the method reported in the literature.[S11]

2-(Trimethylsilyl)phenyl triflate (**a**, 0.233 g, 0.781 mmol, 2.63 eq.) was slowly added to a mixture containing tri(*o*-tolyl)phosphine sulfide (**8**, 0.100 g, 0.297 mmol, 1 eq.) and CsF (0.276 g, 1.82 mmol, 6.11 eq.) in anhydrous CH_3CN (5 mL) at 0 °C. The reaction mixture was allowed to warm to ambient temperature and stirred for 24 h. After adding deionized water to the reaction mixture, the mixture was extracted with ethyl acetate and the organic layer was dried over $MgSO_4$. After the removal of the volatile fractions by evaporation, the residue was purified by column chromatography (SiO_2, CH_2Cl_2 to CH_2Cl_2/methanol = 20/1 vol/vol as an eluent) to give **8a**•OTf as a pale orange powder (0.153 g, 0.240 mmol, 80.6% yield).

Melting point: 210 °C.

^{1}H NMR (400 MHz, $CDCl_3$) δ 1.91 (s, 3H), 1.95 (s, 3H), 1.99 (s, 3H), 6.93–6.95 (2H), 7.25–7.31 (2H), 7.42–7.48 (3H), 7.53–7.89 (14H) ppm.

^{13}C{^{1}H} NMR (100 MHz, $CDCl_3$) δ 22.8–22.9, 23.0 (d, $J_{C–P}$ = 3.9 Hz), 114.3, 115.2, 116.0–116.1, 116.7, 117.6, 120.9 (q, $^1J_{C–F}$ = 318.2 Hz, *C*$F_3SO_3^-$), 127.3, 127.5, 127.6, 127.7, 128.1 (d, $J_{C–P}$ = 13.0 Hz), 128.8 (d, $J_{C–P}$ = 12.5 Hz), 129.0, 129.7, 131.4, 131.6, 133.8, 133.9, 134.0, 134.1, 134.4, 134.5, 134.6, 134.7, 135.3–135.4, 135.5, 135.7, 136.2, 136.4, 136.5, 136.6, 136.8,

143.4 (d, J_{C-P} = 2.4 Hz), 143.5 (d, J_{C-P} = 1.0 Hz), 143.8 (d, J_{C-P} = 8.7 Hz), 144.4 (d, J_{C-P} = 6.7 Hz) ppm.

^{19}F NMR (376 MHz, $CDCl_3$) δ –77.9 (s) ppm.

$^{31}P\{^1H\}$ NMR (121 MHz, $CDCl_3$) δ 23.4 (s) ppm.

HRMS (FAB) *m/z*: $[M - OTf]^+$ calcd for $C_{33}H_{30}PS$, 489.1800; found, 489.1817.

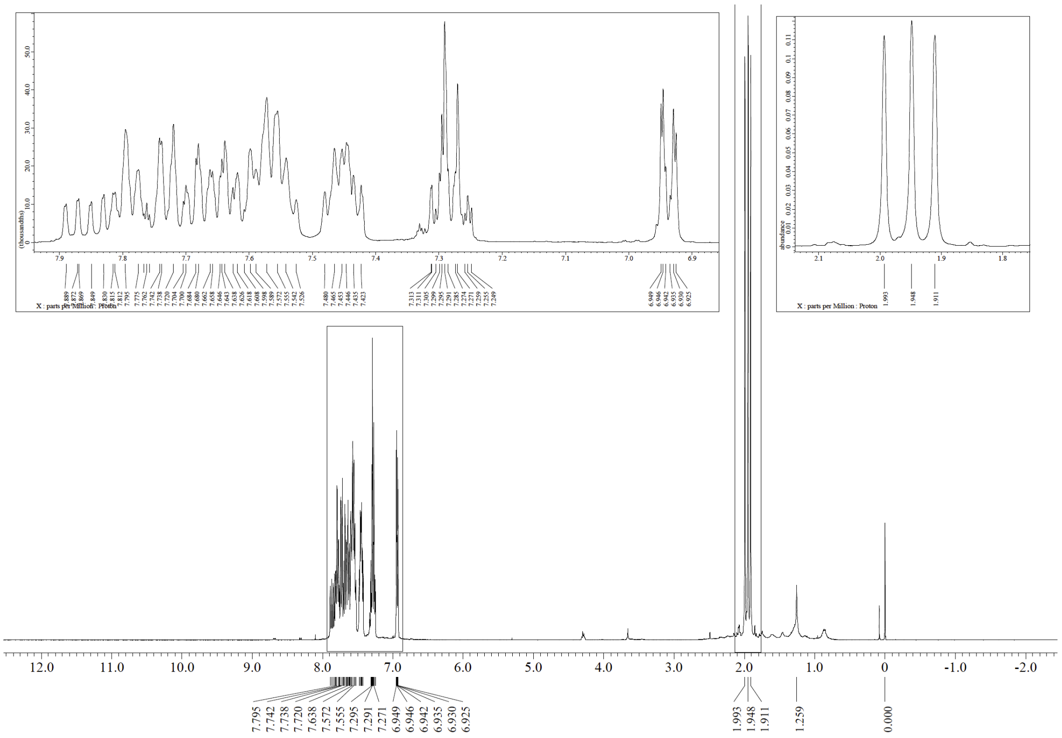

Figure S49. ^{1}H NMR spectrum of **8a**•OTf in $CDCl_3$.

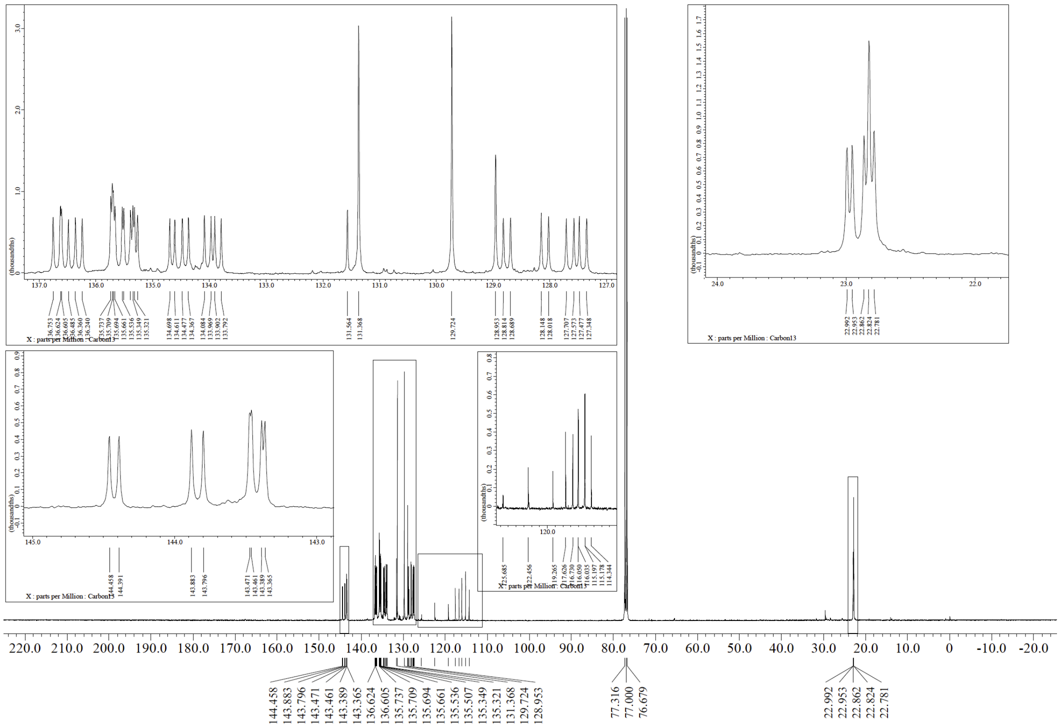

Figure S50. ^{13}C NMR spectrum of **8a**•OTf in $CDCl_3$.

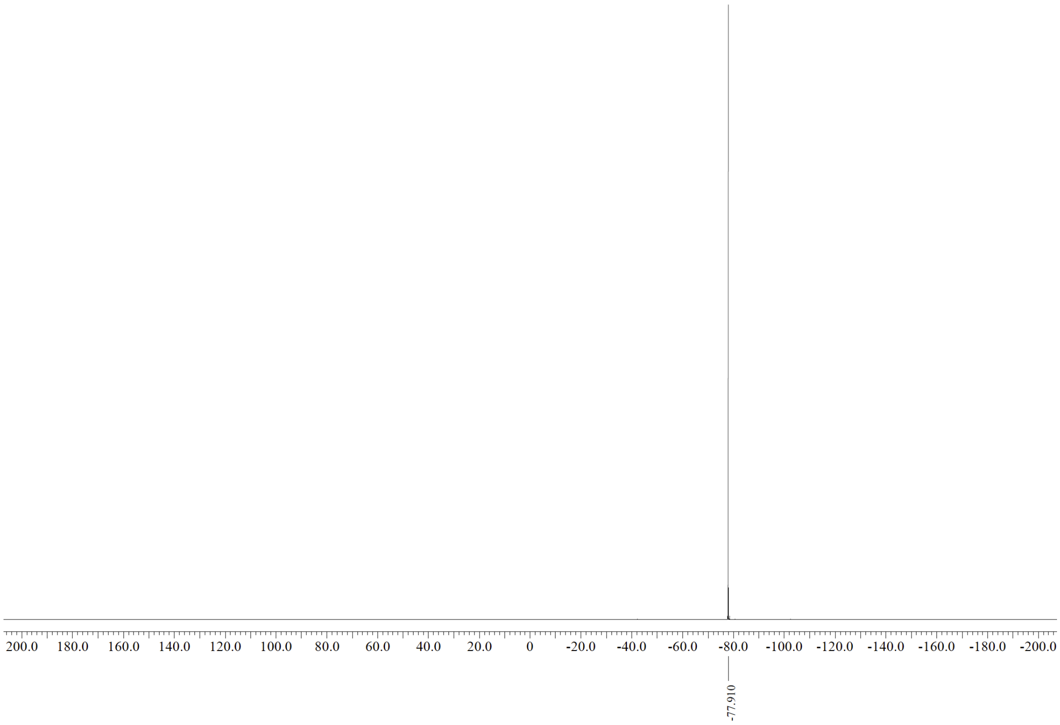

Figure S51. ^{19}F NMR spectrum of **8a**•OTf in $CDCl_3$.

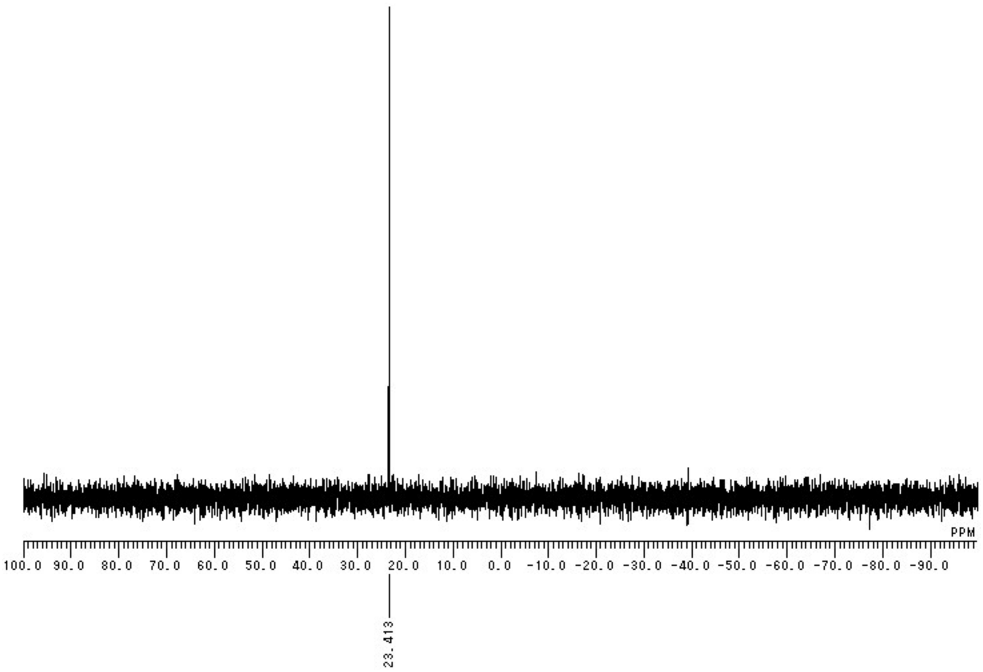

Figure S52. ^{31}P NMR spectrum of **8a**•OTf in $CDCl_3$.

4. Decomposition Product Analysis

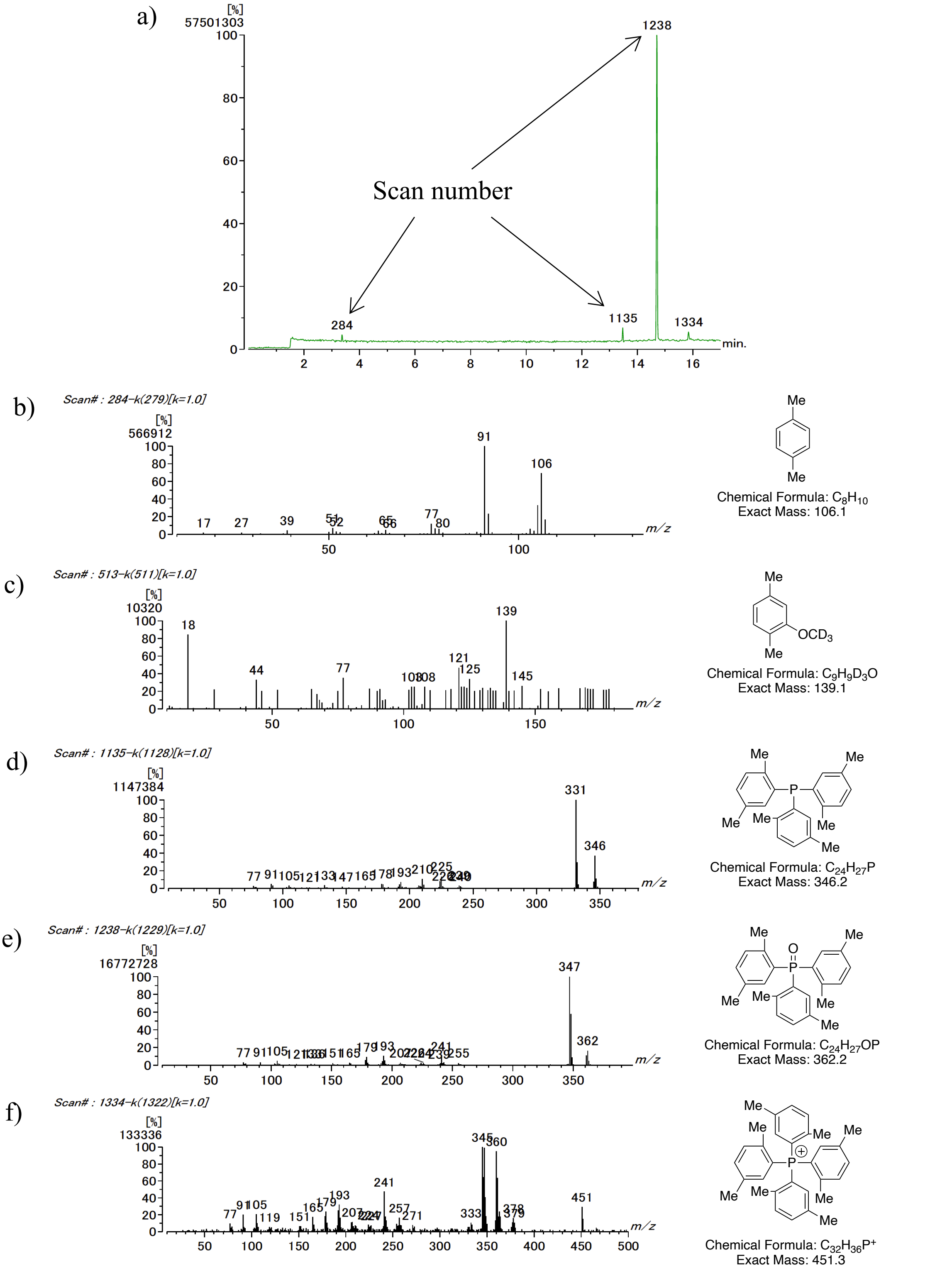

Figure S53. a) GC/EI-MS chart of the sample obtained after heating **3b** at 80 °C for 98 d in 4 M KOH/CD_3OH, b–f) mass spectra of scan numbers of 284, 513, 1135, 1238, and 1334.

5. Relaxation Times of Tetraarylphosphonium Salts

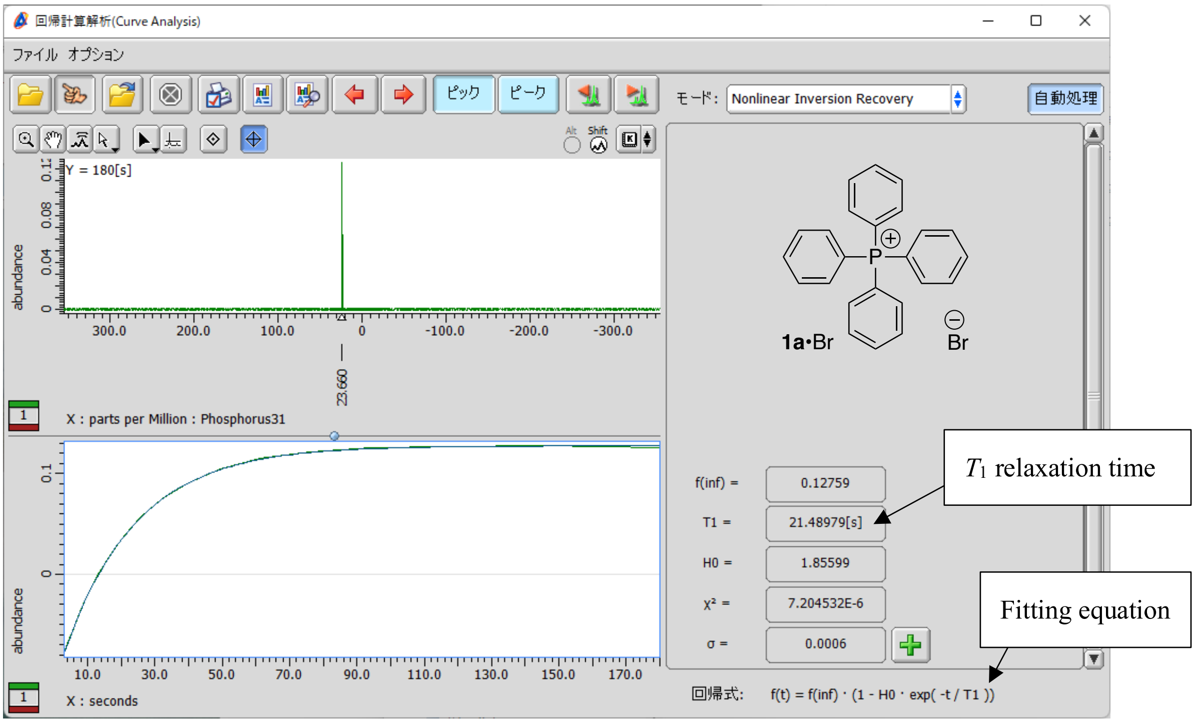

Figure S54. T_1 relaxation experiment of ^{31}P nuclei of **1a**•Br in CD_3OD.

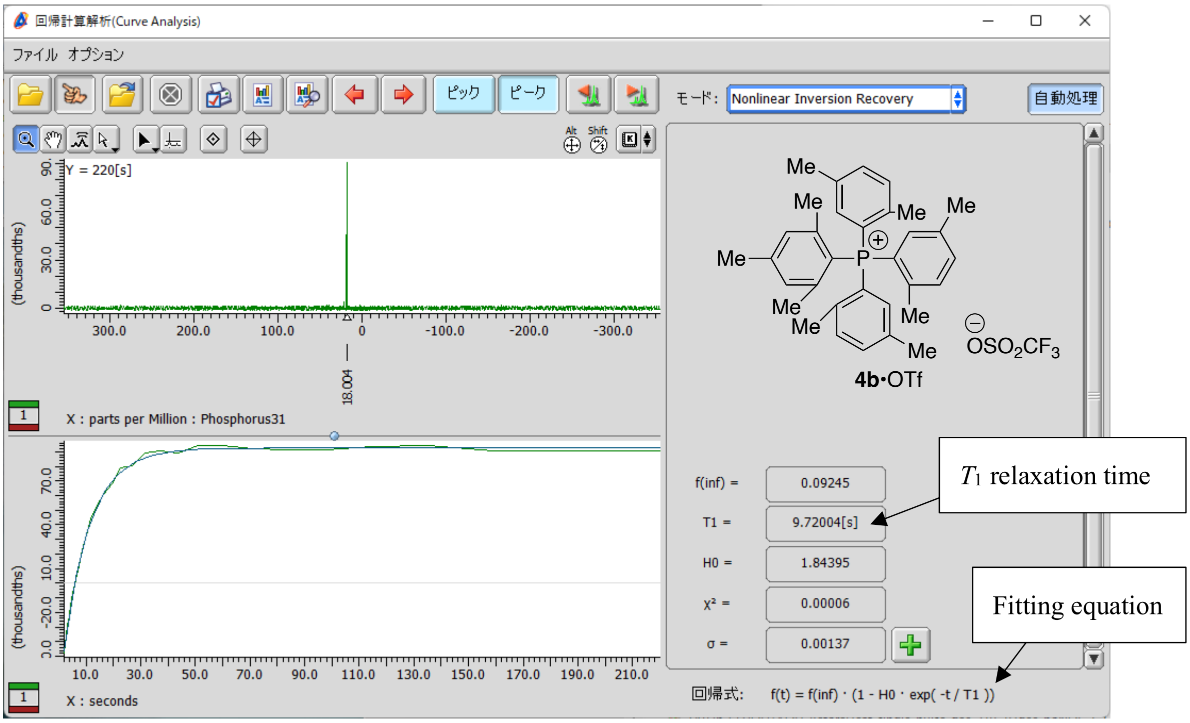

Figure S55. T_1 relaxation experiment of ^{31}P nuclei of **4b**•OTf in CD_3OD.

6. Residual Amounts of Tetraarylphosphonium Cations

Table S1. Residual amount of **2a** in 1 M KOH/CD_3OH at 80 °C.

Structure	Residual amount (%)		
	1 h	7 h	20 h
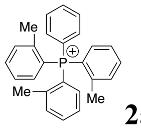 **2a**	84.5	44.4	11.2

Table S2. Residual amount of **2b** in 4 M KOH/CD_3OH at 80 °C.

Structure	Residual amount (%)			
	1 d	2 d	5 d	8 d
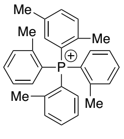 **2b**	74.6	58.2	25.4	12.2

Table S3. Residual amount of **3b** in 4 M KOH/CD_3OH at 80 °C.

Structure	Residual amount (%)											
	1 d	2 d	3 d	4 d	5 d	7 d	9 d	11 d	14 d	18 d	21 d	28 d
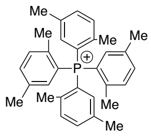 **3b**	100	94.2	86.6	80.8	76.8	66.2	58.2	51.8	44.2	35.7	29.9	23.8

Table S4. Residual amount of **4b** in 4 M KOH/CD_3OH at 80 °C.

Structure	Residual amount (%)						
	1 d	2 d	3 d	4 d	6 d	13 d	27 d
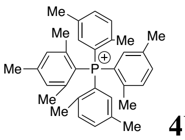 **4b**	91.1	86.2	82.9	80.5	73.2	57.7	48.0

Table S5. Residual amount of **5b** in 4 M KOH/CD_3OH at 80 °C.

Structure	Residual amount (%)						
	1 d	2 d	3 d	4 d	6 d	13 d	27 d
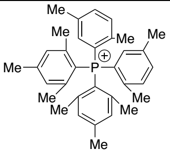 **5b**	96.2	96.2	93.7	87.3	83.5	74.7	60.8

Table S6. Residual amount of **6a** in 1 M KOH/CD_3OH at 80 °C.

Structure	Residual amount (%)				
	1 d	2 d	4 d	6 d	8 d
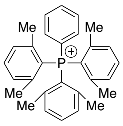 **6a**	88.5	77.9	59.3	45.1	27.4

Table S7. Residual amount of **7a** in 1 M KOH/CD_3OH at 80 °C.

Structure	Residual amount (%)							
	1 d	2 d	4 d	6 d	8 d	10 d	20 d	30 d
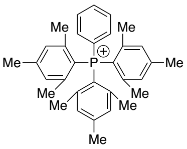 **7a**	95.0	91.7	91.3	90.5	89.6	86.7	83.8	77.2

Table S8. Residual amount of **8a** in 1 M KOH/CD_3OH at 80 °C.

Structure	Residual amount (%)						
	1 d	2 d	3 d	7 d	14 d	21 d	28 d
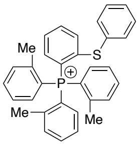 **8a**	98.1	97.5	94.4	92.0	89.5	87.7	85.2

7. Kinetic Plots for Degradation of Tetraarylphosphonium Cations

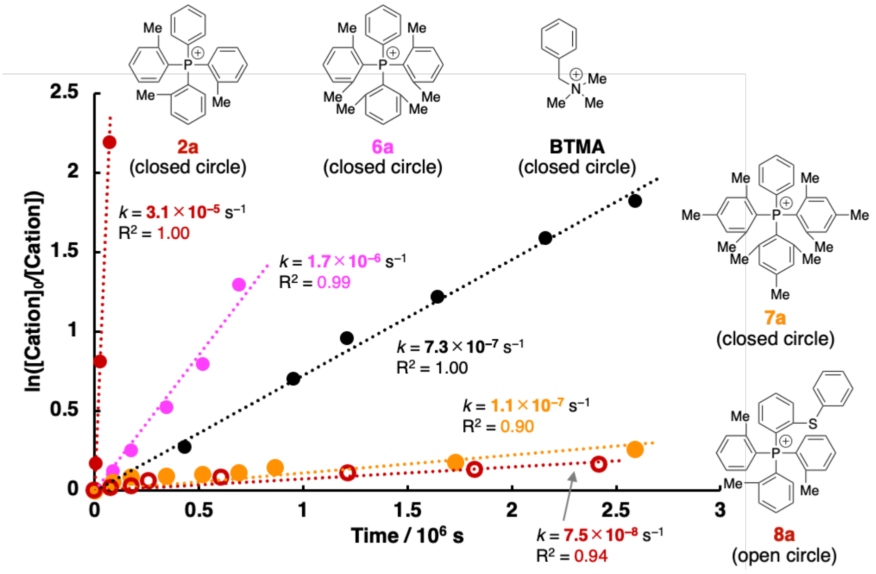

Figure S56. Kinetic plots for degradation of tetraarylphosphonium cations (**2a** and **6a**–**8a**) in 1 M KOH/CD_3OH and **BTMA** in 1 M KOH/CD_3OD at 80 °C. Circles represent measured values. Dotted lines were fitted using $\ln([\mathrm{Cation}]_0/[\mathrm{Cation}]) = kt$, where $[\mathrm{Cation}]_0$, [Cation], and t are the initial concentration of the cations (tetraarylphosphonium or **BTMA**), the concentration of the cations, and time, respectively. R^2 values represent coefficients of determination.

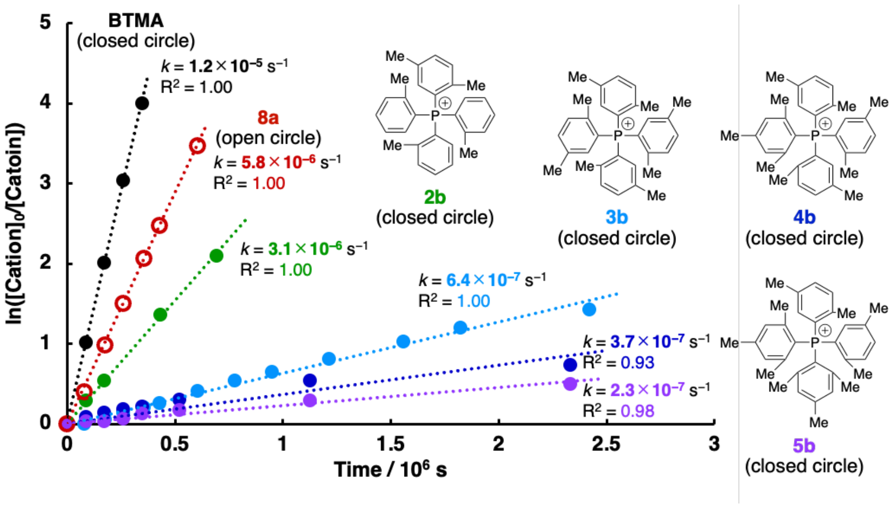

Figure S57. Kinetic plots for degradation of tetraarylphosphonium cations (**2b**–**5b** and **8a**) and **BTMA** in 4 M KOH/CD_3OH at 80 °C. Circles represent measured values. Dotted lines were fitted using $\ln([\mathrm{Cation}]_0/[\mathrm{Cation}]) = kt$, where $[\mathrm{Cation}]_0$, [Cation], and t are the initial concentration of the cations (tetraarylphosphonium or **BTMA**), the concentration of the cations, and time, respectively. R^2 values represent coefficients of determination.

8. Computational Results

Table S9. Natural atomic charges on the cationic phosphorus centers estimated from the DFT calculations (B3LYP/6-31g(d,p) level).

Tetraarylphosphonium	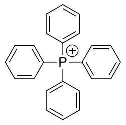 **1a**	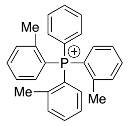 **2a**	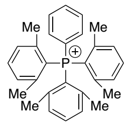 **6a**	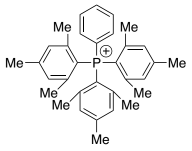 **7a**	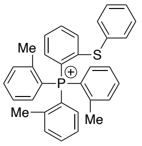 **8a**
Natural atomic charge on P^+	1.720	1.693	1.640	1.646	1.685

Tetraarylphosphonium	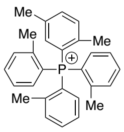 **2b**	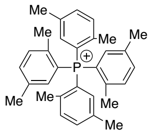 **3b**	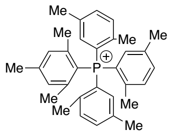 **4b**	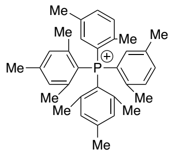 **5b**
Natural atomic charge on P^+	1.685	1.687	1.671	1.654

9. References

[S1] Gaussian 16, Revision C.01, M. J. Frisch, G. W. Trucks, H. B. Schlegel, G. E. Scuseria, M. A. Robb, J. R. Cheeseman, G. Scalmani, V. Barone, G. A. Petersson, H. Nakatsuji, X. Li, M. Caricato, A. V. Marenich, J. Bloino, B. G. Janesko, R. Gomperts, B. Mennucci, H. P. Hratchian, J. V. Ortiz, A. F. Izmaylov, J. L. Sonnenberg, D. Williams-Young, F. Ding, F. Lipparini, F. Egidi, J. Goings, B. Peng, A. Petrone, T. Henderson, D. Ranasinghe, V. G. Zakrzewski, J. Gao, N. Rega, G. Zheng, W. Liang, M. Hada, M. Ehara, K. Toyota, R. Fukuda, J. Hasegawa, M. Ishida, T. Nakajima, Y. Honda, O. Kitao, H. Nakai, T. Vreven, K. Throssell, J. A. Montgomery, Jr., J. E. Peralta, F. Ogliaro, M. J. Bearpark, J. J. Heyd, E. N. Brothers, K. N. Kudin, V. N. Staroverov, T. A. Keith, R. Kobayashi, J. Normand, K. Raghavachari, A. P. Rendell, J. C. Burant, S. S. Iyengar, J. Tomasi, M. Cossi, J. M. Millam, M. Klene, C. Adamo, R. Cammi, J. W. Ochterski, R. L. Martin, K. Morokuma, O. Farkas, J. B. Foresman, D. J. Fox, Gaussian, Inc., Wallingford CT, 2019.

[S2] D. Peña, A. Cobas, D. Pérez, E. Guitián, *Synthesis* **2002**, *2002*, 1454–1458.

[S3] Y. Wang, A. D. Stretton, M. C. McConnell, P. A. Wood, S. Parsons, J. B. Henry, A. R. Mount, T. H. Galow, *J. Am. Chem. Soc.* **2007**, *129*, 13193–13200.

[S4] M. H. Aukland, F. J. T. Talbot, J. A. Fernández-Salas, M. Ball, A. P. Pulis, D. J. Procter, *Angew. Chem. Int. Ed.* **2018**, *57*, 9785–9789.

[S5] A. L. Casalnuovo, T. V. RajanBabu, T. A. Ayers, T. H. Warren, *J. Am. Chem. Soc.* **1994**, *116*, 9869–9882.

[S6] J. Möbus, Q. Bonnin, K. Ueda, R. Fröhlich, K. Itami, G. Kehr, G. Erker, *Angew. Chem. Int. Ed.* **2012**, *51*, 1954–1957.

[S7] M. Wang, F. Nudelman, R. R. Matthes, M. P. Shaver, *J. Am. Chem. Soc.* **2017**, *139*, 14232–14236.

[S8] E. C. Alyea, J. Malito, *Phosphorus, Sulfur, Silicon Relat. Elem.* **1989**, *46*, 175–181.

[S9] J. A. S. Howell, M. G. Palin, P. C. Yates, P. McArdle, D. Cunningham, Z. Goldschmidt, H. E. Gottlieb, D. Hezroni-Langerman, *J. Chem. Soc., Perkin Trans. 2* **1992**, 1769–1775.

[S10] E. Rémond, A. Tessier, F. R. Leroux, J. Bayardon, S. Jugé, *Org. Lett.* **2010**, *12*, 1568–1571.

[S11] C. Lopez-Leonardo, R. Raja, F. Lõpez-Ortiz, M. Ángel Del Águila-Sánchez, M. Alajarin, *Eur. J. Org. Chem.* **2014**, *2014*, 1084–1095.
